# Projecting the clinical burden of chronic kidney disease at the patient level (*Inside CKD*): a microsimulation modelling study

**DOI:** 10.1016/j.eclinm.2024.102614

**Published:** 2024-05-02

**Authors:** Glenn M. Chertow, Ricardo Correa-Rotter, Kai-Uwe Eckardt, Eiichiro Kanda, Avraham Karasik, Guisen Li, Christian Fynbo Christiansen, Panos Stafylas, Stephen G. Holt, Ernst C. Hagen, Juan Jose Garcia Sanchez, Salvatore Barone, Claudia Cabrera, Stephen Nolan, Timothy Coker, Laura Webber, Lise Retat

**Affiliations:** a Stanford University School of Medicine, Stanford Palo Alto, CA 94305, USA; b Instituto Nacional de Ciencias Médicas y Nutrición Salvador Zubirán, Mexico City 14080, Mexico; c Department of Nephrology and Medical Intensive Care, Charité-Universitätsmedizin Berlin, Berlin 10117, Germany; d Medical Science, Kawasaki Medical School, Kurashiki, Okayama 701-0192, Japan; e Maccabi Institute for Research and Innovation, Maccabi Healthcare Services, Tel Aviv 68125, Israel; f Department of Nephrology and Institute of Nephrology, Sichuan Provincial People’s Hospital, School of Medicine, University of Electronic Science and Technology of China, Sichuan Clinical Research Centre for Kidney Diseases, Chengdu 610072, China; g Department of Clinical Epidemiology, Aarhus University Hospital and Aarhus University, Olof Palmes Allé 43-45, Aarhus N DK-8200, Denmark; h HealThink, THERMI GROUP, Steliou Kazantzidi 47 str., Building 1, PC 57 001, PO Box 8121, Thessaloniki, Greece; i SEHA Kidney Care, SKC Central, Abu Dhabi Health Services Co., Al Himam St, Al Mafraq, Abu Dhabi, United Arab Emirates; j Meander Medical Center, Maatweg 3, Amersfoort 3813 TZ, Netherlands; k Global Health Economics, BioPharmaceuticals, AstraZeneca, Academy House, 136 Hills Road, Cambridge CB2 8PA, UK; l Global Medical Affairs, BioPharmaceuticals, AstraZeneca, Gaithersburg, MD 20878, USA; m Real World Science and Analytics, BioPharmaceuticals Medical, AstraZeneca, Gothenburg SE-431 83, Sweden; n Global Medical Affairs, BioPharmaceuticals Medical, AstraZeneca, Cambridge CB2 0AA, UK; o HealthLumen Limited, London EC3N 2PJ, UK

**Keywords:** Burden of disease, Chronic kidney disease, Epidemiology, Microsimulation, Policy

## Abstract

**Background:**

Chronic kidney disease (CKD) is a global concern that presents significant challenges for disease management. Several factors drive CKD prevalence, including primary risk factors, such as type 2 diabetes and hypertension, and an ageing population. *Inside CKD* is an international initiative that aims to raise awareness of the substantial burden incurred by CKD.

**Methods:**

Using a peer-reviewed microsimulation method, the clinical burden of CKD was estimated from 2022 to 2027. Demographic data from the Americas, Europe, and Asia–Pacific/Middle East were used to generate virtual populations and to project the prevalence of CKD, kidney replacement therapy, associated cardiovascular complications, comorbid conditions, and all-cause mortality in the CKD population over the modelled time frame.

**Findings:**

Across the 31 participating countries/regions, the total prevalence of CKD was projected to rise to 436.6 million cases by 2027 (an increase of 5.8% from 2022), with most cases (∼80%) undiagnosed. *Inside CKD* projected a mean of 8859 cases of heart failure, 10,244 of myocardial infarction, and 7797 of stroke per 100,000 patients with CKD by 2027.

**Interpretation:**

The clinical impact of CKD is substantial and likely to increase; the high prevalence of undiagnosed cases and associated complications may benefit from the implementation of health policy interventions that promote screening, earlier diagnosis, and interventions to improve outcomes.

**Funding:**

10.13039/100004325AstraZeneca.

**Research in context**

**Evidence before this study**

A pragmatic literature review was independently performed by local agencies in 31 countries/regions. Local language search terms and exact databases were not recorded, but data were retrieved from available national registries, published literature, and healthcare databases at country and regional levels, which were then assessed by a Scientific Steering Committee. Data were gathered on the prevalence and/or incidence of chronic kidney disease (CKD); the rate of diagnosis; kidney replacement therapy (KRT)—haemodialysis, peritoneal dialysis, and kidney transplantation initiation and maintenance; disease-associated comorbidities of hypertension and type 2 diabetes; cardiovascular complications of heart failure, myocardial infarction, and stroke. Previous studies demonstrate that in addition to complications related to CKD itself and the development of kidney failure, CKD is associated with a substantial risk of cardiovascular complications. CKD has a high prevalence that affects ∼10–15% of the global population and its impact on healthcare are well known, yet a culture of inertia and under-diagnosis undermine effective interventions. There is a lack of available epidemiological data, which undermines the development of sustainable, effective healthcare strategies to improve patient outcomes, which in turn limits the ability of clinicians and policy makers aiming to reduce the burden of disease.

**Added value of this study**

The *Inside CKD* programme produced a simulation across 31 countries/regions, which modelled a virtual population, predicting an estimated 436.6 million cases across the full data set by 2027. This was associated with a projected substantial clinical burden, as evidenced by the cumulative incidence of cardiovascular complications, comorbidities, KRT, and all-cause mortality within the CKD population. The plethora of variables modelled and the global perspective are currently unprecedented in CKD epidemiological research and offer scope for healthcare policy makers to explore projected data specific to their locality.

**Implications of all the available evidence**

*Inside CKD* provides country-specific projections of clinical burden, with the aim of raising awareness of CKD and informing evidence-based policy making. The microsimulation can be programmed to examine the utility and potential impact of various interventions, such as systematic CKD screening programmes.

## Introduction

Chronic kidney disease (CKD) is a debilitating condition that affects 10–15% of the global adult population.^1^ CKD is usually diagnosed using two critical observations, albuminuria (>30 mg/g creatinine) and impaired kidney function, as defined by a glomerular filtration rate (GFR) < 60 mL/min/1.73 m^2^. CKD is further classified by the level of albuminuria (stages A1–3), and the reduction of GFR (stages G3a–5).^2^ As kidney function declines with age, older patients may be vulnerable to developing CKD,^3^ with type 2 diabetes, obesity,^4^^,^^5^ and hypertension^6^ also acting as risk factors. Once diagnosed, CKD progression is associated with adverse cardiovascular events, including stroke,^7^ myocardial infarction,^8^ and heart failure^9^; these conditions may also accelerate the onset of kidney failure. In patients with CKD, comorbid conditions, complications, and major health events all contribute to a lower health-related quality of life, and a higher risk of premature death.^10^^,^^11^

Given the current trajectory of risk factors, CKD is predicted to become an even more common cause of death by 2040 than it is now^12^ with low rates of diagnosis and a general lack of awareness exacerbating the implications of the condition.^13^^,^^14^ Despite a clear need for proactive disease management, there is a paucity of data regarding disease burden that limits optimisation of management strategies. Complementary to real-world data studies, microsimulations create virtual, heterogeneous populations through which clinical outcomes can be modelled. By using a broad range of real-world inputs (including hospital records and national statistics), the course of a disease and associated patient outcomes can be projected.^15^ A particular strength of microsimulation modelling is that the granularity of inputs enables the full complexity of a disease to be considered. Ideally, microsimulations should incorporate multiple inputs with a high level of granularity, and model interventions that can be explored in virtual scenarios.

*Inside CKD* uses a microsimulation methodology to quantify the projected global impact of CKD from 2022 to 2027. Here, we outline changes in the prevalence and distribution of disease stages and kidney replacement therapy (KRT) modalities, assessing the clinical burden for 31 countries/regions in the Americas, Europe, and Asia–Pacific/Middle East.

## Methods

### Study design

*Inside CKD* uses input variables from a range of demographic, epidemiological, and clinical data sets to assess the impact of various health states on disease progression and to project the clinical burden of CKD. A detailed methodology, including sensitivity analyses and validation, has been previously published and the microsimulation has not changed in structure since this description.^16^ The economic burden of CKD, derived from the same microsimulation, is described by Chadban and colleagues in this volume.^17^

This study was conducted in accordance with ethical principles of the Declaration of Helsinki and Good Clinical Practice guidelines. This study did not require informed consent or institutional/ethical review board approval because this is a non-interventional study based on secondary data use.

### Overview

We generated a population of 20 million virtual individuals for each country or region (620 million for the full data set) to allow a starting point for a Monte Carlo procedure. To ensure that the populations were reflective of a given population, we used local demographic data, online databases, published literature, and input from local clinical experts. These virtual individuals then progressed through the microsimulation in 1-year increments from 2022 to 2027. This relatively short time frame allowed for a greater degree of confidence than longer periods, which are associated with increased uncertainty. The time frame also reflected a typical election cycle period, which may be of relevance in the context of healthcare policy, and the slight overlap between present and future data allowed corroboration of the projected baseline findings when the Scientific Steering Committee considered outputs for face validity.

**Fig. 1:**
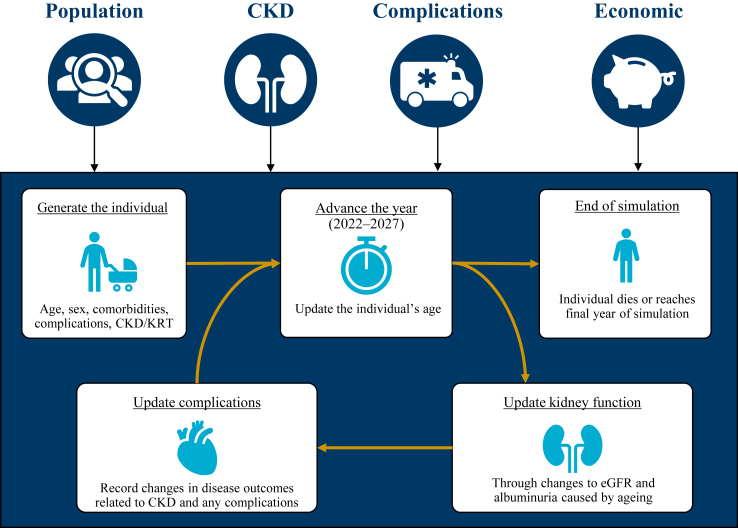
**Overview of the microsimulation modules.** CKD = chronic kidney disease. CV = cardiovascular. eGFR = estimated glomerular filtration rate. KRT = kidney replacement therapy.

At the beginning of each year, the simulation updated the age, estimated glomerular filtration rate (eGFR), and albuminuria values, the related risk of having a cardiovascular complication, and the likelihood of having a comorbidity related to CKD (type 2 diabetes and/or hypertension). These characteristics were also modelled to influence all-cause mortality. Ageing (on a year-to-year basis) increased the risk of complications and all-cause mortality, as well as the likelihood of a new CKD diagnosis or progression of existing disease; in turn, reduced level of GFR and increased level of albuminuria (according to Kidney Disease: Improving Global Outcomes [KDIGO] risk categories)^18^ influenced the relative risk of cardiovascular complications (heart failure, myocardial infarction, and stroke) and all-cause mortality. Accordingly, we updated the health status of each individual each year until estimated death or the study endpoint (Fig. 1). A detailed description of model development, calibration, and statistical drivers has been previously published.^16^

Here, we report the results from the analysis of 31 countries/regions: Australia, Belgium, Brazil, Canada, China, Colombia, Denmark, France, Germany, Greece, Hungary, India, Israel, Italy, Japan, Mexico, Netherlands, Philippines, Poland, Romania, Saudi Arabia, Singapore, South Korea, Spain, Sweden, Taiwan, Thailand, Türkiye, UAE (Emirati population), UK, and USA. These countries were selected based on the availability of epidemiological data (albeit with some variation of completeness) and the agreement of local experts who expressed an interest in participation.

A Scientific Steering Committee comprising local clinical experts provided advice on the modelling approach, conceptualisation, clinical assumptions, validation of data inputs, addressing of data gaps, model calibrations, and interpretation of results from a global and local perspective (Supplementary Table S1).

### Clinical data inputs

Details of the sources for each data input can be found in Supplementary Table S2. These were identified through a robust and extensive literature search within each country and based on local language search terms. Literature search dates varied by country based on data availability but broadly captured publications from the last 10 years, with a cap at 20 years if more recent input sources were scarce.^16^ In brief, we assigned each virtual individual a profile based on a series of input variables that can be broadly grouped as follows: (1) the population module, including baseline demographics such as age and sex; (2) the CKD status module, in which each individual was assigned a CKD status based on eGFR using the CKD-Epidemiology Collaboration equation and on albuminuria as defined by the urine albumin:creatinine ratio, all based on distributions from national and/or regional databases or published literature; the version of the source was appropriate to the data derived from the country or region, and can be assessed in the Supplementary materials detailing input variables; (3) the disease burden module, including the prevalence and/or incidence of comorbid conditions (type 2 diabetes and hypertension), cardiovascular complications (heart failure, myocardial infarction, and stroke), KRT use and the probability of death from all causes; and (4) the health economics module, which included direct CKD costs.

The health economics module was pivotal in determining the financial burden of CKD, and a detailed description of inputs related to costs, as well as the findings from a parallel microsimulation run using the *Inside CKD* model are described in detail by Chadban and colleagues^17^ in this volume.

Because inputs were selected to reflect the specific disease demographics and healthcare systems for a given country or region, there was considerable variation in these parameters, including the threshold of clinical indicators for KRT initiation, the availability of KRT modalities, and the proportion of patients who were able to access treatment. Disease calibrations have been previously described,^16^ and for all countries/regions, KRT prevalence was calibrated against the input data via a regression analysis that extrapolated historical datapoints and compared them with the projected outputs. When country- or region-specific input data were limited, we identified a suitable proxy either from recommendations of key experts or by using a project-specific algorithm (Supplementary Table S2).

To determine disease progression rates, we used annual slopes of eGFR decline for various CKD patient subtypes based on regression analyses from the DISCOVER CKD database.^19^ We assigned disease progression rates to individuals based on clinical characteristics, including the presence of comorbid conditions and cardiovascular complications. The eGFR slopes were assumed to be consistent across modelled countries/regions (see Supplementary Table S2 for input parameters).

### Clinical outputs

We obtained outputs specific to our 31 countries/regions, including the projected prevalence of CKD by GFR stages (G1–G5), age, diagnosis status (diagnosed and undiagnosed), and comorbidity status, as well as that of KRT modality (haemodialysis, peritoneal dialysis, and kidney transplantation), cardiovascular events, and all-cause mortality. These outputs were generated for the end of each year. To account for differences in population size between countries/regions, prevalence data were presented as per 100,000 and/or as percentage change over time, from 2022 to 2027, as appropriate. Prevalence was representative of the total, age-standardised population and was calculated separately for each country. Cumulative incidence was calculated from baseline (2022) up to and including 2027 (i.e. the sum of annual incidences that occurred between 2022 and 2027). Total values for the data set were generated as a straightforward sum of all data. If prevalence was described for the whole data set, weighted mean values for the total data set were used to account for differences in population size.

### Model validation and sensitivity analyses

Although the basic microsimulation model has been previously substantiated,^15^ we undertook a comprehensive evaluation of validity specific to these *Inside CKD* outputs. This approach included validation from key clinical experts and internal checks. A summary of the model validation has been previously published.^16^ In brief, the validation strategy was conducted in accordance with the International Society for Pharmacoeconomics and Outcomes Research and the Society for Medical Decision Making (ISPOR-SMDM) task force, and comprised four different aspects of validation: external validation, internal validation, cross-validation, and face validity.^20^ Here, we report external validation for the specific countries/regions for which data were available (Supplementary Table S3). The Scientific Steering Committee provided face validity assessments of all included input data and endpoints reported here, including the input data and outputs for type 2 diabetes and hypertension.

A sensitivity analysis was conducted for each country/region on diagnosis rates for each CKD stage in which prevalence of CKD was projected using a 10% increase and a 10% decrease in the total weighted diagnosis rate. The data inputs used for diagnosis rates are provided in Supplementary Table S2. Diagnosis rate input data were not available for many countries/regions, in these cases, proxy values were utilised, and the sensitivity analysis was performed to assess the robustness of the model against changes in diagnostic rates.

### Role of the funding source

The funder of the study was involved in the study design, data analysis, data interpretation and writing of the report. The funder had no role in data collection, which were conducted by HealthLumen. Authors (LR and LW) checked and corroborated the data. All authors had full access to all data and the corresponding author had final responsibility for the decision to submit for publication.

## Results

### Baseline demographics of the virtual population

Each virtual population was scaled to its given regional population, with baseline demographic characteristics shown in Table 1 and Table 2. Baseline drivers of CKD are provided in Supplementary Table S4. Sex distribution was consistent across regions, with the exception of the UAE, where the proportion of men was higher (Table 1). The proportion of persons aged 65 years and older varied widely (range 3.6–28.9%) (Table 1). The overall proportion of persons with CKD (diagnosed and undiagnosed) in the virtual population varied between 5.5% and 18.8% (Table 2). The proportion of persons with diagnosed CKD also varied between 0.8% and 5.9% (Table 2). Among those with CKD, an overall weighted mean of 26.3% were diagnosed with type 2 diabetes, and 60.0% were diagnosed with hypertension (Table 2). At baseline, in 2022, all participating countries/regions showed a high prevalence of diagnosed CKD (range 846–5927 per 100,000), but an even greater prevalence of undiagnosed CKD (range 4137–12,860) (Supplementary Fig. S1, Table 3).

**Table 1:** Demographic summary for each virtual population.

Region	Country	Population size (number of people)		Sex, % (2022)		Age, % (2022)				Percentage increase in population ≥65 years old (2022–2027)
		2022	2027	Female	Male	0–17 years	18–34 years	35–64 years	≥65 years	
Americas	Brazil	215,353,588	221,142,806	50.9	49.1	24.5	26.6	38.6	10.3	22.5
	Canada	38,388,416	39,939,039	50.3	49.7	18.9	22.2	39.9	19.0	17.7
	Colombia	51,512,766	52,530,216	50.9	49.1	26.2	28.3	35.8	9.7	22.6
	Mexico	131,562,775	137,608,574	51.1	48.9	30.2	27.8	33.9	8.1	20.9
	USA	334,805,268	344,100,699	50.5	49.5	22.0	23.3	37.3	17.4	14.0
Europe	Belgium	11,668,276	11,817,128	50.4	49.6	20.3	20.3	39.5	19.9	11.0
	Denmark	5,834,952	5,942,672	50.3	49.7	19.7	22.0	37.8	20.6	7.9
	France	65,584,514	66,323,948	51.6	48.4	21.0	19.5	38.1	21.4	8.9
	Germany	83,883,587	83,346,306	50.5	49.5	16.9	19.2	41.6	22.3	9.3
	Greece	10,316,641	10,054,798	50.9	49.1	16.3	17.5	43.2	23.0	6.5
	Hungary	9,606,252	9,447,725	52.4	47.6	17.5	19.9	41.6	21.0	2.7
	Italy	60,262,779	59,555,936	51.2	48.8	15.5	17.5	43.1	23.9	7.9
	Netherlands	17,211,449	17,380,060	50.1	49.9	18.8	20.9	39.4	20.9	12.0
	Poland	37,739,779	37,314,983	51.6	48.4	18.1	19.7	42.2	20.0	11.0
	Romania	19,031,330	18,590,731	51.4	48.6	18.7	19.4	41.9	20.1	2.1
	Spain	47,865,714	48,494,124	51.0	49.0	17.3	18.3	44.3	20.2	11.9
	Sweden	10,218,972	10,488,223	49.9	50.1	21.0	21.2	37.2	20.6	7.0
	Türkiye	85,561,976	87,612,953	50.6	49.4	28.0	26.1	36.4	9.6	20.2
	UK	68,497,913	69,771,961	50.5	49.5	21.0	21.2	38.8	19.0	9.3
Asia–Pacific/Middle East	Australia	26,068,793	27,406,327	50.2	49.8	23.0	22.3	37.9	16.8	15.2
	China	1,448,471,404	1,461,797,638	48.7	51.3	20.9	22.7	43.6	12.8	18.4
	India	1,406,631,781	1,469,338,564	48.0	52.0	30.8	28.9	33.3	7.0	19.4
	Israel	8,922,893	9,582,849	50.2	49.8	32.4	23.3	31.6	12.7	12.2
	Japan	125,584,839	122,754,999	51.2	48.8	14.9	16.5	39.7	28.9	1.5
	Philippines	112,508,991	119,632,644	49.8	50.2	34.8	29.2	30.0	5.9	24.9
	Saudi Arabia	35,844,913	38,115,319	42.2	57.8	28.5	26.5	41.2	3.8	39.4
	Singapore	5,943,551	6,158,533	47.7	52.3	14.9	22.5	47.4	15.2	35.8
	South Korea	51,329,905	51,293,519	50.0	50.0	14.8	20.8	47.0	17.4	26.2
	Taiwan	23,888,600	23,997,588	50.4	49.6	15.2	21.3	46.2	17.3	22.4
	Thailand	70,078,198	70,394,102	51.4	48.6	19.6	22.6	43.6	14.2	24.2
	UAE Emirati^a^	1,604,749	1,749,670	23.3	76.7	31.0	29.8	35.7	3.6	122.4

CKD = chronic kidney disease.

The virtual population comprised 20 million individuals, and was scaled to the given country or region population. Note that the increase in population is given as the percentage of projected change.

a These demographic data were generated for both the Emirati and non-Emirati populations, but it should be noted that the relatively high proportion of males reflects the predominantly male expatriate subgroup in the non-Emirati population.

**Table 2:** CKD demographics for each virtual population at baseline (2022).

Region	Country	CKD stage, % (diagnosed and undiagnosed)						CKD, all stages, %		Comorbidity prevalence in the CKD population, %	
		1	2	3a	3b	4	5	Diagnosed and undiagnosed	Diagnosed	Type 2 diabetes	Hyper-tension
Americas	Brazil	1.57	1.73	4.70	1.55	0.16	0.02	9.73	2.96	36.0	78.6
	Canada	3.10	2.39	5.59	1.87	0.35	0.05	13.34	3.88	32.2	56.9
	Colombia	1.74	1.19	3.11	0.53	0.10	0.01	6.68	1.71	33.1	77.4
	Mexico	4.46	3.19	2.24	0.73	0.18	0.14	10.93	2.43	43.1	79.8
	USA	5.33	3.81	4.31	1.32	0.11	0.02	14.89	3.40	29.5	62.9
Europe	Belgium	2.97	2.42	5.65	2.20	0.81	0.13	14.19	4.28	19.9	55.8
	Denmark	3.05	2.48	6.06	1.94	0.33	0.04	13.89	4.05	19.4	55.4
	France	3.58	2.33	3.38	1.09	0.33	0.11	10.82	3.06	19.9	52.7
	Germany	8.33	1.64	2.01	0.61	0.11	0.01	12.71	2.08	20.0	48.1
	Greece	1.63	1.98	6.32	1.37	0.25	0.03	11.58	3.63	19.6	57.8
	Hungary	3.11	2.50	5.95	1.77	0.27	0.03	13.63	3.53	27.8	72.6
	Italy	2.06	1.28	2.46	0.63	0.19	0.09	6.72	1.78	35.1	77.8
	Netherlands	3.09	2.78	4.67	1.39	0.25	0.03	12.19	3.37	19.7	55.4
	Poland	2.05	2.54	4.57	1.33	0.22	0.02	10.73	3.17	20.0	57.3
	Romania	1.39	1.22	3.75	0.59	0.07	0.01	7.02	2.06	27.0	72.0
	Spain	1.65	1.82	5.65	1.32	0.24	0.04	10.73	3.39	31.9	77.2
	Sweden	2.29	1.92	3.51	1.10	0.28	0.17	9.26	2.49	33.8	62.8
	Türkiye	5.32	2.72	1.98	1.40	0.23	0.08	11.74	2.50	20.9	52.8
	UK	3.00	2.33	5.68	1.96	0.36	0.05	13.38	3.94	19.8	55.3
Asia–Pacific/Middle East	Australia	3.53	2.22	2.43	0.54	0.14	0.06	8.92	2.03	20.6	52.5
	China	6.13	0.96	1.17	0.21	0.05	0.02	8.54	1.10	20.8	49.9
	India	3.54	0.70	0.84	0.28	0.05	0.05	5.46	0.85	30.0	65.1
	Israel	7.19	2.47	2.24	0.80	0.36	0.07	13.13	2.32	37.7	44.7
	Japan	2.18	6.33	5.43	4.31	0.50	0.04	18.79	5.93	19.9	54.6
	Philippines	1.88	0.93	2.15	0.38	0.09	0.03	5.47	1.33	22.3	66.6
	Saudi Arabia	4.13	2.63	3.86	1.03	0.34	0.30	12.28	3.21	28.2	60.6
	Singapore	5.86	3.84	4.89	0.94	0.27	0.09	15.89	3.58	20.7	53.6
	South Korea	4.59	2.83	2.58	0.62	0.16	0.02	10.81	2.31	20.7	52.5
	Taiwan	4.54	2.75	2.54	0.61	0.16	0.02	10.62	2.26	42.0	66.2
	Thailand	2.43	3.56	8.64	2.24	0.39	0.21	17.46	4.90	20.0	57.5
	UAE Emirati	3.94	2.51	3.53	0.81	0.31	0.25	11.35	2.89	28.0	60.8

CKD = chronic kidney disease.

The UAE comprised the Emirati population and the expatriate population. Only the Emirati populations are considered in the results. The virtual population comprised 20 million individuals, and was scaled to the given country or region population.

**Table 3:** Prevalence of diagnosed and undiagnosed CKD cases, per 100,000 of the national population.

Diagnosed CKD prevalence per 100,000 people (including KRT)									
Year	Country/region	CKD stage						Total	% Change 2022–2027
		1	2	3a	3b	4	5		
Americas									
2022	Brazil	67	469	1379	888	139	20	2963	7.8
2027		74	430	1677	783	176	55	3193	
2022	Canada	133	652	2057	741	256	46	3885	1.1
2027		144	598	2246	646	203	88	3926	
2022	Colombia	76	322	915	304	85	11	1713	22.3
2027		88	295	1365	249	68	31	2095	
2022	Mexico	190	870	658	418	158	131	2425	0.8
2027		215	745	924	364	136	63	2446	
2022	USA	229	1036	1268	756	98	17	3404	−0.3
2027		252	896	1373	663	162	48	3394	
Europe									
2022	Belgium	128	657	2081	871	595	131	4463	−5.3
2027		141	592	2280	711	340	164	4228	
2022	Denmark	134	673	2233	766	243	37	4087	−2.6
2027		151	601	2494	529	137	69	3981	
2022	France	155	635	1244	431	241	111	2817	7.3
2027		173	528	1647	356	217	102	3023	
2022	Germany	359	445	739	243	83	14	1884	11.1
2027		349	448	991	209	73	24	2093	
2022	Greece	92	163	2328	544	186	32	3345	11.3
2027		106	148	2865	431	114	60	3723	
2022	Hungary	136	683	1749	1021	242	28	3858	−7.1
2027		153	599	2021	640	119	54	3585	
2022	Italy	88	349	724	362	166	89	1778	6.3
2027		94	288	976	352	118	62	1890	
2022	Netherlands	134	754	1720	551	181	28	3369	7.7
2027		151	676	2242	392	116	50	3628	
2022	Poland	89	688	1682	523	159	24	3165	7.5
2027		107	641	2177	345	89	42	3401	
2022	Romania	61	332	1384	231	49	6	2063	33.2
2027		69	262	2160	170	48	39	2748	
2022	Spain	71	493	2084	522	180	37	3387	9.5
2027		79	439	2456	525	141	69	3709	
2022	Sweden	458	385	702	554	222	165	2486	−3.6
2027		517	321	911	381	141	126	2398	
2022	Türkiye	230	740	729	555	173	75	2502	0.4
2027		253	667	1034	348	161	49	2512	
2022	UK	128	634	2086	776	267	48	3940	−3.2
2027		140	567	2202	641	188	75	3814	
Asia–Pacific/Middle East									
2022	Australia	152	604	896	216	103	58	2030	7.0
2027		168	516	1141	219	82	46	2171	
2022	China	267	263	432	84	37	16	1098	−1.6
2027		270	248	455	80	19	8	1081	
2022	India	151	190	310	111	35	48	846	−2.5
2027		157	172	401	67	19	8	825	
2022	Israel	401	202	792	527	329	67	2319	−9.4
2027		420	185	902	375	160	60	2101	
2022	Japan	94	1717	1999	1710	370	36	5927	9.9
2027		111	1518	3179	1122	487	97	6514	
2022	Philippines	276	137	713	126	48	30	1331	6.4
2027		295	117	820	134	28	22	1417	
2022	Saudi Arabia	179	720	1131	593	302	284	3210	15.0
2027		202	673	1441	687	333	355	3692	
2022	Singapore	859	564	1617	312	141	89	3583	10.6
2027		920	546	1888	401	113	95	3964	
2022	South Korea	197	771	951	246	119	22	2305	32.3
2027		212	699	1628	286	162	64	3051	
2022	Taiwan	196	749	935	242	114	21	2258	28.8
2027		208	658	1574	266	146	56	2908	
2022	Thailand	356	523	2864	743	205	206	4897	11.2
2027		407	472	3142	895	263	269	5448	
2022	UAE (Emirati)	173	678	1051	471	279	235	2888	20.1
2027		205	724	1563	457	303	217	3469	
2022	UAE (Expatriate)	192	100	91	24	5	7	419	28.8
2027		195	102	209	22	9	3	540	

Undiagnosed CKD prevalence per 100,000 people (including KRT)									
Year	Country/region	CKD stage						Total	% Change 2022–2027
		1	2	3a	3b	4	5		
Americas									
2022	Brazil	1503	1263	3318	661	17	1	6763	10.3
2027		1664	1149	4042	581	21	2	7459	
2022	Canada	2963	1743	3535	1125	94	0	9460	2.8
2027		3216	1598	3852	983	74	0	9723	
2022	Colombia	1668	867	2194	227	10	0	4966	12.0
2027		1859	793	2714	184	8	1	5560	
2022	Mexico	4265	2322	1581	309	19	6	8502	7.9
2027		4725	1947	2212	268	16	3	9171	
2022	USA	5098	2774	3043	561	12	1	11,488	1.9
2027		5526	2373	3294	493	19	2	11,708	
Europe									
2022	Belgium	2847	1758	3571	1332	215	0	9723	1.1
2027		3128	1577	3917	1082	123	0	9827	
2022	Denmark	2913	1803	3824	1172	89	0	9801	−2.0
2027		3228	1605	3910	812	49	0	9605	
2022	France	3429	1692	2134	657	85	2	7999	4.9
2027		3795	1407	2578	535	72	2	8389	
2022	Germany	7967	1193	1269	366	30	0	10,825	1.4
2027		7719	1206	1707	315	26	0	10,972	
2022	Greece	1535	1820	3989	825	67	0	8236	3.7
2027		1766	1642	4443	652	41	0	8544	
2022	Hungary	2971	1819	4198	753	29	1	9771	−1.0
2027		3252	1590	4341	472	14	2	9671	
2022	Italy	1974	933	1740	267	20	4	4938	11.6
2027		2118	771	2345	259	14	3	5509	
2022	Netherlands	2952	2021	2947	840	65	0	8825	4.20
2027		3263	1812	3476	599	42	0	9192	
2022	Poland	1961	1856	2889	802	57	0	7565	4.6
2027		2348	1724	3285	526	32	0	7916	
2022	Romania	1326	889	2361	358	19	0	4952	14.6
2027		1468	703	3225	262	18	0	5676	
2022	Spain	1582	1329	3569	797	65	0	7342	9.0
2027		1764	1177	4214	794	52	0	8001	
2022	Sweden	1830	1537	2803	548	54	0	6772	3.4
2027		2060	1277	3256	377	32	0	7002	
2022	Türkiye	5094	1982	1254	847	62	0	9239	5.7
2027		5604	1789	1778	534	56	0	9761	
2022	UK	2872	1693	3589	1184	98	0	9437	0.3
2027		3125	1513	3788	974	69	0	9468	
Asia–Pacific/Middle East									
2022	Australia	3374	1614	1535	327	37	1	6888	6.5
2027		3659	1364	1953	331	29	1	7338	
2022	China	5862	701	741	128	13	0	7446	0.9
2027		5950	661	775	123	7	0	7516	
2022	India	3389	509	531	168	12	0	4610	3.4
2027		3518	455	684	102	7	0	4766	
2022	Israel	6792	2263	1450	275	31	0	10,811	1.5
2027		7047	2074	1644	197	16	0	10,978	
2022	Japan	2086	4609	3434	2599	133	0	12,860	7.9
2027		2452	4071	5464	1714	174	0	13,875	
2022	Philippines	1608	792	1440	254	44	0	4137	5.3
2027		1721	681	1660	270	25	0	4356	
2022	Saudi Arabia	3955	1907	2725	437	37	12	9074	12.2
2027		4404	1754	3455	510	40	16	10,179	
2022	Singapore	5005	3279	3271	628	126	0	12,309	7.7
2027		5358	3172	3812	811	102	0	13,255	
2022	South Korea	4394	2056	1633	376	44	0	8503	16.2
2027		4726	1867	2796	431	59	0	9879	
2022	Taiwan	4345	2003	1604	370	41	0	8362	14.0
2027		4606	1762	2703	408	54	0	9532	
2022	Thailand	2071	3039	5777	1497	183	0	12,568	7.3
2027		2367	2745	6337	1802	235	0	13,486	
2022	UAE (Emirati)	3764	1827	2482	343	32	10	8457	11.5
2027		4169	1890	3012	318	31	9	9429	
2022	UAE (Expatriate)	4199	271	222	18	1	0	4711	4.8
2027		4272	268	377	16	1	0	4934	

CKD = chronic kidney disease. KRT = kidney replacement therapy.

Note: individual values have been rounded to whole numbers. Slight discrepancies may occur between the total sum reported as a result.

### Prevalence of CKD

**Fig. 2:**
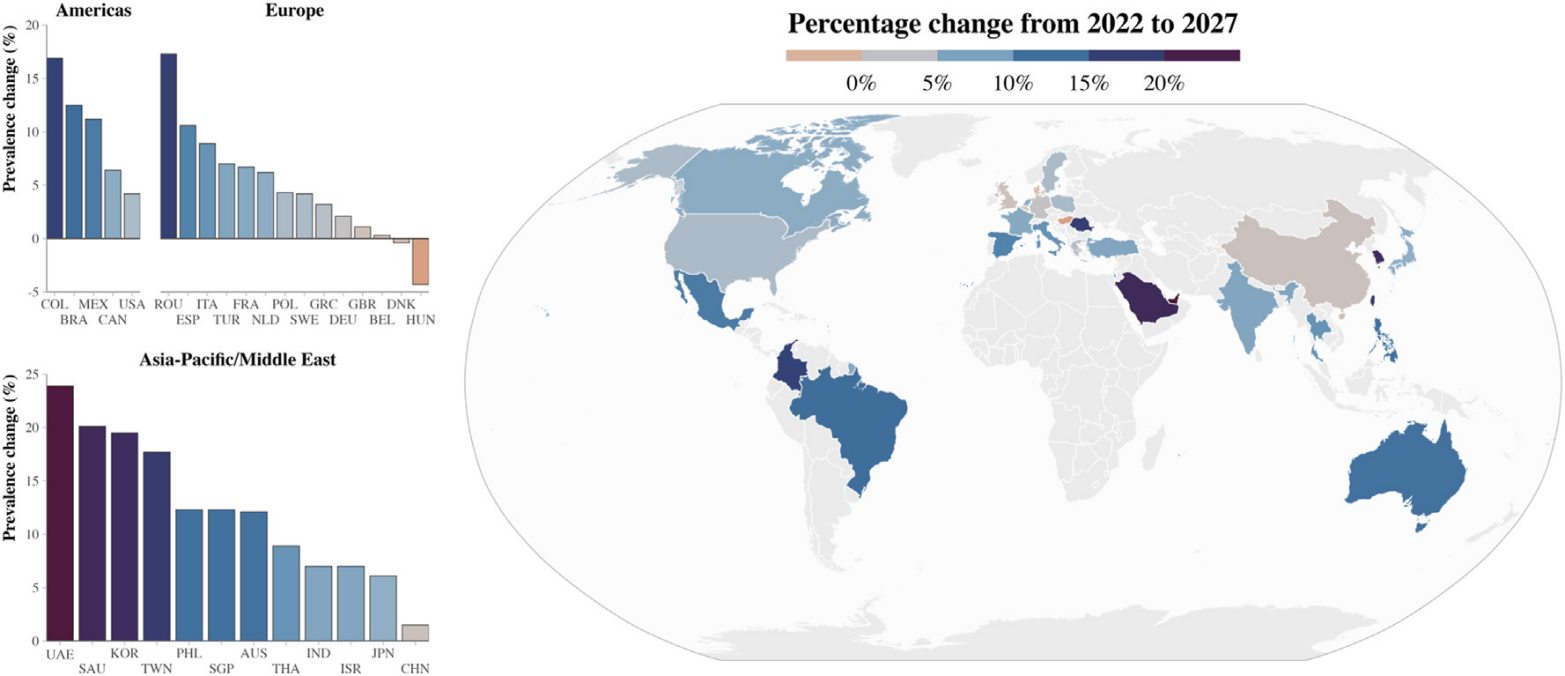
**Projected percentage change of CKD prevalence in each population 2022–2027.** Notes: The UAE has a large and diverse Expatriate population with a different CKD profile; only the Emirati population has been presented here. CKD = chronic kidney disease.

Across the complete data set, a total of 436.6 million cases were projected by 2027 (an increase of 5.8% from 2022) with all countries showing an increase in absolute numbers except for Hungary and Denmark (Fig. 2, Supplementary Table S5). Assuming no changes to diagnostic techniques and screening, undiagnosed individuals were projected to constitute 80.0% of the CKD population across data sets in 2027 (range: 68.1–87.4%) (Table 3).

### Cardiovascular complications in the CKD population

**Fig. 3:**
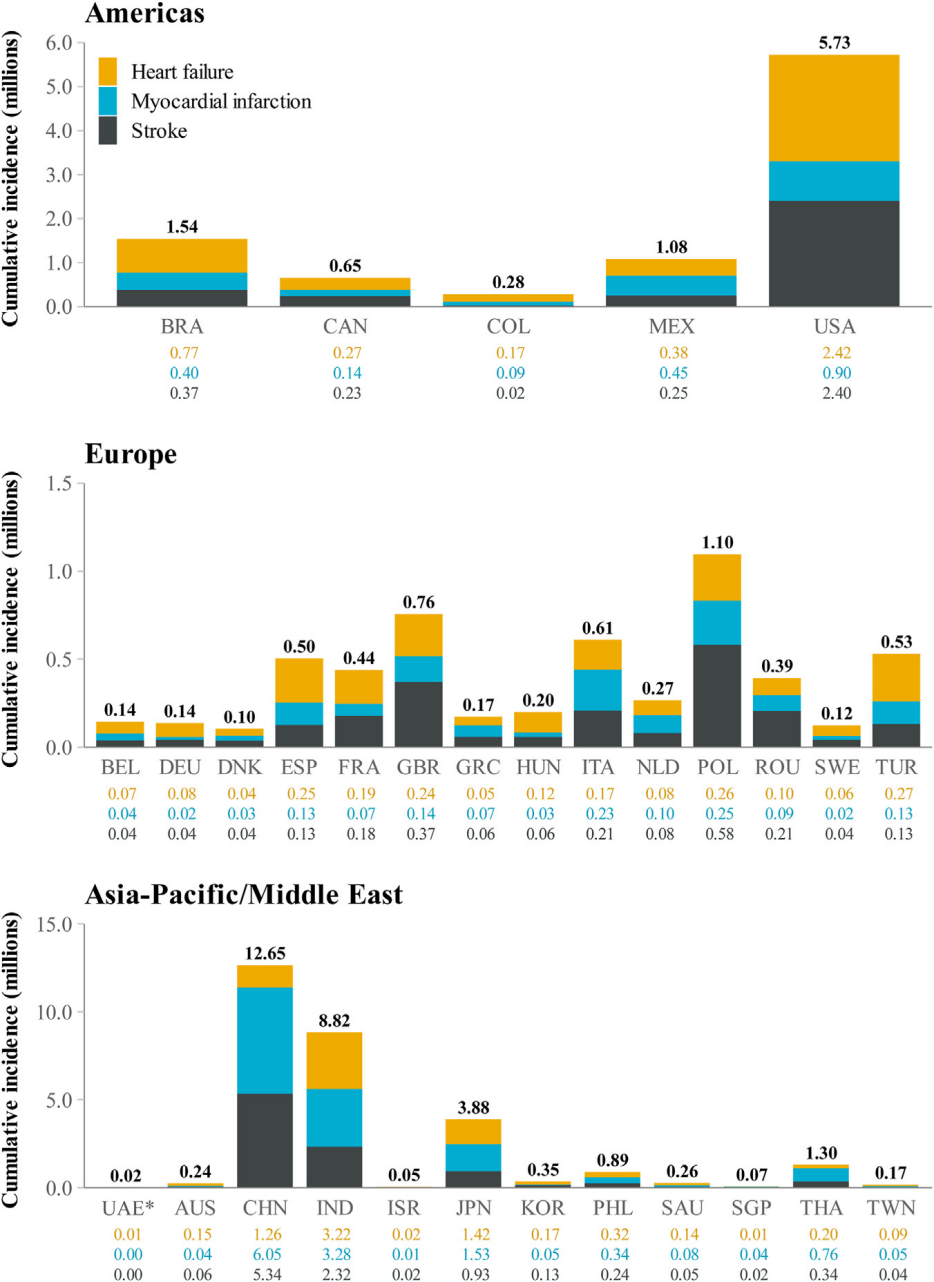
**Projected cumulative incidence (2022–2027) of cardiovascular complications in patients with diagnosed CKD.** Note: The UAE has a large and diverse Expatriate population with a different CKD profile; only the Emirati population has been provided here. Individual values have been rounded to two decimal places. Slight discrepancies may occur between the total sum reported as a result. CKD = chronic kidney disease. ∗UAE cumulative incidence where values shown are <0.01 million: Heart failure, 7328; Myocardial infarction, 4151; Stroke, 4183.

Fig. 3 illustrates the cumulative incidence of cardiovascular complications in patients diagnosed with CKD by 2027. Across the data set, per 100,000 patients and expressed as a weighted mean, *Inside CKD* projected 8859 cases of heart failure, 10,244 of myocardial infarction, and 7797 of stroke per 100,000 patients with CKD by 2027 (Table 4).

**Table 4:** Projected prevalence of cardiovascular complications per 100,000 patients with CKD in 2027.

Region	Country/region	Heart failure	Myocardial infraction	Stroke	Total
Americas	Brazil	7595	3107	2825	13,527
	Canada	9852	6874	9276	26,001
	Colombia	9678	8132	1709	19,519
	Mexico	4947	6703	3962	15,612
	USA	13,114	6505	12,876	32,494
Europe	Belgium	6272	6181	4498	16,951
	Denmark	11,979	10,753	9710	32,442
	France	4666	3662	5923	14,252
	Germany	1307	474	954	2735
	Greece	13,243	11,611	7686	32,540
	Hungary	25,826	5524	9591	40,941
	Italy	13,496	11,758	8641	33,895
	Netherlands	6744	14,469	8059	29,273
	Poland	14,501	17,762	44,838	77,101
	Romania	13,560	15,608	38,915	68,083
	Spain	10,555	4691	4196	19,441
	Sweden	11,569	5508	8011	25,090
	Türkiye	5536	2052	2279	9868
	UK	4707	4267	7827	16,800
Asia–Pacific/Middle East	Australia	20,621	5288	5005	30,914
	China	4987	13,676	9253	27,916
	India	11,259	10,878	5228	27,365
	Israel	3074	2628	2856	8559
	Japan	19,769	21,231	7365	48,366
	Philippines	13,100	13,275	8838	35,213
	Saudi Arabia	6150	3989	1880	12,020
	Singapore	5829	9499	5914	21,243
	South Korea	5795	1995	7251	15,041
	Taiwan	10,759	4687	2949	18,395
	Thailand	6071	16,662	6929	29,661
	UAE Emirati	8017	5816	5068	18,902

CKD = chronic kidney disease.

The UAE comprised the Emirati population and the expatriate population. Only the Emirati populations are considered in the results. The virtual population comprised 20 million individuals, and was scaled to the given country or region population. All CKD stages, cases per 100,000 of the population with CKD.

### Comorbid conditions in the CKD population

The prevalence of comorbidities varied between countries/regions but was generally high in the CKD population in 2027 for hypertension (range: 26,684.8–52,887.3), type 2 diabetes (range: 7072.2–19,445.6) and the co-occurrence of both (range: 9729.8–32,986.4) (Table 5).

**Table 5:** Projected prevalence of comorbid conditions in the population with CKD in 2027.

Region	Country	Hypertension only	Type 2 diabetes only	Hypertension and type 2 diabetes
Americas	Brazil	50,767.4	7195.9	27,527.9
	Canada	38,142.5	13,830.7	18,353.3
	Colombia	52,802.3	7119.0	24,411.4
	Mexico	46,301.8	8265.4	32,986.4
	USA	43,392.3	10,035.9	18,727.9
Europe	Belgium	44,746.4	8832.9	11,001.7
	Denmark	44,809.1	8645.8	10,549.4
	France	42,923.2	9244.0	10,355.8
	Germany	38,994.2	10,179.7	9729.8
	Greece	47,191.4	8073.0	11,057.5
	Italy	51,052.7	7494.9	26,539.5
	Hungary	52,459.1	7411.6	19,982.0
	Netherlands	43,924.0	8984.5	10,525.9
	Poland	45,876.8	8345.0	11,059.2
	Romania	52,878.1	7256.0	18,520.2
	Spain	52,887.3	7072.2	24,257.1
	Sweden	42,048.4	12,333.1	20,661.2
	Türkiye	41,552.4	9716.6	11,018.4
	UK	44,477.8	8880.0	10,901.8
Asia–Pacific/Middle East	Australia	41,937.8	9607.5	10,830.4
	China	38,898.2	10,392.9	10,255.8
	India	45,134.7	9831.3	19,519.1
	Israel	26,684.8	19,445.6	17,865.7
	Japan	44,477.8	8798.2	10,911.5
	Philippines	50,986.2	7227.2	14,694.8
	Saudi Arabia	42,248.3	10,153.2	17,284.0
	Singapore	42,511.7	9452.0	11,037.4
	South Korea	42,676.0	9402.1	11,045.5
	Taiwan	35,880.6	12,793.2	26,631.3
	Thailand	46,358.1	8354.1	11,472.9
	UAE Emirati	42,670.8	9854.0	17,205.7

CKD = chronic kidney disease.

The UAE comprised the Emirati population and the expatriate population. Only the Emirati populations are considered in the results. The virtual population comprised 20 million individuals, and was scaled to the given country or region population. All CKD stages, cases per 100,000 of the population with CKD.

### Projected prevalence of KRT by modality

**Fig. 4:**
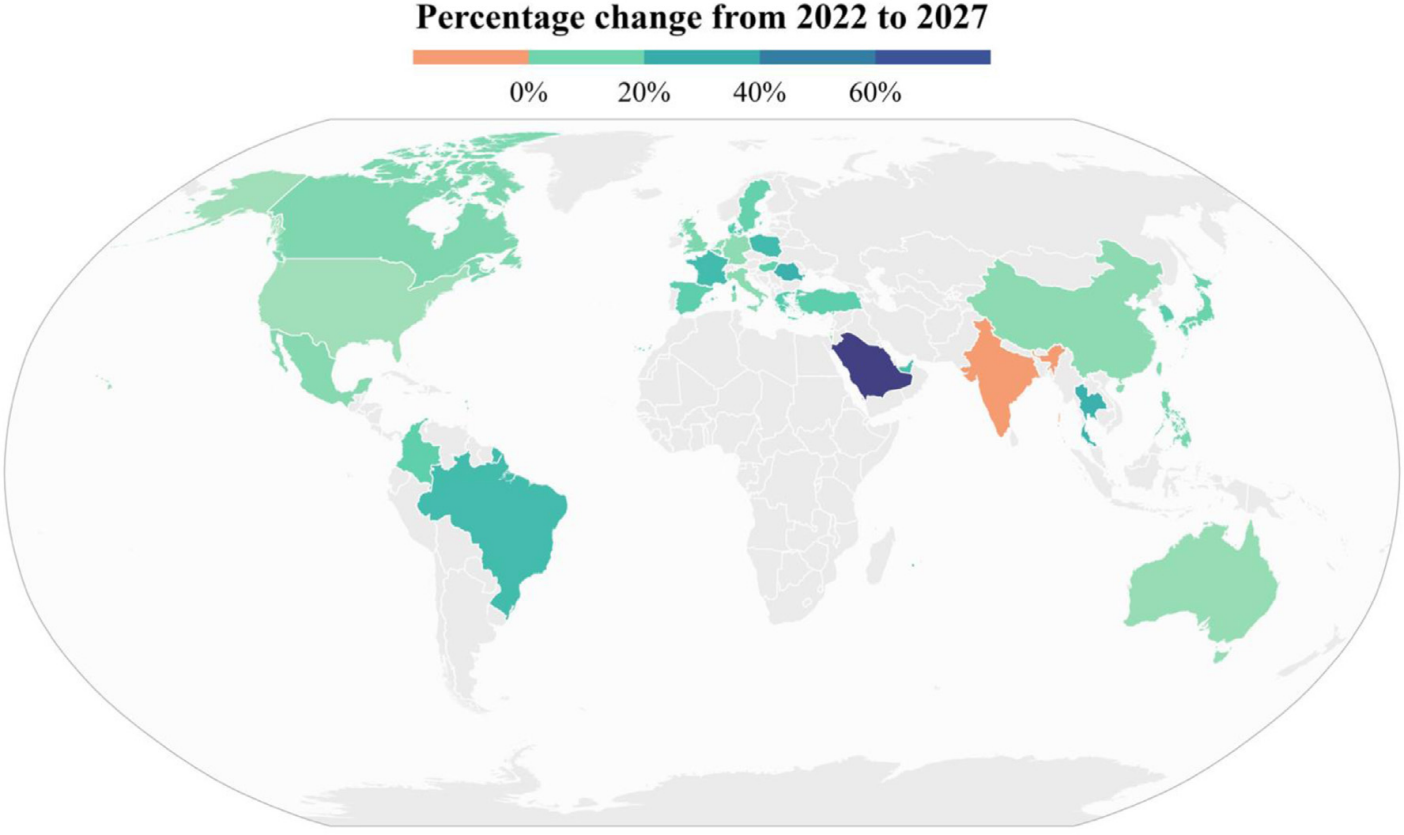
**Projected percentage change in prevalence of kidney replacement therapy from 2022 to 2027.** Notes: The UAE has a large and diverse Expatriate population with a different CKD profile; only the Emirati population has been provided here. CKD = chronic kidney disease.

In diagnosed CKD cases, across the data set the number of patients with moderate to severe kidney disease (stages G3a–G5) was 155 million cases; assuming current treatment strategies remain unchanged over time, the total number of persons receiving KRT is also projected to increase by 7.7% between 2022 and 2027. The use of KRT and its modalities varied considerably across different healthcare systems; across the data set as a whole, haemodialysis was the most common modality at baseline (Supplementary Fig. S2) but trends in use were projected to change slightly with relative increases of 8.5%, 12.5%, and 4.1% for haemodialysis, peritoneal dialysis, and kidney transplantation, respectively between 2022 and 2027 (Fig. 4, Supplementary Table S6).

### All-cause mortality in the diagnosed and undiagnosed CKD population

**Fig. 5:**
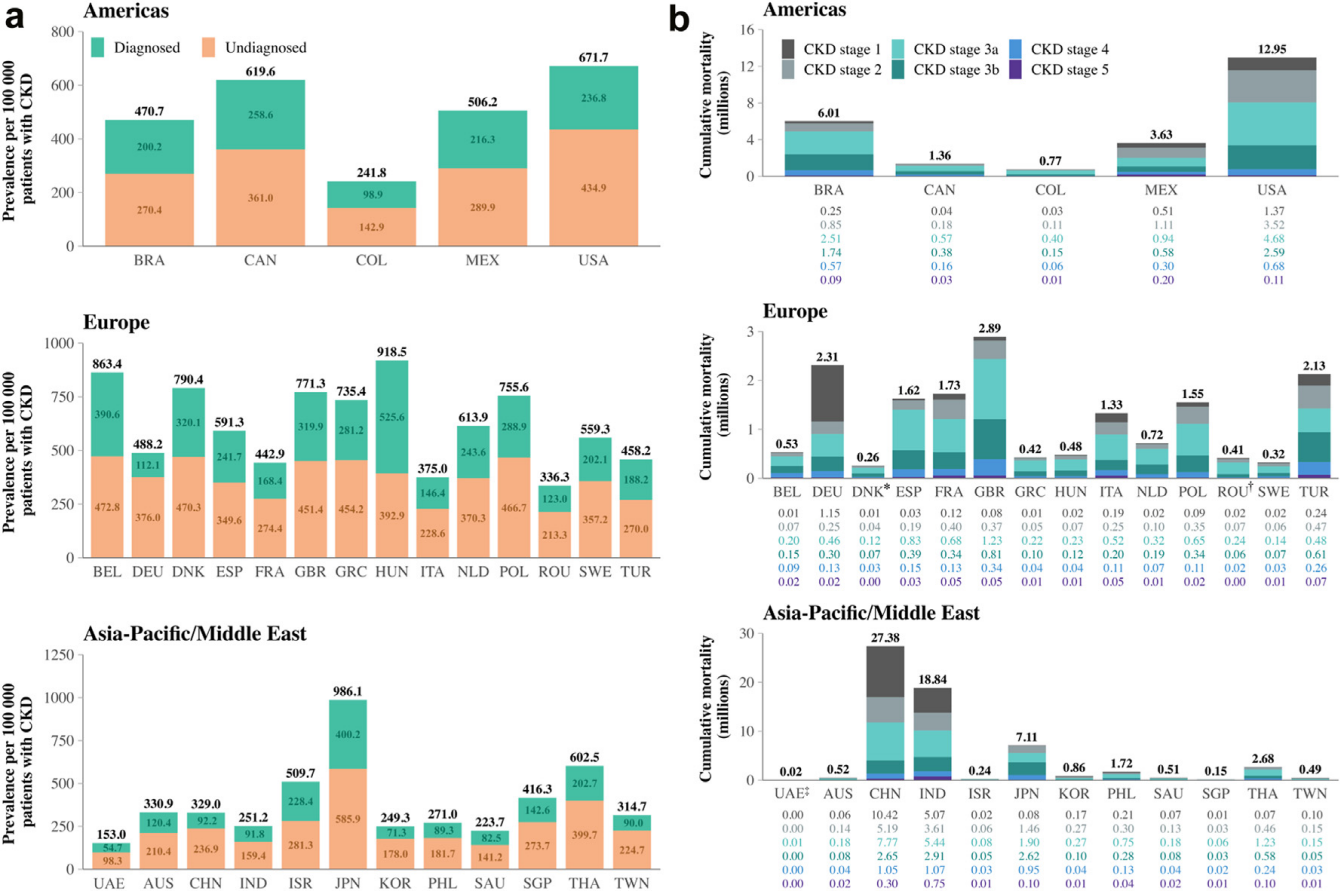
**a: Projected all-cause mortality in the diagnosed and undiagnosed CKD population in 2022, per 100,000 of the population with CKD. b: Projected cumulative incidence of all-cause mortality in people with CKD between 2022 and 2027, by CKD stage.** Notes: The UAE comprised the Emirati population and also a population of those who had migrated to the country, with only the former considered in the results shown. Individual values have been rounded to one–two decimal places. Slight discrepancies may occur between the total sum reported as a result. Cumulative all-cause mortality where values are shown are <0.01 million: ∗Denmark: CKD stage 1, 7970; CKD stage 2, 36,374; CKD stage 3a, 116,474; CKD stage 3b, 69,083; CKD stage 4, 25,190; CKD stage 5, 3820. †Romania: CKD stage 1, 18,261; CKD stage 2, 70,336; CKD stage 3a, 236,637; CKD stage 3b, 61,983; CKD stage 4, 19,474; CKD stage 5, 3041. ‡UAE: CKD stage 1, 2760; CKD stage 2, 4977; CKD stage 3a, 6660; CKD stage 3b, 2427; CKD stage 4, 1439; CKD stage 5, 505.

Our simulation suggests that the effects of CKD are not limited to associated complications and comorbid conditions; in 2022, the prevalence of all-cause mortality in the CKD population varied across our data set, but was generally high (range: 153.0–986.1 cases per 100,000), with deaths in the undiagnosed population generally higher than those in the population diagnosed with CKD (Fig. 5). By 2027, the weighted mean average all-cause mortality rate in the undiagnosed CKD population was 242.5 per 100,000 (range 146.4–545.1), over double that of the diagnosed CKD group 111.4 per 100,000 (range 70.2–391.1).

### Validation analyses and sensitivity analysis

Validation of outputs has been described previously, and a UK example is used to describe the process in detail, which comprised four aspects: external validation, internal validation, cross-validation, and face validity.^16^ This was conducted in accordance with the ISPOR-SMDM task force.^20^ *Inside CKD* outputs were externally validated against literature sources that were not used as data inputs (Supplementary Table S3), which additionally involved consideration of values by the Scientific Steering Committee (face validity). Alongside their expert clinical judgement, the Scientific Steering Committee considered the following factors: (i) the date of the external validation source; (ii) the use of proxy data; and (iii) any assumptions that were applied to *Inside CKD* input data. Combining these factors enabled the Scientific Steering Committee to validate the data set. It is notable that data sources for external validation varied greatly in terms of endpoints, data collection years, and methodology, hence direct comparisons are not provided or advised, but the exercise is included for transparency of reporting.

The sensitivity analysis that was performed on diagnosis rates indicated that the model is robust against minor fluctuations in diagnostic rate (Supplementary Data Appendix 1).

## Discussion

The *Inside CKD* microsimulation is unprecedented in the breadth and granularity of its inputs, as well as the range of countries/regions that it includes. The plethora of factors can be tailored to the epidemiology of a given locality in order to predict future trends. The use of a microsimulation strategy was a core strength of this research because it enabled exploration of a wider remit of outcomes than are usually reported in real-world studies. Microsimulations also provide a future-orientated perspective and allow insight into countries or regions where data are sparse. The model itself can be further adjusted as new data become available, and interventions can be incorporated and tested.^16^ The simulation reported here generated a virtual population of 20 million for 31 countries/regions, each scaled to the corresponding national population. We predicted not only a consistently high prevalence of CKD until 2027, but also a substantial clinical burden, as evidenced by the cumulative incidence of cardiovascular complications, comorbidities, KRT, and all-cause mortality within the CKD population.

In terms of prevalence, our findings are consistent with existing epidemiological data; for example, the CaReMe CKD study used healthcare database analyses from 2020 to estimate a pooled CKD prevalence of 10% in an adult population of 2.4 million across 11 countries.^21^ Our modelled data substantiates the observation that CKD has reached global epidemic levels and predicts that cases are likely to remain high. Of particular concern was the proportion of undiagnosed patients; assuming current diagnostic protocols remain unchanged, most of the CKD population will not be eligible for treatment (unless therapeutic agents are prescribed for alternative reasons, such as angiotensin-converting enzyme inhibitors and angiotensin II receptor blockers for the treatment of hypertension). The implications of clinical and political inertia may be substantial: most deaths in the CKD population are likely to occur among those without a diagnosis. Because people with early-stage CKD are typically asymptomatic, they are unlikely to present at clinic unless seeking support for a different condition.

Diagnosis rates vary in the literature between regions, with current estimates suggesting between 62% and 96% of those with CKD stage 3 are undiagnosed.^22^ Delays in diagnosis can lead to silent disease progression and may accelerate the onset of kidney failure. At this stage, patients usually require KRT, yet the availability of KRT may be limited by costs; the literature suggests access to KRT varies regionally, and 47–73% of patients with kidney failure do not have receive these resource-intensive therapies.^23^^,^^24^ Proactive interventions, such as screening,^25^^,^^26^ earlier diagnosis,^22^ and earlier treatment^27^ have been shown to slow disease progression and to reduce clinical burden, including the need for KRT.^18^, ^19^, ^20^, ^21^, ^22^, ^23^, ^24^ In the absence of these strategies, complications arising from untreated CKD may incur substantial clinical and economic costs.

Investigating across different parameters allows *Inside CKD* to compare how the general trend of increased prevalence is mediated by demographic factors. *Inside CKD* used the World Health Organisation classifications and groupings for age, and found a consistent, direct correlation between prevalence of CKD and the proportion of individuals aged 65 years or older. Advanced age may increase vulnerability to comorbid conditions and eGFR decline,^1^ as well as diminished health-related quality of life.^10^ Future simulations may use age disaggregation to better understand diverse subpopulations of elderly people in different regions. For example, in the Japanese population, which has a relatively high proportion of older people and a long life-expectancy, stratification of individuals aged 65 years or older may help to refine outcome projections.

Type 2 diabetes and hypertension are common among patients with CKD, and can also accelerate CKD progression.^4^^,^^6^^,^^28^ The frequency of both conditions in our data set broadly aligned with age, albeit with some caveats: the highest proportion of type 2 diabetes in the CKD population was projected in Mexico (43.1%), which has a relatively young population. Healthcare interventions focusing on these and other comorbid conditions and their risk factors (e.g. obesity, insulin resistance) may decrease the prevalence and progression of not only CKD but also of related diseases. Exploring cardiovascular complications, we found a high cumulative incidence across the data set, with some variations. Germany had the lowest rate of cardiovascular complications, while unusually high rates were found in Hungary, Poland, Romania, and Japan. The interplay and variability of factors in the *Inside CKD* countries substantiates the need to consider data on a national level and avoid making direct comparisons between countries/regions.

Disease management is mediated by a complex interplay of socioeconomic and demographic factors. For example, gross domestic product and other income categories may influence healthcare systems and the availability of treatments; just as access to KRT was notably varied, so was the modality of approach. Generally, haemodialysis use was proportionately more prevalent in the Asia–Pacific/Middle East region, with a correspondingly lower overall proportion of kidney transplants than in the Americas and Europe. Our findings substantiate the concerns of global advocates, such as the Kidney Health Initiative, which observed that nearly three times as many people will need KRT as will receive it.^29^

In terms of limitations, the eGFR slopes used to determine CKD progression were deemed to be consistent across countries/regions, but this assumption merits some reflection: decline rates or CKD progression may be driven by genetic and or environmental factors. Notably, the literature shows differences for key outcomes (for example, mortality) by ethnicity even after adjustment for possible counfounders.^30^ These differences may indicate a more rapid decline of eGFR due to genetic and/or environmental factors.

The results provided here were contingent on the input data; accordingly, the variation observed among nations' results is attributable not only to distinct demographics (e.g. population size, population ageing, fertility rates) and healthcare infrastructures, but also to disparities in data collection methodologies, temporal differences in input data and nuances in the criteria employed across input sources. This underscores the importance of considering data at a national level and to avoid making direct country comparisons. Gaps and reporting differences in epidemiological data available for input are a common challenge in modelling. For the UAE, United Nations demographic data were utilised for the de facto population, that included Emirati and non-Emirati. A relatively high male:female sex ratio reflects a predominantly male expatriate subgroup within the non-Emirati population. Very little detailed population information is available from the official UAE statistics, and, because data for the whole population (including individuals with and without CKD) were needed, this use of combined statistics for Emirati and non-Emirati was necessary. Our sensitivity analysis suggested that the use of a skewed sex ratio for the Emirati population may not have significantly affected the overall findings, but future research specific to the UAE could explore a greater granularity in the individual Emirate states and their subpopulations (data not shown). It is notable that the UAE has the most rapidly ageing population compared with other countries/regions, which may contribute toward the high projected percentage change of CKD prevalence reported here. The UAE also has one of the smallest national populations that were modelled and outputs such cumulative incidence (i.e. the total sum of incidences that occurred over the analysis period) are directly impacted by population size, and therefore cross-country comparisons should be interpreted carefully. China, as well has having the highest national population, also has a large working age population and a low fertility rate. These unique demographics in addition to national policies aimed at improving health (e.g. Healthy China 2030) may contribute to its relatively low projected percentage change in CKD. Each country/region modelled holds its own unique set of input data, population demographics and country-specific nuances, which warrant careful interpretation.

To the best of our knowledge, we used the most up-to-date and representative epidemiological data for each country or region included in the microsimulation at the time of its first iteration. We acknowledge that new data may have been published in the interim between the first iteration and simulations run for data presented here, and future runs could utilise updated sources. When no suitable data were available for a specific endpoint, a proxy country or region was selected using a project-specific algorithm and informed by an expert panel. Despite our best efforts to ensure comparability, these proxies may not have been fully representative of the population in question. Furthermore, the diverse types of input data used, comprising hospital records, and national statistics, makes it challenging to achieve parity of input definitions for each region. Our literature search highlighted a crucial need for national epidemiological studies of CKD to improve understanding of how discrepancies in healthcare systems may influence patient outcomes. The lack of surveillance that we observed may reflect a low prioritisation in some regions; by promoting awareness of the clinical burden associated with CKD, we hope to encourage improved monitoring and data generation to inform subsequent policy changes. We also acknowledge that periodic updates of literature searches and subsequent microsimulation runs would likely provide useful new information.

Owing to the time of study initiation, the microsimulation did not account for the impact of the COVID-19 pandemic. It is probable that reduced healthcare resources and the interplay of the two diseases affected kidney health, with CKD having influenced COVID-19 disease outcomes and vice versa. The effects of climate change, and the possibility of future pandemics and broad-based geopolitical developments were also not considered; however, the flexibility of dynamic simulation allows future modules to be added that can be tailored to address these or other issues.

In terms of other potential modifications and future iterations, the microsimulation could be extended to quantify the role of other pathologies (e.g., infectious diseases) and the impact of specific interventions on cross-regional health, socioeconomic factors, and health equity. *Inside CKD* modelled medium- and high-income countries using the World Bank definitions, but low-income countries were not modelled; low-income countries may experience higher incidences of CKD drivers because of discrepancies in access to care and vulnerability to environmental factors. These countries could be included in future iterations of the model and, in general, it would be useful to broaden the participation of different nations; notably representation from Africa would be particularly welcome. *Inside CKD* could also be adjusted to investigate how specific at-risk populations are affected by low diagnosis rates. By changing key input parameters, such as the potential impact of screening programmes, we could identify the most appropriate interventions for different patient populations.

Informing healthcare policy may stimulate discussion of protective interventions. Our data strongly suggest that persons with type 2 diabetes, obesity, and/or hypertension represent an ‘at risk’ group who would benefit from initiatives to increase awareness of CKD and, ideally, prevent its development. The cumulative incidence of cardiovascular disease within the CKD population observed supports the benefits of holistic approaches to care across clinical disciplines to minimise the risk of complications. Reviewing demographic risk factors and anticipating changing trends may substantiate national-level decisions for lifestyle interventions, for example, anti-smoking or weight-loss campaigns.

In conclusion, *Inside CKD* projected a global increase in CKD prevalence from 2022 to 2027 that, without intervention, is likely to incur a substantial clinical burden, presenting a clear challenge for global healthcare. Earlier diagnosis combined with timely intervention has the potential to decrease the clinical burden of CKD, protract disease progression, and reduce the use of KRT associated with treating late-stage CKD. In turn, this is likely to lead to a reduced healthcare burden. Our research highlights the need to explore interventions, and ultimately healthcare policy change, to identify people with CKD at an earlier stage of their disease to support timely initiation of treatment aimed at preserving kidney function and preventing complications of CKD.

## Contributors

TC, LW and LR contributed to study conceptualisation, data curation, formal analysis, investigation, methodology, visualisation, data interpretation, writing concept creation at outline, and writing review and editing of all drafts. GMC, RCR, K-U E, EK, AK, GL, CFC, PS, SGH and ECH contributed to study conceptualisation, data curation (review of model inputs and source data), resources, validation, data interpretation, writing concept creation at outline, and writing review and editing of all drafts. JJGS, SB, CC and SN contributed to overall study supervision, study conceptualisation, formal analysis, methodology, visualisation, data interpretation, writing concept creation at outline, and writing review and editing of all drafts. All authors have access to the underlying study data. Authors GC, TC, LW, LR and JJGS verify the underlying study data.

## Data sharing statement

The data sets generated and/or analysed during the current study are available from the corresponding author on reasonable request. In particular, we note that the extensive portfolio of input data may be of interest.

## Declaration of interests

AK has received honoraria for lectures from AstraZeneca. ECH, GL, EK, CFC and RCR have received support from AstraZeneca for their contributions to this work. Stanford University School of Medicine has received grants/contracts from NIDDK, NIAID and CSL Behring on behalf of work conducted by GMC. GMC has received consulting fees from AstraZeneca, Akebia, Ardelyx, Renibus, Miromatrix, Sanifit, Unicycive and Vertex. GMC has received royalties/licences from Elsevier. GMC has participated in advisory boards/data safety monitoring boards by Bayer, Mineralys and ReCor. GMC has a leadership/fiduciary role with Satellite Healthcare Board of Directors (which is non-profit). GMC has stock/stock options at Applaud, CloudCath, Durect, Eliaz Therapeutics, Miromatrix, Outset, Renibus and Unicycive. K-UE has received grants from AstraZeneca, Amgen, Bayer, Evotec and Vifor. K-UE has received consulting fees from Akebia, AstraZeneca, Bayer, Otsuka and Retrophin. K-UE has received honoraria/payment for lectures by AstraZeneca and Bayer. K-UE has participated in advisory boards/data safety monitoring boards by AstraZeneca. LR, LW and TC are employees of HealthLumen Ltd, and AstraZeneca provided funding to HealthLumen Ltd for their contributions to this work. HealThink received funding from AstraZeneca based on the contributions of PS to this work. RCR has received grants/contracts from AstraZeneca. Instituto Nacional de Ciencias Médicas y Nutrición Salvador Zubirán has received grants/contracts from Boehringer Ingelheim, Novo Nordisk, Roche and Baxter on behalf of work conducted by RCR. RCR has received consulting feeds from Chinook, Boehringer, Bayer and Medxl. RCR has received honoraria/payment for lectures by AstraZeneca, Amgen, Boehringer, Novo Nordisk and Bayer. SEHA Kidney Care received honoraria payments from AstraZeneca for the contributions of SGH to this work. JJGS, SB, CC and SN are employees and shareholders of AstraZeneca.
