# Supplementary material for: Projecting the clinical burden of chronic kidney disease at the patient level (Inside CKD): a microsimulation modelling study
Source: eClinicalMedicine. 2024 May 2;72:102614. doi: 10.1016/j.eclinm.2024.102614 (PMC11247147; doi:10.1016/j.eclinm.2024.102614)
Supplement: Supplementary Figs. S1 and S2, Tables S1-S6, and Data Appendix [file mmc1.pdf]

Projecting the clinical burden of chronic kidney disease at the patient level (*Inside CKD*): a microsimulation modelling study

Supplementary material

Authors

Glenn M Chertow, Ricardo Correa-Rotter, Kai-Uwe Eckardt, Eiichiro Kanda, Avraham Karasik, Guisen Li,
Christian Fynbo Christiansen, Panos Stafylas, Stephen G Holt, Ernst C Hagen,
Juan Jose Garcia Sanchez, Salvatore Barone, Claudia Cabrera, Stephen Nolan, Timothy Coker,
Laura Webber, Lise Retat

Contents

Supplementary figures 2
Figure S1a. Prevalence of diagnosed CKD cases per 100 000 of each population, by CKD stage in 2022 2
Figure S1b. Projected prevalence of diagnosed and undiagnosed cases of CKD at baseline (2022) per 100 000 of each population 3
Figure S2. Prevalence of kidney replacement therapy by modality in 2022 4
Supplementary tables 5
Table S1: The Inside CKD Scientific Steering Committee 5
Table S2: Disease burden data inputs 7
Table S3: External validation of clinical burden of disease outputs 38
Table S4: Baseline percentage of drivers of CKD, and country/region income categories in 2022 52
Table S5: Overall prevalence of CKD in 2022 and 2027 expressed as the total number of cases 53
Table S6: KRT prevalence per 100 000 (given for all modalities of treatment) in 2022 and 2027 57
Supplementary data appendix 1: sensitivity analysis on diagnosis rates 59
Supplementary references 75
Table S2 references 75
Table S3 references 83

Supplementary figures

Figure S1a. Prevalence of diagnosed CKD cases per 100 000 of each population, by CKD stage in 2022

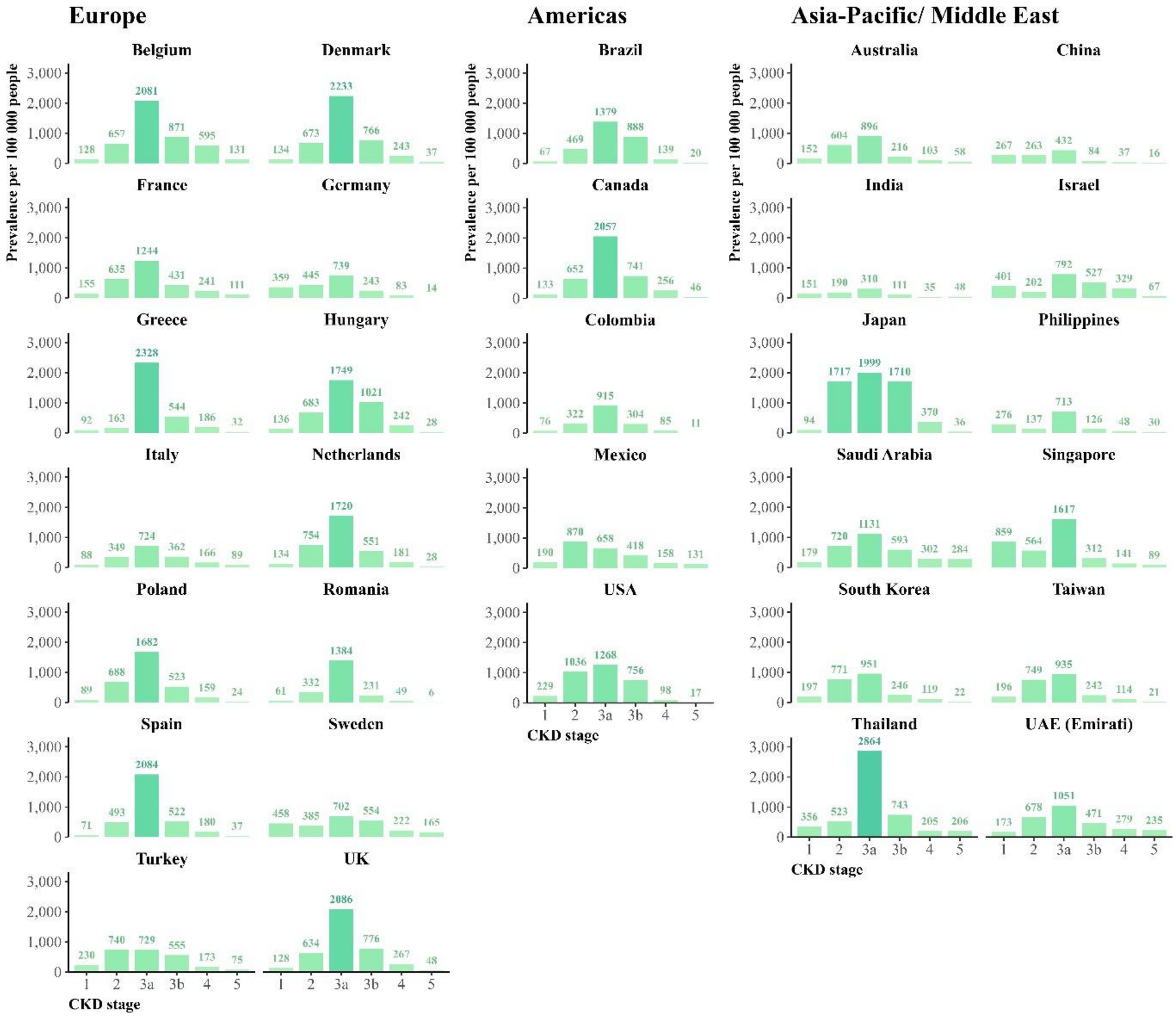

Figure S1b. Projected prevalence of diagnosed and undiagnosed cases of CKD at baseline (2022) per 100 000 of each population

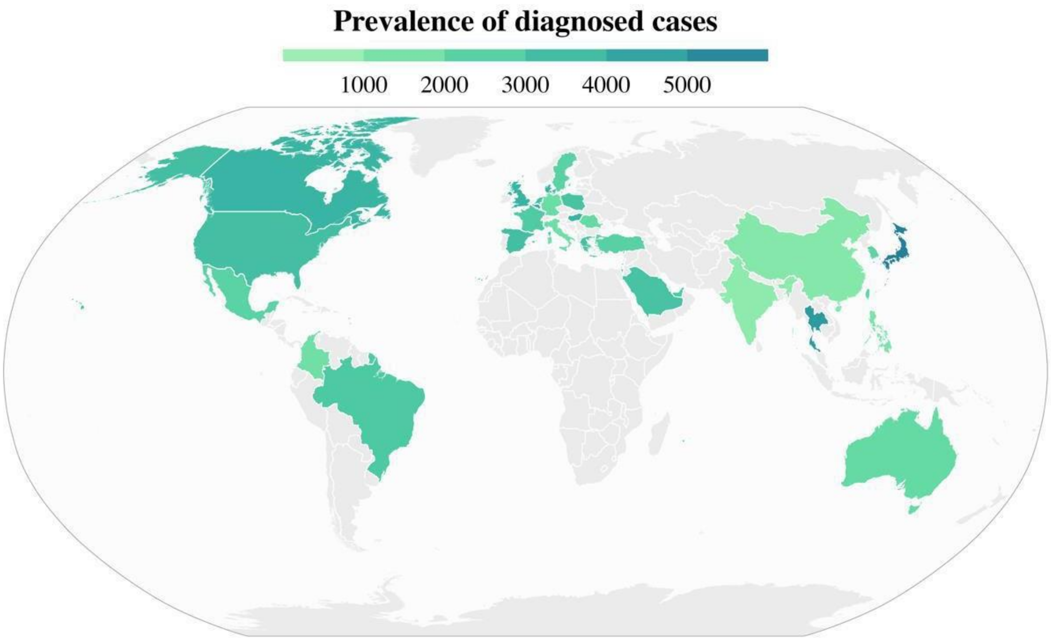

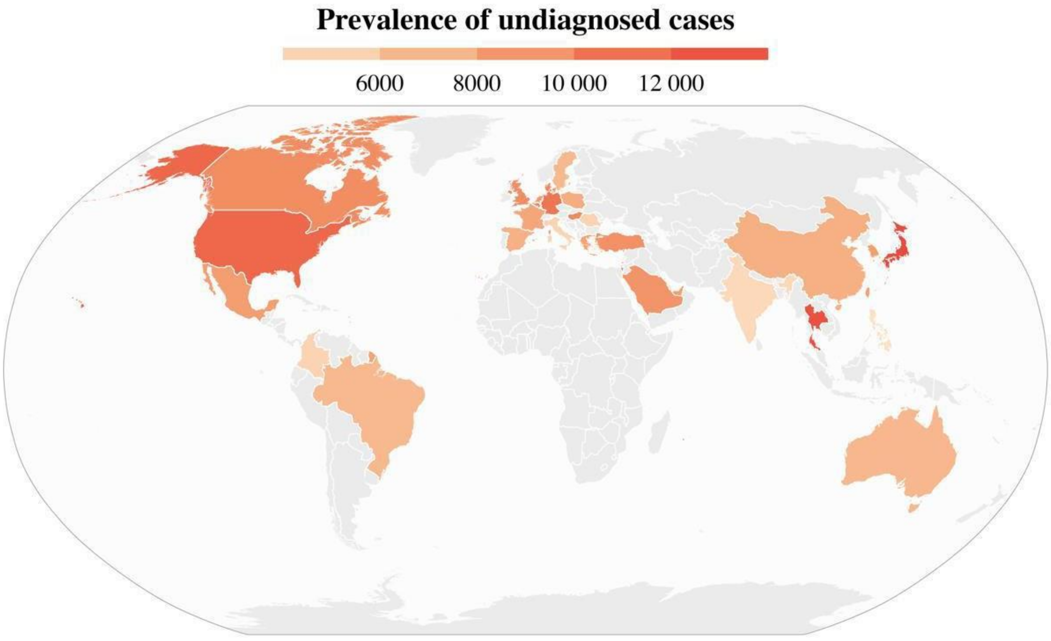

CKD=chronic kidney disease. Notes: The UAE has a large and diverse Expatriate population with a different CKD profile; only the Emirati population has been provided here.

Figure S2. Prevalence of kidney replacement therapy by modality in 2022

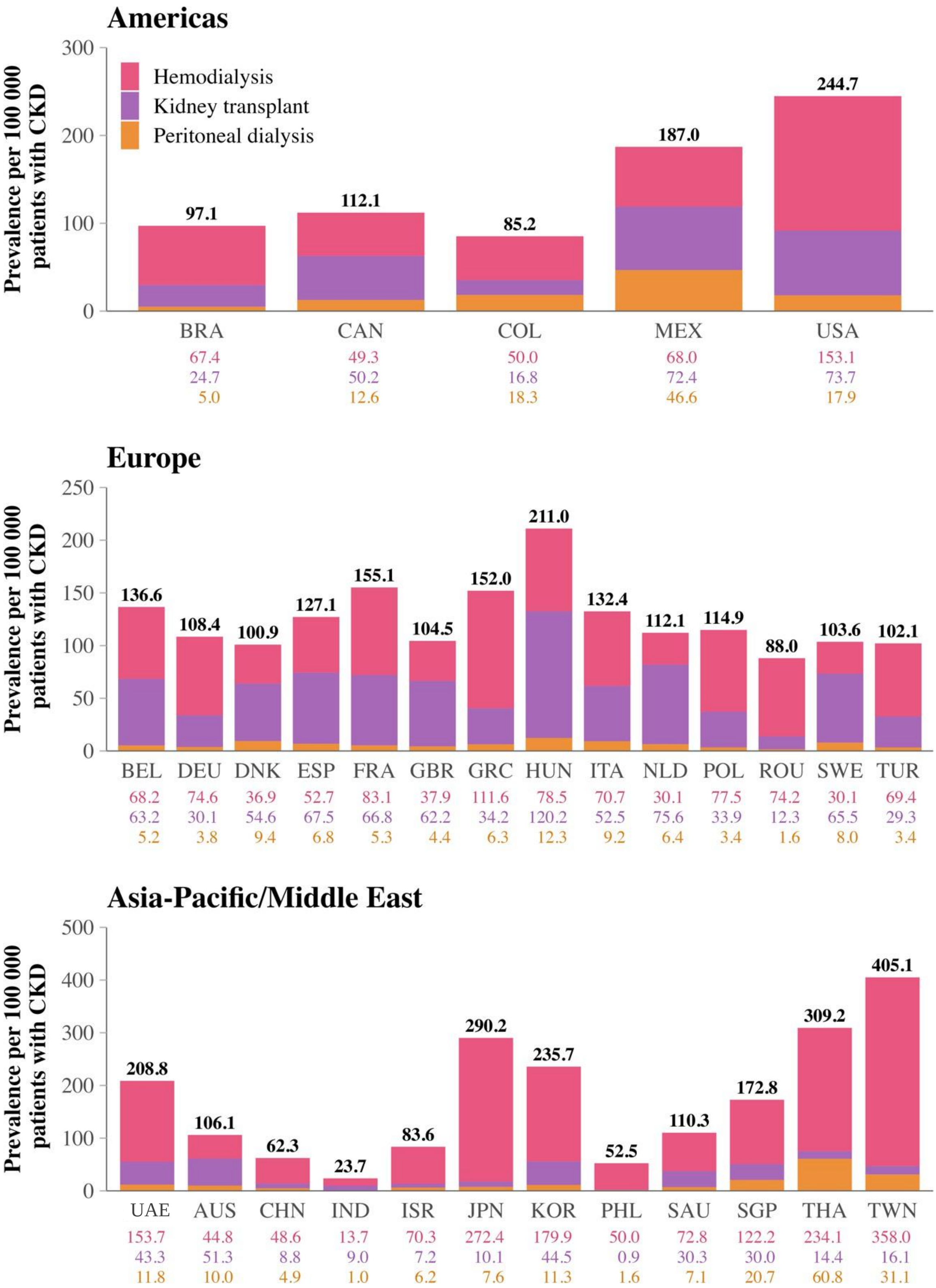

Notes: The UAE has a large and diverse Expatriate population with a different CKD profile; only the Emirati population has been shown here. Individual values have been rounded to one decimal place. Slight discrepancies may occur between the total sum reported as a result. CKD=chronic kidney disease.

Supplementary tables

Table S1: The Inside CKD Scientific Steering Committee

Region	Key external expert	Affiliation
Australia	Prof. Steven Chadban	Royal Prince Alfred Hospital and University of Sydney, Camperdown, Australia
Belgium	Prof. Michel Jadoul	Cliniques Universitaires Saint-Luc, Université catholique de Louvain, Brussels, Belgium
Brazil	Prof. Marcelo Costa Batista	Hospital Israelita Albert Einstein, São Paulo, Brazil
Canada	Dr Navdeep Tangri	University of Manitoba, Winnipeg, Canada
China	Prof. Guisen Li	Sichuan Academy of Medical Sciences and Sichuan Provincial People's Hospital, Chengdu, China
Colombia	Prof. José Javier Arango Álvarez	Universidad del Quindío, Quindío, Colombia
Denmark	Prof. Christian Fynbo Christiansen	Aarhus University and Aarhus University Hospital, Aarhus, Denmark
France	Prof. Jean-Michel Halimi	Service de néphrologie-HTA, dialyses et transplantation rénale, CHRU de Tours, Tours, France
Germany	Prof. Kai-Uwe Eckardt	Charité Universitätsmedizin Berlin, Berlin, Germany
Greece	Dr Panos Stafylas	HealThink, Thessaloniki, Greece
Hungary	Prof. István Wittmann, Dr Boglarka Laczy	University of Pécs, Pécs, Hungary
India	Prof. Vivekanand Jha	The George Institute for Global Health India, New Delhi, India
Israel	Prof. Avraham Karasik	Maccabi Institute for Research and Innovation, Tel Aviv, Israel
Israel	Dr Gil Chernin	Kaplan Medical Center, Faculty of Medicine, Hebrew University of Jerusalem, Jerusalem, Israel
Italy	Prof. Francesco Saverio Mennini	Economic Evaluation and HTA-CEIS, Department of Economics and Finance, Faculty of Economics, University of Rome "Tor Vergata", Rome, Italy
Italy	Prof. Luca De Nicola	Department of Advanced Medical and Surgical Sciences, University of Campania Luigi Vanvitelli, Naples, Italy
Japan	Prof. Eiichiro Kanda	Kawasaki Medical School, Okayama, Japan
Mexico	Prof. Ricardo Correa-Rotter	Instituto Nacional de Ciencias Médicas y Nutrición Salvador Zubirán<br>Mexico City, Mexico
Netherlands	Dr Ernst C Hagen	Meander Medical Centre, Amersfoort, Netherlands
Philippines, Singapore, and Thailand	Assoc. Prof. Jason Choo Chon Jun	Singapore General Hospital, Singapore
Poland	Prof. Michał Nowicki	Warsaw University of Technology, Warsaw, Poland
Romania	Prof. Dr Ismail Gener	Fundeni Institute, Bucharest, Romania

Saudi Arabia	Prof. Saeed M. G. Al-Ghamdi	King Abdulaziz University Hospital and King Faisal Specialist Hospital and Research Centre, Jeddah, Saudi Arabia
South Korea	Prof. Kook-Hwan Oh	Seoul National University College of Medicine, Seoul, South Korea
Spain	Prof. Juan Francisco Navarro-González	Research Unit and Nephrology Service, University Hospital Nuestra Señora de Candelaria, Santa Cruz de Tenerife, Spain
Sweden	Prof. Johan Ärnlöv	Department of Neurobiology, Care Sciences and Society, Division of Family Medicine and Primary Care, Karolinska Institute, Stockholm, Sweden
Taiwan	Prof. Mai-Szu Wu	Department of Internal Medicine, School of Medicine, College of Medicine, Taipei Medical University, Taipei City, Taiwan<br><br>Taipei Medical University Research Center of Urology and Kidney, Taipei City, Taiwan<br><br>Taipei Medical University Shuang Ho Hospital, New Taipei City, Taiwan
Türkiye	Prof. Mustafa Arıcı	Division of Nephrology, Department of Internal Medicine, Hacettepe University, Ankara, Türkiye
UAE	Prof. Stephen G Holt	SEHA Kidney Care, Abu Dhabi, UAE
UK	Dr Albert Power	North Bristol NHS Trust, Southmead Hospital, Bristol, UK
USA	Prof. Glenn Chertow	Stanford University School of Medicine, Stanford, CA, USA
USA	Prof. Jay Wish	Indiana University School of Medicine, Indianapolis, IN, USA

CEIS=Centre for Economic and International Studies. CHRU=Centre Hospitalier Régional Universitaire. CKD=chronic kidney disease. HTA=health technology assessment. NHS=National Health Service. SEHA=Abu Dhabi Health Services Company.

Table S2: Disease burden data inputs

Parameter	Reference	Proxy country	Definitions
**Australia**			
eGFR	Australian Health Survey 2011–12 [1]	-	Distribution of eGFR and albuminuria in individuals aged ≥18 (cohort of 17 042) with each stage of CKD
Albuminuria			
**KRT parameters**			
KRT initiation threshold	Prof. Steven Chadban, personal communication	-	
Chance of being treated	Sparke et al., 2013 [2]	-	
Peritoneal dialysis	ANZDATA 43rd Annual Report, 2020 [3]	-	Incidence and prevalence of patients receiving KRT who are undergoing peritoneal dialysis and haemodialysis
Haemodialysis			Prevalence of KRT by year
Transplantation			Transplantation failure per year
**Comorbidities of CKD**			
Type 2 diabetes	HSE, 2016 [4]	UK	Categorized by albuminuria level
Hypertension			
**Cardiovascular complications of CKD**			
Heart failure	British Heart Foundation, 2020 [5]	UK	Incidence
	Calculated from Barendregt et al., 2003 [6]	-	Prevalence
	USRDS, 2009 [7]	USA	Relative risk
Myocardial infarction	Australian Bureau of Statistics, 2018 [8]	-	Prevalence (incidence calculated)
	USRDS, 2009 [7]	USA	Relative risk
Stroke	Stroke Foundation and Deloitte Access Economics, 2020 [9]	-	Incidence and prevalence
	Masson et al., 2015 [10]	-	Relative risk
**Mortality**			
Mortality by CKD stage	UN, World Population Prospects, 2019 [11, 12]	-	Total number of deaths by age and sex
	Levey et al., 2011 [13]	-	Probability of dying from any cause by CKD stage
**Diagnosis rates**			
Diagnosis rates by stage	Hirst et al., 2020 [14]	UK	Average calculated from both inputs
	HSE, 2016 [4]	UK	
**Belgium**			
eGFR	HSE, 2016 [4]	UK	eGFR by age category, disaggregated based on [4]
	Van Pottelbergh et al., 2012 [15]	-	

Albuminuria	HSE, 2016 [4]	UK	AER by age category
**KRT parameters**			
KRT initiation threshold	Prof. Michel Jadoul, personal communication	-	
Chance of being treated	O'Callaghan et al., 2011 [16]	UK	
Peritoneal dialysis	Cleemput et al., 2010 [17]	-	Patients undergoing peritoneal dialysis or haemodialysis
Haemodialysis	Jaarrapport, 2019 [18]	-	Prevalence of KRT by year, and transplantation rate per dialysis patient-year
Transplantation	UK Renal Registry (p91) [19]	UK	Risk of transplantation failure
**Comorbidities of CKD**			
Type 2 diabetes	HSE, 2016 [4]	UK	Categorized by albuminuria level
Hypertension	HSE, 2016 [4]	UK	
	Barzilay et al., 2018 [20]	UK	
**Cardiovascular complications of CKD**			
Heart failure	Devroey et al., 2010 [21]	-	Incidence
	Smeets et al., 2019 [22]	-	Prevalence
	USRDS, 2009 [7]	USA	Relative risk
Myocardial infarction	Belgian Federal Public Service: Health, Food Chain Safety and Environment, 2016 [23]	-	Incidence
	Moran et al., 2012 [24]	-	Prevalence
	USRDS, 2009 [7]	USA	Relative risk
Stroke	IHME Global Burden of Diseases, 2019 [25]	-	Incidence and prevalence
	Masson et al., 2015 [10]	-	Relative risk
**Mortality**			
Mortality by CKD stage	UN, World Population Prospects, 2019 [11, 12]	-	Total number of deaths by age and sex
	Levey et al., 2011 [13]	-	Probability of dying from any cause by CKD stage
**Diagnosis rates**			
Diagnosis rates by stage	Hirst et al., 2020 [14]	UK	Average calculated from both inputs
	HSE, 2016 [4]	UK	
**Brazil**			
eGFR	Piccolli et al., 2017 [26]	-	Categorical eGFR data for both sexes combined, for three age groups (18–29 years, 30–59 years, and ≥60 years). Redistributed based on [4]

Albuminuria	Piccolli et al., 2017 [26]	-	Categorical albuminuria data by sex and albuminuria stage combined
**KRT parameters**			
KRT initiation threshold	Pyart et al., 2020 [27]	UK	
Chance of being treated	Organización Nacional de Trasplantes, 2018 [28]	Spain	
Peritoneal dialysis	Machado et al., 2011 [29]	-	Incident peritoneal dialysis or haemodialysis
	Brazilian Dialysis Registry [30] (not publicly accessible)	-	Prevalent peritoneal dialysis or haemodialysis
Haemodialysis	Organización Nacional de Trasplantes, 2018 [28]	-	Prevalence of KRT by modality
	Neves et al., 2021 [31]	Spain	Calculated transplantation rate per 100 dialysis patients per year
Transplantation	Oliveira et al., 2005 [32]	-	Risk of transplantation failure
**Comorbidities of CKD**			
Type 2 diabetes	Ruiz-Hurtado et al., 2016 [33]	Spain	Categorized by albuminuria level
Hypertension			
**Cardiovascular complications of CKD**			
Heart failure	Fernández-Gassó et al., 2019 [34]	Spain	Incidence
	Oliveira et al., 2020 [35]	-	Prevalence
	USRDS, 2009 [7]	USA	Relative risk
Myocardial infarction	Forcadell et al., 2018 [36]	Spain	Incidence (prevalence calculated)
	USRDS, 2009 [7]	USA	Relative risk
Stroke	Vega et al., 2009 [37]	Spain	Incidence (prevalence calculated)
	Masson et al., 2015 [10]	-	Relative risk
**Mortality**			
Mortality by CKD stage	UN, World Population Prospects, 2019 [11, 12]	-	Total number of deaths by age and sex
	Levey et al., 2011 [13]	-	Probability of dying from any cause by CKD stage
**Diagnosis rates**			
Diagnosis rates by stage	Hirst et al., 2020 [14]	UK	Stages 1 and 2
	Average from TriNetX and LCED database analysis by AstraZeneca (data on file)	USA	Stages 3a and 3b
	Bakris et al., 2020 [38]	USA	Stages 4 and 5

**Canada**			
eGFR	HSE, 2016 [4]	UK	Percentage distribution of eGFR and albuminuria by age/sex
Albuminuria			
**KRT parameters**			
KRT initiation threshold	Dr Navdeep Tangri, personal communication	-	
Chance of being treated	O'Callaghan et al., 2011 [16]	UK	
Peritoneal dialysis	Canadian Institute for Health Information, 2018 [39]	-	Renal replacement therapy model in Canada
Haemodialysis	USRDS, 2021 [40]	-	Transplantation rate per dialysis patient-year
Transplantation	Pyart et al., 2020 [27]	-	Risk of transplantation failure
**Comorbidities of CKD**			
Type 2 diabetes	Armstrong et al., 2019 [41]	-	Proportion of patients with CKD, categorized by albuminuria level
Hypertension	NHANES, 2018 [42]	-	
**Cardiovascular complications of CKD**			
Heart failure	Canadian Chronic Disease Surveillance System, 2016 [43]	-	Incidence and prevalence
	USRDS, 2009 [7]	USA	Relative risk
Myocardial infarction	Canadian Chronic Disease Surveillance System, 2016 [43]	-	Incidence and prevalence
	USRDS, 2009 [7]	USA	Relative risk
Stroke	Canadian Chronic Disease Surveillance System, 2016 [43]	-	Incidence and prevalence
	Masson et al., 2015 [10]	USA	Relative risk
**Mortality**			
Mortality by CKD stage	UN, World Population Prospects, 2019 [11, 12]	-	
	Levey et al., 2011 [13]	-	Probability of dying from any cause by CKD stage
**Diagnosis rates**			
Diagnosis rates by stage	Hirst et al., 2020 [14]	UK	Average calculated from both inputs
	HSE, 2016 [4]	UK	
**China**			
eGFR	Zhang et al., 2012 [44]	-	Categorical eGFR data for adults, distributed by age/sex (disaggregated based on [4])
Albuminuria	Japanese Society of Nephrology, 2012 [45]	Japan	Categorical albuminuria data for both sexes, by age (disaggregated based on [4])
**KRT parameters**			
KRT initiation threshold	Watanabe et al., 2015 [46]	Japan	

Chance of being treated	Zhang et al., 2019 [47]	-	
Peritoneal dialysis	Zhang et al., 2019 [47]	-	Prevalence of KRT by year
	Chinese National Renal Data System [48]	-	Incidence of peritoneal dialysis and haemodialysis
Haemodialysis	Sun et al., 2016 [49]	-	Prevalence of peritoneal dialysis and haemodialysis
Transplantation	Chinese National Renal Data System [48]	-	Transplantation rate per dialysis patient-year
	Xue et al., 2017 [50]	-	Risk of transplantation failure
**Comorbidities of CKD**			
Type 2 diabetes	HSE, 2016 [4]	UK	Prevalence of type 2 diabetes and hypertension in the CKD population, by albuminuria level
Hypertension	Barzilay et al., 2018 [20]	UK	
	HSE, 2016 [4]	UK	
**Cardiovascular complications of CKD**			
Heart failure	Sun et al., 2009 [51]	-	Prevalence (incidence calculated)
	USRDS, 2009 [7]	USA	Relative risk
Myocardial infarction	Japanese-language paper [52]	Japan	Incidence (prevalence calculated)
	USRDS, 2009 [7]	USA	Relative risk
Stroke	IHME, Global Burden of Diseases, 2019 [25]	-	Prevalence and incidence
	Masson et al., 2015 [10]	-	Relative risk
**Mortality**			
Mortality by CKD stage	UN, World Population Prospects, 2019 [11, 12]	-	Total number of deaths by age and sex
	Levey et al., 2011 [13]		Probability of dying from any cause by CKD stage
**Diagnosis rates**			
Diagnosis rates by stage	Hirst et al., 2020 [14]	UK	Average calculated from both inputs
	HSE, 2016 [4]	UK	
**Colombia**			
eGFR	Otero et al., 2010 [53]	Spain	eGFR stages redistributed by UK data
Albuminuria	Gorostidi et al., 2018 [54]	Spain	
**KRT parameters**			
KRT initiation threshold	Dr Albert Power, personal communication	UK	
Chance of being treated	O'Callaghan et al., 2011 [16]	UK	
Peritoneal dialysis	Calculated from Cuenta de Alto Costo, 2021 [55]	-	Incidence and prevalence of patients receiving KRT who are undergoing peritoneal dialysis and haemodialysis

Haemodialysis	Cuenta de Alto Costo, 2021 [55]<br>Urrego et al., 2022 [56]	-<br>-	Prevalence of KRT by year<br><br>Transplantation number per year
Transplantation	Estupiñán-Bohórquez et al., 2021 [57]	-	Annual risk of transplantation failure
**Comorbidities of CKD**			
Type 2 diabetes	Ruiz-Hurtado et al., 2016 [33]	Spain	Categorized by albuminuria level
Hypertension			
**Cardiovascular complications of CKD**			
Heart failure	Gómez, 2016 [58	-	Incidence<br><br>Distribution by age and sex
	SISPRO [59]	-	
	Gómez-Mesa et al., 2021 [60]	-	Prevalence<br><br>Distribution by age and sex
	SISPRO [59]	-	
	USRDS, 2009 [7]	USA	Relative risk
Myocardial infarction	Canadian Chronic Disease Surveillance System, 2016 [43]	Canada	Incidence
	Bedoya-Rios et al., 2016 [61]	-	Prevalence<br><br>Distribution by age and sex
	SISPRO [59]	-	
	USRDS, 2009 [7]	USA	Relative risk
Stroke	SISPRO and Congreso de la República Colombiana, 2021 [59]	-	Incidence
	El Hospital, 2018 [62]	-	Prevalence<br><br>Distribution by age and sex
	SISPRO [58]	-	
	Masson et al., 2015 [10]	-	Relative risk
**Mortality**			
Mortality by CKD stage	UN, World Population Prospects, 2019 [11, 12]	-	Total number of deaths by age and sex
	Levey et al., 2011 [13]	-	Probability of dying from any cause by CKD stage
**Diagnosis rates**			
Diagnosis rates by stage	Hirst et al., 2020 [14]	UK	Stages 1 and 2
	Average from TriNetX and LCED database analysis by AstraZeneca (data on file)	USA	Stages 3a and 3b
	Bakris et al., 2020 [38]	USA	Stages 4 and 5
**Denmark**			
eGFR	HSE, 2016 [4]	UK	eGFR and AER by age
Albuminuria			
**KRT parameters**			
KRT initiation threshold	Danish Society of Nephrology, 2015 [63]	-	

Chance of being treated	Danish Nephrological Society National Register, 2020 [64]	-	
Peritoneal dialysis	Danish Nephrological Society National Register, 2020 [64]	-	Incidence and prevalence of patients receiving KRT who are undergoing peritoneal dialysis and haemodialysis
Haemodialysis			Prevalence of KRT by year<br>Transplantation rate per 100 dialysis patients per year
Transplantation	Sundhend, 2019 [65]	-	Annual risk of transplantation failure
**Comorbidities of CKD**			
Type 2 diabetes	HSE, 2016 [4]	UK	Prevalence of type 2 diabetes and hypertension in the CKD population, by albuminuria level
Hypertension	Barzilay et al., 2018 [20]	UK	
	HSE, 2016 [4]	UK	
**Cardiovascular complications of CKD**			
Heart failure	Hjerte foreningen, 2018 [66]	-	Incidence
	Hjerte foreningen, 2018 [67]	-	Prevalence
	USRDS, 2009 [7]	USA	Relative risk
Myocardial infarction	Hjerte foreningen, 2018 [68]	-	Incidence
	Hjerte foreningen, 2018 [67]	-	Prevalence
	USRDS, 2009 [7]	USA	Relative risk
Stroke	Hjerte foreningen, 2018 [69]	-	Incidence
	Hjerte foreningen, 2018 [70]	-	Prevalence
	Masson et al., 2015 [10]	-	Relative risk
**Mortality**			
Mortality by CKD stage	UN, World Population Prospects, 2019 [11, 12]	-	Total number of deaths by age and sex
	Levey et al., 2011 [13]	-	Probability of dying from any cause by CKD stage
**Diagnosis rates**			
Diagnosis rates by stage	Hirst et al., 2020 [14]	UK	Average calculated from both inputs
	HSE, 2016 [4]	UK	
**France**			
eGFR	HSE, 2016 [4]	UK	eGFR stages by 5-year age categories
Albuminuria	HSE, 2016 [4]	UK	AER stages by 5-year age categories
**KRT parameters**			
KRT initiation threshold	Pyart et al., 2020 [27]	UK	
Chance of being treated	REIN report, 2018 [71]	-	

Peritoneal dialysis<br>Haemodialysis<br>Transplantation	REIN report, 2018 [71]	-	Incidence and prevalence of patients receiving KRT who are undergoing peritoneal dialysis and haemodialysis<br>Prevalence of KRT by year<br>Transplantation rate per 100 dialysis patients per year<br>Annual risk of transplantation failure
**Comorbidities of CKD**			
Type 2 diabetes<br>Hypertension	HSE, 2016 [4]	UK	Prevalence of type 2 diabetes and hypertension in the CKD population, by albuminuria level
**Cardiovascular complications of CKD**			
Heart failure	Analysis of THIN, 2019 (data on file)	-	Incidence and prevalence
	USRDS, 2009 [7]	USA	Relative risk
Myocardial infarction	Analysis of THIN, 2019 (data on file)	-	Incidence and prevalence
	USRDS, 2009 [7]	USA	Relative risk
Stroke	Analysis of THIN, 2019 (data on file)	-	Incidence and prevalence
	Masson et al., 2015 [10]	-	Relative risk
**Mortality**			
Mortality by CKD stage	UN, World Population Prospects, 2019 [11, 12]	-	Total number of deaths by age and sex
	Levey et al., 2011 [13]	-	Probability of dying from any cause by CKD stage
**Diagnosis rates**			
Diagnosis rates by stage	Hirst et al., 2020 [14]	UK	Average calculated from both inputs
	HSE, 2016 [4]	UK	
**Germany**			
eGFR<br>Albuminuria	Girndt et al., 2016 [72]	-	eGFR by age category, disaggregated based on [5]
**KRT parameters**			
KRT initiation threshold	Wanner et al., 2003 [73]	-	
Chance of being treated	Frei et al., 2008 [74]	-	
Peritoneal dialysis<br>Haemodialysis	Frei et al., 2008 [74]	-	Incidence and prevalence of patients receiving KRT who were undergoing peritoneal dialysis and haemodialysis
Transplantation	Satayathum et al., 2005 [75]	-	Rate per 100 dialysis patients per year

	Frei et al., 2008 [74]	-	Risk of transplantation failure
**Comorbidities of CKD**			
Type 2 diabetes	HSE, 2016 [4]	UK	Prevalence of type 2 diabetes and hypertension in the CKD population, by albuminuria level
Hypertension	Barzilay et al., 2018 [20]	UK	
	HSE, 2016 [4]	UK	
**Cardiovascular complications of CKD**			
Heart failure	GBE Federation, 2021 [76]	-	Incidence calculated from prevalence
	USRDS, 2009 [7]	USA	Relative risk
Myocardial infarction	GBE Federation, 2021 [76]	-	Incidence calculated from prevalence
	USRDS, 2009 [7]	USA	Relative risk
Stroke	GBE Federation, 2021 [76]	-	Incidence calculated from prevalence
	Masson et al., 2015 [10]	-	Relative risk
**Mortality**			
Mortality by CKD stage	UN, World Population Prospects, 2019 [11, 12]	-	Total number of deaths by age and sex
	Levey et al., 2011 [13]	-	Probability of dying from any cause by CKD stage
**Diagnosis rates**			
Diagnosis rates by stage	Hirst et al., 2020 [14]	UK	Average calculated from both inputs
	HSE, 2016 [4]	UK	
**Greece**			
eGFR	Otero et al., 2010 [53]	Spain	eGFR by age category, redistributed based on UK data
Albuminuria	Gorostidi et al., 2018 [54]	Spain	UACR by age category, redistributed based on UK data
**KRT parameters**			
KRT initiation threshold	Pyart et al., 2020 [27]	UK	
Chance of being treated	ERA-EDTA Registry, 2019 [77]	-	
Peritoneal dialysis	ERA-EDTA Registry, 2019 [77]	-	Incidence and prevalence of patients receiving KRT who are undergoing peritoneal dialysis and haemodialysis<br><br>Prevalence of KRT by year<br><br>Transplantation rate per 100 dialysis patient-years
Haemodialysis			

Transplantation	Grandaliano, 2020 [78]	Italy	Annual risk of transplantation failure (calculated from 10-year failure rate of 40%)
**Comorbidities of CKD**			
Type 2 diabetes	HSE, 2016 [4]	UK	Prevalence of type 2 diabetes and hypertension in the CKD population, by albuminuria level
Hypertension	Barzilay et al., 2018 [20]	UK	
	HSE, 2016 [4]	UK	
**Cardiovascular complications of CKD**			
Heart failure	Piccinni et al., 2017 [79]	Italy	Incidence
	Bosch et al., 2019 [80]	-	Prevalence
	USRDS, 2009 [7]	USA	Relative risk
Myocardial infarction	Registro dell'infarto miocardico acuto nell'ASL di Brescia [81]	Italy	Incidence
	Hellenic Statistical Authority, 2020 [82]	-	Prevalence
	USRDS, 2009 [7]	USA	Relative risk
Stroke	Karantali et al., 2022 [83]	-	Incidence
	Hellenic Statistical Authority, 2020 [82]	-	Prevalence
	Masson et al., 2015 [10]	-	Relative risk
**Mortality**			
Mortality by CKD stage	UN, World Population Prospects, 2019 [11, 12]	-	Total number of deaths by age and sex
	Levey et al., 2011 [13]	-	Probability of dying from any cause by CKD stage
**Diagnosis rates**			
Diagnosis rates by stage	Hirst et al., 2020 [14]	UK	Average calculated from both inputs
	HSE, 2016 [4]	UK	
	Maccabi database (data on file)	Israel	Stages 1 and 2
**Hungary**			
eGFR	Zdrojewski et al., 2015 [84]	Poland	eGFR data by age and two risk categories, disaggregated using UK data
Albuminuria	Chudek et al., 2014 [85]	Poland	Albuminuria data by age and risk category
**KRT parameters**			
KRT initiation threshold	Pyart et al., 2020 [27]	UK	
Chance of being treated	O'Callaghan et al., 2011 [16]	UK	

Peritoneal dialysis	Calculated using NHIFA real-world data	-	Incidence and prevalence of patients receiving KRT who are undergoing peritoneal dialysis and haemodialysis
Haemodialysis	MANET - Hungarian Society of Nephrology 2016–2019 [86]	-	Prevalence of KRT per 100 000 by year
	NHIFA real-world data (data on file)	-	Transplantation rate per 100 dialysis patient-years
Transplantation	Analysis from national inpatient claims database (data on file)	Romania	Annual risk of transplantation failure
**Comorbidities of CKD**			
Type 2 diabetes	Analysis from national inpatient claims database (data on file)	Romania	Proportion of type 2 diabetes and hypertension in the CKD population, by albuminuria level
Hypertension			
**Cardiovascular complications of CKD**			
Heart failure	Hungarian NHIFA analysis, 2019 (data on file)	-	Incidence and prevalence
	USRDS, 2009 [7]	USA	Relative risk
Myocardial infarction	Hungarian NHIFA analysis, 2019 (data on file)	-	Incidence and prevalence
	USRDS, 2009 [7]	USA	Relative risk
Stroke	Hungarian NHIFA analysis, 2019 (data on file)	-	Incidence and prevalence
	Masson et al., 2015 [10]	-	Relative risk
**Mortality**			
Mortality by CKD stage	UN, World Population Prospects, 2019 [11, 12]	-	Total number of deaths by age and sex
	Levey et al., 2011 [13]	-	Probability of dying from any cause by CKD stage
**Diagnosis rates**			
Diagnosis rates by stage	Hirst et al., 2020 [14]	UK	Stages 1 and 2
	Average from TriNetX and LCED database analysis by AstraZeneca (data on file)	USA	Stages 3a and 3b
	Bakris et al., 2020 [38]	USA	Stages 4 and 5
**India**			
eGFR	Anand et al., 2015 [87]	-	Disaggregated based on [4]
Albuminuria			
**KRT parameters**			
KRT initiation threshold	Prof. Eiichiro Kanda, personal communication	Japan	
Chance of being treated	UK Renal Registry [88]	UK	

Peritoneal dialysis	Parameswaran et al., 2011 [89]	-	Incidence of patients who are undergoing KRT
	Nitta et al., 2020 [90]<br>Japanese Society for Transplantation, 2020 [91]	Japan [91]	Prevalence of patients who are undergoing KRT calculated from [90] and [91]
Haemodialysis	Bharati et al., 2020 [92]<br>Zhang et al., 2019 [47]<br>(rescaled to India population)	China [47]	Prevalence of KRT per 100 000 by year
Transplantation	Japanese Society for Transplantation, 2020 [91]	Japan	Calculated transplantation rate
	Xue et al., 2017 [50]	China	Risk of transplantation failure
**Comorbidities of CKD**			
Type 2 diabetes	NHANES, 2020 [93]	USA	
Hypertension			
**Cardiovascular complications of CKD**			
Heart failure	Japanese-language paper [52]	Japan	Incidence
	E-Stat Japan [94]	Japan	Prevalence
	USRDS, 2009 [7]	USA	Relative risk
Myocardial infarction	Japanese Registry of All Cardiac and Vascular Diseases [95]	Japan	Incidence
	Ministry of Health, Labour and Welfare, 2019 [96]	Japan	Prevalence
	USRDS, 2009 [7]	USA	Relative risk
Stroke	Das et al., 2006 [97]	-	Incidence and prevalence
	Masson et al., 2015 [10]	-	Relative risk
**Mortality**			
Mortality by CKD stage	UN, World Population Prospects, 2019 [11, 12]	-	Total number of deaths by age and sex
	Levey et al., 2011 [13]	-	Probability of dying from any cause by CKD stage
**Diagnosis rates**			
Diagnosis rates by stage	Hirst et al., 2020 [14]	UK	Average calculated from both inputs
	HSE, 2016 [4]	UK	
**Israel**			
eGFR	Maccabi database, 2019 (database not publicly available)	-	Percentage distribution of eGFR and albuminuria by age and sex
Albuminuria			
**KRT parameters**			
KRT initiation threshold	Pyart et al., 2020 [27]	UK	
Chance of being treated	Prof. Avraham Karasik, personal communication	-	

Peritoneal dialysis	Israel Government Dialysis Report, 2020 [98]	-	Incidence of patients who are undergoing KRT<br><br>Prevalence of patients who are undergoing KRT<br><br>Transplantation rate<br><br>Prevalence of KRT modality per 100 000 by year
Haemodialysis			
Transplantation	Pyart et al., 2020 [27]	UK	Risk of transplantation failure
**Comorbidities of CKD**			
Type 2 diabetes	Maccabi database, 2019 (database not publicly available)	-	Prevalence of type 2 diabetes and hypertension in the CKD population, by albuminuria level
Hypertension			
**Cardiovascular complications of CKD**			
Heart failure	Maccabi database, 2019	-	Incidence and prevalence
	USRDS, 2009 [7]	USA	Relative risk
Myocardial infarction	Maccabi database, 2019	-	Incidence and prevalence
	USRDS, 2009 [7]	USA	Relative risk
Stroke	Maccabi database, 2019	-	Incidence and prevalence
	Masson et al., 2015 [10]	-	Relative risk
**Mortality**			
Mortality by CKD stage	UN, World Population Prospects, 2019 [11, 12]	-	Total number of deaths by age and sex
	Levey et al., 2011 [13]	-	Probability of dying from any cause by CKD stage
**Diagnosis rates**			
Diagnosis rates by stage	Maccabi database, 2019	-	Diagnosis rates provided by agency (database not publicly available)
**Italy**			
eGFR	De Nicola et al., 2015 [99]	-	Percentage distribution of eGFR and albuminuria by age/sex, distributed based on data from France
Albuminuria			
**KRT parameters**			
KRT initiation threshold	Ministero della Salute, 2014 [100]	-	
Chance of being treated	Organización Nacional de Trasplantes, 2018 [28]	Spain	
Peritoneal dialysis	Organización Nacional de Trasplantes, 2018 [28]<br><br>Nordio et al., 2020 [101]	Spain<br><br>-	Incidence of patients who are undergoing KRT<br><br>Prevalence of patients who are undergoing KRT

Haemodialysis	Organización Nacional de Trasplantes, 2018 [28]	Spain	Prevalence of KRT per 100 000 by year
			Transplantation rate per 100 dialysis patients
Transplantation	Grandaliano, 2020 [78]	-	Calculated from 10-year failure rate of 40%
**Comorbidities of CKD**			
Type 2 diabetes	Ruiz-Hurtado et al., 2016 [33]	Spain	Prevalence of type 2 diabetes and hypertension in the CKD population, by albuminuria level
Hypertension			
**Cardiovascular complications of CKD**			
Heart failure	Piccinni et al., 2017 [79]	-	Prevalence calculated from incidence
	USRDS, 2009 [7]	-	Relative risk
Myocardial infarction	Registro dell'infarto miocardico acuto nell'ASL di Brescia [81]	-	Prevalence calculated from incidence
	USRDS, 2009 [7]	USA	Relative risk
Stroke	Corso et al., 2013 [102]	-	Prevalence calculated from incidence
	Masson et al., 2015 [10]	-	Relative risk
**Mortality**			
Mortality by CKD stage	UN, World Population Prospects, 2019 [11, 12]	-	Total number of deaths by age and sex
	Levey et al., 2011 [13]	-	Probability of dying from any cause by CKD stage
**Diagnosis rates**			
Diagnosis rates by stage	Hirst et al., 2020 [14]	UK	Stages 1 and 2
	Average from TriNetX and LCED database analysis by AstraZeneca (data on file)	USA	Stages 3a and 3b
	Bakris et al., 2020 [38]	USA	Stages 4 and 5
**Japan**			
eGFR	Japanese Society of Nephrology, 2012 [45]	-	Percentage distribution of eGFR and albuminuria by age/sex
Albuminuria	HSE, 2016 [4]	UK	Aged <40 years
	Japanese Society of Nephrology, 2012 [45]	-	Aged >40 years: Japan AER albuminuria data by single age category, redistributed based on UK data
**KRT parameters**			
KRT initiation threshold	Prof. Eiichiro Kanda, personal communication		
Chance of being treated	O'Callaghan et al., 2011 [16]	UK	
Peritoneal dialysis	Calculated using Yagisawa et al., 2019 [103] and Nitta et al., 2020 [90]	-	Incidence of patients who are undergoing KRT

Haemodialysis	Calculated using Nitta et al., 2020 [90] and the Japanese Society for Transplantation, 2020 [91]	-	Prevalence of patients who are undergoing KRT
Transplantation	Calculated<br><br>UK Renal Registry (p91) [19]	-<br><br>UK	Transplantation rate<br><br>Risk of transplantation failure
**Comorbidities of CKD**			
Type 2 diabetes	HSE, 2016 [4]	UK	Prevalence of type 2 diabetes and hypertension in the CKD population, by albuminuria level
Hypertension	Barzilay et al., 2018 [20]	UK	
	HSE, 2016 [4]	UK	
**Cardiovascular complications of CKD**			
Heart failure	Japanese-language paper [52]	-	Incidence
	E-Stat Japan [94]	-	Prevalence
	USRDS, 2009 [7]	USA	Relative risk
Myocardial infarction	Japanese-language paper [52]	-	Incidence
	The Japanese Registry of All Cardiac and Vascular Diseases, 2019 [95]; Ministry of Health, Labour and Welfare, 2019 [96]	-	Prevalence
	USRDS, 2009 [7]	USA	Relative risk
Stroke	Takashima et al., 2017 [104]	-	Incidence
	E-Stat Japan [94]	-	Prevalence
	Masson et al., 2015 [10]	-	Relative risk
**Mortality**			
Mortality by CKD stage	UN, World Population Prospects, 2019 [11, 12]	-	Total number of deaths by age and sex
	Levey et al., 2011 [13]	-	Probability of dying from any cause by CKD stage
**Diagnosis rates**			
Diagnosis rates by stage	Hirst et al., 2020 [14]	UK	Average calculated from both inputs
	HSE, 2016 [4]	UK	
**Mexico**			
eGFR	Cueto-Manzano et al., 2014 [105]	-	Percentage distribution of eGFR and albuminuria by age/sex
Albuminuria			
**KRT parameters**			
KRT initiation threshold	USRDS, 2020 [106]	USA	
Chance of being treated	Garcia-Garcia et al., 2007 [107]	-	
Peritoneal dialysis	USRDS, 2020 [108]	USA	Incidence of patients who are undergoing KRT

Haemodialysis<br><br>Transplantation	USRDS, 2020 - average from both regions [106] (Jalisco and Aguascalientes)<br><br>USRDS, 2021 [40]<br><br>USRDS, 2020 [106]	USA<br><br>USA<br><br>USA	Prevalence of KRT by year<br><br>Prevalence of patients who are undergoing KRT<br><br>Transplantation rate<br><br>Risk of transplantation failure
**Comorbidities of CKD**			
Type 2 diabetes<br><br>Hypertension	Ruiz-Hurtado et al., 2016 [33]	Spain	Prevalence of type 2 diabetes and hypertension in the CKD population, by albuminuria level
**Cardiovascular complications of CKD**			
Heart failure	Fernández-Gassó et al., 2019 [34]	Spain	Incidence
	Farré et al., 2017 [108]	-	Prevalence
	USRDS, 2009 [7]	USA	Relative risk
Myocardial infarction	IHME Global Burden of Diseases, 2019 [25]	-	Incidence and Prevalence
	USRDS, 2009 [7]	USA	Relative risk
Stroke	IHME Global Burden of Diseases, 2019 [25]	-	Incidence and prevalence
	Masson et al., 2015 [10]	-	Relative risk
**Mortality**			
Mortality by CKD stage	UN, World Population Prospects, 2019 [11, 12]	-	Total number of deaths by age and sex
	Levey et al., 2011 [13]	-	Probability of dying from any cause by CKD stage
**Diagnosis rates**			
Diagnosis rates by stage	Hirst et al., 2020 [14]	UK	Stages 1 and 2
	Average from TriNetX and LCED database analysis by AstraZeneca (data on file)	USA	Stages 3a and 3b
	Bakris et al., 2020 [38]	USA	Stages 4 and 5
**Netherlands**			
eGFR	HSE, 2016 [4]	UK	Stages by 5-year age categories
Albuminuria	HSE, 2016 [4]	UK	Stages for four age groups
**KRT parameters**			
KRT initiation threshold	Dutch guideline for diabetic nephropathy, 2020 [109]	-	
Chance of being treated	O'Callaghan et al., 2011 [16]	UK	
Peritoneal dialysis<br><br>Haemodialysis	Nefrovisie, 2022 [110]	-	Incidence and prevalence of patients receiving KRT who were undergoing peritoneal dialysis and haemodialysis<br><br>Prevalence of KRT by year

Transplantation	O'Callaghan et al., 2011 [16]	-	Transplantation rate per 100 dialysis patients per year<br><br>Annual risk of transplantation failure
**Comorbidities of CKD**			
Type 2 diabetes	HSE, 2016 [4]	UK	Prevalence of type 2 diabetes and hypertension in the CKD population by albuminuria level
Hypertension	HSE, 2016 [4]	UK	
**Cardiovascular complications of CKD**			
Heart failure	Dutch Heart Foundation, 2021 [111]	-	Incidence and prevalence
	USRDS, 2009 [7]	USA	Relative risk
Myocardial infarction	Dutch Heart Foundation, 2021 [111]	-	Incidence and prevalence
	USRDS, 2009 [7]	USA	Relative risk
Stroke	Dutch Heart Foundation, 2021 [111]	-	Incidence and prevalence
	Masson et al., 2015 [10]	-	Relative risk
**Mortality**			
Mortality by CKD stage	UN, World Population Prospects, 2019 [11, 12]	-	Total number of deaths by age and sex
	Levey et al., 2011 [13]	-	Probability of dying from any cause by CKD stage
**Diagnosis rates**			
Diagnosis rates by stage	Hirst et al., 2020 [14]	UK	Average calculated from both inputs
	HSE, 2016 [4]	UK	
**Philippines**			
eGFR	Nang et al., 2009 [112]	Singapore	Percentage prevalence of each eGFR stage, disaggregated by age and sex using data from UK and China
Albuminuria	Piccolli et al., 2017 [26]	Brazil	Percentage distribution of eGFR and albuminuria by age/sex
**KRT parameters**			
KRT initiation threshold	Watanabe et al., 2015 [46]	Japan	
Chance of being treated	O'Callaghan et al., 2011 [16]	UK	
Peritoneal dialysis	USRDS, 2020, ADR international comparisons [113]	USA	Incidence and prevalence of patients receiving KRT who were undergoing peritoneal dialysis and haemodialysis, and prevalence of KRT by year
Haemodialysis			
Transplantation	Chinese National Renal Data System [48]<br><br>Xue et al., 2017 [50]	China<br><br>China	Transplantation rate per 100 dialysis patients per year<br><br>Risk of transplantation failure

**Comorbidities of CKD**			
Type 2 diabetes	HSE, 2016 [4]	UK	Categorized by albuminuria level
Hypertension	HSE, 2016 [4]	UK	
	Barzilay et al., 2018 [20]	UK	
**Cardiovascular complications of CKD**			
Heart failure	Japanese-language paper [52]	Japan	Incidence
	E-Stat Japan [94]	Japan	Prevalence
	USRDS, 2009 [7]	USA	Relative risk
Myocardial infarction	Japanese-language paper [52]	Japan	Incidence
	The Japanese Registry of All Cardiac and Vascular Diseases, 2019 [95]; Ministry of Health, Labour and Welfare, 2019 [96]	Japan	Prevalence
	USRDS, 2009 [7]	USA	Relative risk
Stroke	IHME Global Burden of Diseases, 2019 [25]	-	Incidence and prevalence
	Masson et al., 2015 [10]	-	Relative risk
**Mortality**			
Mortality by CKD stage	UN, World Population Prospects, 2019 [11, 12]	-	Total number of deaths by age and sex
	Levey et al., 2011 [13]	-	Probability of dying from any cause by CKD stage
**Diagnosis rates**			
Diagnosis rates by stage	Wong et al., 2018 [114]	Singapore	
**Poland**			
eGFR	Zdrojewski et al., 2015 [84]	-	eGFR data by age and two risk categories, disaggregated using UK data. Albuminuria data by age and risk category
Albuminuria	Chudek et al., 2014 [85]	-	
**KRT parameters**			
KRT initiation threshold	Myśliwiec, 2021 [115]	-	
Chance of being treated	NHF CKD, 2020 [116]<br>Dębska-Ślizień et al., 2020 [117]	-<br>-	Chance of being treated, incidence and prevalence of patients receiving KRT who were undergoing dialysis, and prevalence of KRT by year
Peritoneal dialysis			
Haemodialysis			Rate per 100 dialysis patients per year
Transplantation	ANZDATA 43rd Annual Report, 2020 [3]	Australia	Annual risk of transplantation failure
**Comorbidities of CKD**			
Type 2 diabetes	HSE, 2016 [4]	UK	Prevalence of type 2 diabetes and hypertension in the CKD population, by albuminuria level
Hypertension	Barzilay et al., 2018 [20]	UK	

	HSE, 2016 [4]	UK	
**Cardiovascular complications of CKD**			
Heart failure	MPZ Kardiologia, 2018 [118]	-	Incidence
	National inpatient claims database analysis (data on file)	Romania	Prevalence
	USRDS, 2009 [7]	USA	Relative risk
Myocardial infarction	National inpatient claims database analysis (data on file)	Romania	Incidence
	Dutch Heart Foundation, 2021 [111]	Netherlands	Prevalence
	USRDS, 2009 [7]	USA	Relative risk
Stroke	National inpatient claims database analysis (data on file)	Romania	Incidence
	Dutch Heart Foundation, 2021 [111]	Netherlands	Prevalence
	Masson et al., 2015 [10]	-	Relative risk
**Mortality**			
Mortality by CKD stage	UN, World Population Prospects, 2019 [11, 12]	-	Total number of deaths by age and sex
	Levey et al., 2011 [13]	-	Probability of dying from any cause by CKD stage
**Diagnosis rates**			
Diagnosis rates by stage	Hirst et al., 2020 [14]	UK	Average calculated from both inputs
	HSE, 2016 [4]	UK	
**Romania**			
eGFR	Zdrojewski et al., 2015 [84]	Poland	eGFR data by age and two risk categories, disaggregated using UK data
Albuminuria	Chudek et al., 2014 [85]	Poland	Albuminuria data by age and risk category
**KRT parameters**			
KRT initiation threshold	Myśliwiec, 2021 [115]	Poland	
Chance of being treated	O'Callaghan et al., 2011 [16]	UK	Divided by the results of a calculation that estimated the percentage of the population of the UK undergoing KRT
Peritoneal dialysis	Spitalul Clinic de Nefrologie Carol Davila, 2022 [119]	-	Incidence and prevalence of patients receiving KRT who are undergoing dialysis
Haemodialysis	Haemodialysis and peritoneal dialysis: Romanian Renal Registry Report, 2019–2020 [120]	-	Prevalence of KRT by year

Transplantation	ERA-EDTA annual reports [121]<br><br>Spitalul Clinic de Nefrologie Carol Davila, 2017 [122]<br><br>Analysis from national inpatient claims database (data on file)	-<br><br>-<br><br>-	Prevalence of KRT by year<br><br>Rate per 100 dialysis patients per year<br><br>Annual risk of transplantation failure
**Comorbidities of CKD**			
Type 2 diabetes	Analysis from national inpatient claims database (data on file)	-	Prevalence of type 2 diabetes and hypertension in the CKD population, by albuminuria level
Hypertension			
**Cardiovascular complications of CKD**			
Heart failure	MPZ Kardiologia, 2018 [118]	Poland	Incidence
	National inpatient claims database analysis (data on file)	-	Prevalence
	USRDS, 2009 [7]	USA	Relative risk
Myocardial infarction	National inpatient claims database analysis (data on file)	-	Incidence
	Dutch Heart Foundation, 2021 [111]	Netherlands	Prevalence
	USRDS, 2009 [7]	USA	Relative risk
Stroke	National inpatient claims database analysis (data on file)	-	Incidence
	Dutch Heart Foundation, 2021 [111]	Netherlands	Prevalence
	Masson et al., 2015 [10]	-	Relative risk
**Mortality**			
Mortality by CKD stage	UN, World Population Prospects, 2019 [11, 12]	-	Total number of deaths by age and sex
	Levey et al., 2011 [13]	-	Probability of dying from any cause by CKD stage
**Diagnosis rates**			
Diagnosis rates by stage	Hirst et al., 2020 [14]	UK	Average calculated from both inputs
	HSE, 2016 [4]	UK	
**Saudi Arabia**			
eGFR	Ahmed et al., 2014 [123]	-	Percentage distribution of eGFR and albuminuria by age/sex
Albuminuria	NHANES, 2020 [93]	USA	
**KRT parameters**			
KRT initiation threshold	USRDS, 2020 [113]	USA	
Chance of being treated	Organización Nacional de Trasplantes, 2018 [28]	Spain	
Peritoneal dialysis	Al-Attar, 2021 [124]	-	Incidence and prevalence of patients receiving KRT who are undergoing dialysis

Haemodialysis	Saudi Center for Organ Transplantation, 2019 [125]	-	Prevalence of KRT by modality
Transplantation	Calculated from Neves et al., 2021 [31]	Brazil	Transplantation rate
	UK Renal Registry (p91) [19]	UK	Risk of transplantation failure
**Comorbidities of CKD**			
Type 2 diabetes	NHANES, 2020 [93]	USA	Prevalence of type 2 diabetes and hypertension in the CKD population by albuminuria level
Hypertension			
**Cardiovascular complications of CKD**			
Heart failure	Benjamin et al., 2019 [126]	USA	Incidence calculated from prevalence
	USRDS, 2009 [7]	USA	Relative risk
Myocardial infarction	Mozaffarian et al., 2015 [127]	USA	Incidence calculated from prevalence
	USRDS, 2009 [7]	USA	Relative risk
Stroke	Alhazzani et al., 2018 [128]	-	Incidence
	IHME Global Burden of Diseases, 2019 [25]	-	Prevalence
	Masson et al., 2015 [10]	-	Relative risk
**Mortality**			
Mortality by CKD stage	UN, World Population Prospects, 2019 [11, 12]	-	Total number of deaths by age and sex
	Levey et al., 2011 [13]	-	Probability of dying from any cause by CKD stage
**Diagnosis rates**			
Diagnosis rates by stage	Hirst et al., 2020 [14]	UK	Stages 1 and 2
	Average from TriNetX and LCED database analysis by AstraZeneca (data on file)	USA	Stages 3a and 3b
	Bakris et al., 2020 [38]	USA	Stages 4 and 5
**Singapore**			
eGFR	Nang et al., 2009 [112]	-	Disaggregated into more granular eGFR stages using data from China, and disaggregated by age and sex using UK data
Albuminuria	Nang et al., 2009 [112]	-	Disaggregated into more granular AER stages using data from South Korea, and disaggregated by age and sex using UK data
**KRT parameters**			
KRT initiation threshold	Watanabe et al., 2015 [46]	Japan	
Chance of being treated	Singapore Renal Registry Annual Report, 2018 [129]	-	Chance of being treated, incidence and prevalence of patients receiving KRT who

Peritoneal dialysis			are undergoing dialysis, and prevalence of KRT by year
Haemodialysis			
Transplantation	Chinese National Renal Data System [48]<br><br>Singapore Renal Registry Annual Report, 2018 [129]	China<br><br>-	Rate per 100 dialysis patients per year<br><br>Annual risk of transplantation failure
**Comorbidities of CKD**			
Type 2 diabetes	HSE, 2016 [4]	UK	Prevalence of type 2 diabetes and hypertension in the CKD population, by albuminuria level
Hypertension	Barzilay et al., 2018 [20]	UK	
	HSE, 2016 [4]	UK	
**Cardiovascular complications of CKD**			
Heart failure	Calculated		Incidence
	Sun et al., 2009 [51]	China	Prevalence
	USRDS, 2009 [7]	USA	Relative risk
Myocardial infarction	Singapore Myocardial Infarction Registry Annual Report, 2018 [130]	-	Incidence
	Calculated	-	Prevalence
	USRDS, 2009 [7]	USA	Relative risk
Stroke	IHME Global Burden of Diseases, 2019 [25]	-	Incidence and prevalence
	Masson et al., 2015 [10]	-	Relative risk
**Mortality**			
Mortality by CKD stage	UN, World Population Prospects, 2019 [11, 12]	-	Total number of deaths by age and sex
	Levey et al., 2011 [13]	-	Probability of dying from any cause by CKD stage
**Diagnosis rates**			
Diagnosis rates by stage	Wong et al., 2018 [114]	-	
**South Korea**			
eGFR	Park et al., 2016 [131]<br><br>Kim et al., 2009 [132]	-<br><br>-	Percentage distribution of eGFR and albuminuria by age/sex (≥0 years with each stage of CKD)
Albuminuria			Percentage distribution of eGFR and albuminuria by age/sex (≥18 years with each stage of albuminuria)

**KRT parameters**			
KRT initiation threshold	Assumption		
Chance of being treated	O'Callaghan et al., 2011 [16]	UK	
Peritoneal dialysis	Korean Society of Nephrology [133]	-	Incidence and prevalence of patients receiving KRT who are undergoing dialysis
Haemodialysis	Korean Society of Nephrology [133]	-	Transplantation rate per 100 dialysis patients per year
	Kim and Jin, 2020 [134]	-	Prevalence of KRT by modality
Transplantation	Park et al., 2020 [135]	-	Risk of transplantation failure
**Comorbidities of CKD**			
Type 2 diabetes	HSE, 2016 [4]	UK	Prevalence of type 2 diabetes and hypertension in the CKD population, by albuminuria level
Hypertension	HSE, 2016 [4]	-	
	Barzilay et al., 2018 [20]	UK	
**Cardiovascular complications of CKD**			
Heart failure	National Health Interview Survey, 2018 [136]	-	Incidence
	Lee et al., 2016 [137]	-	Prevalence
	USRDS, 2009 [7]	USA	Relative risk
Myocardial infarction	Kim et al., 2019 [138]	-	Prevalence calculated from incidence
	USRDS, 2009 [7]	USA	Relative risk
Stroke	Korea Disease Control and Prevention Agency, 2018 [139]	-	Incidence and prevalence
	Masson et al., 2015 [10]	-	Relative risk
**Mortality**			
Mortality by CKD stage	UN, World Population Prospects, 2019 [11, 12]	-	Total number of deaths by age and sex
	Levey et al., 2011 [13]	-	Probability of dying from any cause by CKD stage
**Diagnosis rates**			
Diagnosis rates by stage	Hirst et al., 2020 [14]	UK	Average calculated from both inputs
	HSE, 2016 [4]	UK	
**Spain**			
eGFR	Otero et al., 2010 [53]	-	Categorical eGFR data, redistributed based on UK data
Albuminuria	Gorostidi et al., 2018 [54]	-	Categorical albuminuria data for both sexes, by age, redistributed based on UK data

**KRT parameters**			
KRT initiation threshold	Prof. Juan Francisco Navarro-González, personal communication	-	
Chance of being treated	Organización Nacional de Trasplantes, 2018 [28]	-	Incidence and prevalence of patients receiving KRT who are undergoing dialysis, and prevalence of KRT by modality<br>Transplantation rate per dialysis patient-year<br>Risk of transplantation failure
Peritoneal dialysis			
Haemodialysis			
Transplantation			
**Comorbidities of CKD**			
Type 2 diabetes	Ruiz-Hurtado et al., 2016 [33]	-	Proportion of type 2 diabetes and hypertension in the CKD population, by albuminuria level
Hypertension			
**Cardiovascular complications of CKD**			
Heart failure	Fernández-Gassó et al., 2019 [34]	-	Incidence
	Farré et al., 2017 [108]	-	Prevalence
	USRDS, 2009 [7]	USA	Relative risk
Myocardial infarction	Forcadell et al., 2018 [36]	-	Incidence, prevalence calculated
	USRDS, 2009 [7]	USA	Relative risk
Stroke	Vega et al., 2009 [37]	-	Incidence, prevalence calculated
	Masson et al., 2015 [10]	-	Relative risk
**Mortality**			
Mortality by CKD stage	Instituto Nacional de Estadistica, 2020 [141]	-	Number of deaths by age
	Levey et al., 2011 [13]	-	Probability of dying from any cause by CKD stage
**Diagnosis rates**			
Diagnosis rates by stage	Hirst et al., 2020 [14]	UK	Stages 1 and 2
	Average from TriNetX and LCED database analysis by AstraZeneca (data on file)	USA	Stages 3a and 3b
	Bakris et al., 2020 [38]	USA	Stages 4 and 5
**Sweden**			
eGFR	Analysis of SCREAM data (study information: Mazhar et al., 2022 [in press]); data on file	-	Categorized by age and sex
Albuminuria			
**KRT parameters**			
KRT initiation threshold	Prof. Johan Ärnlöv, personal communication	-	

Chance of being treated	Frei et al., 2008 [74]	Germany	
Peritoneal dialysis	Svenskt Njurregister, 2021 [141]	-	Incidence and prevalence of patients receiving KRT who are undergoing dialysis, and prevalence of KRT by year
Haemodialysis			
Transplantation	Jaarrapport, 2019 [18]<br><br>Njurförbundet, 2021 [142]	Belgium<br><br>-	Transplantation rate per dialysis patient-year<br><br>Annual risk of transplantation failure
**Comorbidities of CKD**			
Type 2 diabetes	CELOSIA analysis, 2019 (data on file)	-	Proportion of type 2 diabetes and hypertension in the CKD population, by albuminuria level
Hypertension			
**Cardiovascular complications of CKD**			
Heart failure	Analysis of SCREAM data (study information: Mazhar et al., 2022 [in press]); data on file	-	Incidence and prevalence
	USRDS, 2009 [7]	USA	Relative risk
Myocardial infarction	Analysis of SCREAM data (study information: Mazhar et al., 2022 [in press]); data on file	-	Incidence and prevalence
	USRDS, 2009 [7]	USA	Relative risk
Stroke	Analysis of SCREAM data (study information: Mazhar et al., 2022 [in press]); data on file	-	Incidence and prevalence
	Masson et al., 2015 [10]	-	Relative risk
**Mortality**			
Mortality by CKD stage	UN, World Population Prospects, 2019 [11, 12]	-	Total number of deaths by age and sex
	Levey et al., 2011 [13]	-	Probability of dying from any cause by CKD stage
**Diagnosis rates**			
Diagnosis rates by stage	CELOSIA analysis, 2019 (data on file)	-	
**Taiwan**			
eGFR	Kweon et al., 2014 [143]	South Korea	Mean eGFR and albuminuria by age category and sex
Albuminuria			
**KRT parameters**			
KRT initiation threshold	Prof. Mai-Szu Wu, personal communication	-	
Chance of being treated	O'Callaghan et al., 2011 [16]	UK	
Peritoneal dialysis	Taiwan Society of Nephrology, 2019 [144]	-	Incidence and prevalence of patients receiving KRT who

Haemodialysis			are undergoing dialysis, and prevalence of KRT by year
Transplantation			Transplantation rate per dialysis patient-year<br><br>Annual risk of transplantation failure
**Comorbidities of CKD**			
Type 2 diabetes	Lin et al., 2007 [145]	South Korea	Proportion of type 2 diabetes and hypertension in the CKD population, by albuminuria level
Hypertension	Han et al., 2015 [146]	South Korea	
**Cardiovascular complications of CKD**			
Heart failure	Ministry of Health and Welfare, 2013 [147]	-	Incidence and prevalence
	USRDS, 2009 [7]	USA	Relative risk
Myocardial infarction	Wang et al., 2019 [148]	-	Incidence
	Siqeura and De Souza, 2020 [149]	-	Prevalence
	USRDS, 2009 [7]	USA	Relative risk
Stroke	Ministry of Health and Welfare, 2013 [147]	-	Incidence, prevalence
	Masson et al., 2015 [10]	-	Relative risk
**Mortality**			
Mortality by CKD stage	UN, World Population Prospects, 2019 [11, 12]	-	Total number of deaths by age and sex
	Levey et al., 2011 [13]	-	Probability of dying from any cause by CKD stage
**Diagnosis rates**			
Diagnosis rates by stage	Hirst et al., 2020 [14]	UK	Average calculated from both inputs
	HSE, 2016 [4]	UK	
**Thailand**			
eGFR	Perkovic et al., 2008 [150]	-	Prevalence of eGFR stage by age group
Albuminuria	Zhang et al., 2012 [44]	China	Categorical albuminuria data for both sexes, by age
	Japanese Society of Nephrology [45]	Japan	
**KRT parameters**			
KRT initiation threshold	Assoc. Prof. Jason Choo Chon Jun, personal communication	-	
Chance of being treated	O'Callaghan et al., 2011 [16]	UK	
Peritoneal dialysis	Nephrology Society of Thailand [151]	-	Incidence and prevalence of patients receiving KRT who are undergoing dialysis, and prevalence of KRT by year
Haemodialysis			
Transplantation	Chinese National Renal Data System [48]<br><br>Xue et al., 2017 [50]	China<br><br>China	Transplantation rate

			Risk of transplantation failure
**Comorbidities of CKD**			
Type 2 diabetes	HSE, 2016 [4]	UK	Prevalence of type 2 diabetes and hypertension in the CKD population, by albuminuria level
Hypertension	HSE, 2016 [4]	UK	
	Barzilay et al., 2018 [20]	UK	
**Cardiovascular complications of CKD**			
Heart failure	Japanese-language paper [52]	Japan	Incidence
	E-Stat Japan [94]	-	Prevalence
	USRDS, 2009 [7]	USA	Relative risk
Myocardial infarction	Japanese-language paper [52]	Japan	Incidence
	The Japanese Registry of All Cardiac and Vascular Diseases, 2019 [96]; Ministry of Health, Labour and Welfare, 2019 [96]	-	Prevalence
	USRDS, 2009 [7]	USA	Relative risk
Stroke	IHME Global Burden of Diseases, 2019 [25]	-	Incidence and prevalence
	Masson et al., 2015 [10]	-	Relative risk
**Mortality**			
Mortality by CKD stage	UN, World Population Prospects, 2019 [11, 12]	-	Total number of deaths by age and sex
	Levey et al., 2011 [13]	-	Probability of dying from any cause by CKD stage
**Diagnosis rates**			
Diagnosis rates by stage	Wong et al., 2018 [114]	Singapore	
**Türkiye**			
eGFR	Süleymanlar et al., 2011 [152]	-	Categorical eGFR data for adults, disaggregated according to Spain proxy data, and distributed by age and sex according to US proxy data
Albuminuria			Categorical albuminuria data for both sexes, by age
**KRT parameters**			
KRT initiation threshold	Prof. Bülent Altun, personal communication	-	Hacettepe University, Department of Nephrology, May 2021
Chance of being treated	Registry of Nephrology, 2014 [153]	-	
Peritoneal dialysis	Registry of Nephrology, 2014 [153]	-	Incidence and prevalence of patients receiving KRT who are undergoing dialysis<br>Prevalence of KRT by year
Haemodialysis			

Transplantation	Organización Nacional de Trasplantes, 2018 [28]	Spain	Transplantation rate per 100 dialysis patients per year<br><br>Risk of transplantation failure
**Comorbidities of CKD**			
Type 2 diabetes	HSE, 2016 [4]	UK	Prevalence of type 2 diabetes and hypertension in the CKD population, by albuminuria level
Hypertension	HSE, 2016 [4]	UK	
	Barzilay et al., 2018 [20]	UK	
**Cardiovascular complications of CKD**			
Heart failure	Fernández-Gassó et al., 2019 [34]	Spain	Incidence
	Farré et al., 2017 [108]	-	Prevalence
	USRDS, 2009 [7]	USA	Relative risk
Myocardial infarction	Forcadell et al., 2018 [36]	Spain	Incidence (prevalence calculated)
	USRDS, 2009 [7]	USA	Relative risk
Stroke	Vega et al., 2009 [37]	Spain	Incidence
	Díaz-Guzmán et al., 2008 [154]	-	Prevalence
	Masson et al., 2015 [10]	-	Relative risk
**Mortality**			
Mortality by CKD stage	UN, World Population Prospects, 2019 [11, 12]	-	Total number of deaths by age and sex
	Levey et al., 2011 [13]	-	Probability of dying from any cause by CKD stage
**Diagnosis rates**			
Diagnosis rates by stage	Hirst et al., 2020 [14]	UK	Average calculated from both inputs
	HSE, 2016 [4]	UK	
**UAE (Emirati and migrant populations)***			
eGFR	**Emirati:** Ahmed et al., 2014 [123]	Saudi Arabia	Categorical eGFR by age
	**Migrant:** Anand et al., 2015 [54]	India	KDIGO grid
Albuminuria	**Emirati:** NHANES, 2020 [93]	USA	Individual-level continuous albuminuria data
	**Migrant:** Anand et al., 2015 [87]	India	KDIGO grid
**KRT parameters**			
KRT initiation threshold	O'Callaghan et al., 2011 [16]	UK	
Chance of being treated			
Peritoneal dialysis	Al-Attar, 2021 [124]	Saudi Arabia	Incidence of patients who are undergoing KRT
Haemodialysis	Saudi Center for Organ Transplantation, 2019 [125]	Saudi Arabia	Prevalence of patients who are undergoing KRT, and prevalence of KRT by year

Transplantation	Prof. Stephen Holt, personal communication	-	Transplantation rate per dialysis patient-year
	O'Callaghan et al., 2011 [16]	-	Annual risk of transplantation failure
**Comorbidities of CKD**			
Type 2 diabetes	NHANES, 2018 [155]	USA	Prevalence of type 2 diabetes and hypertension in the CKD population, by albuminuria level
Hypertension			
**Cardiovascular complications of CKD**			
Heart failure	Calculated from prevalence	-	Incidence
	Benjamin et al., 2019 [126]	USA	Prevalence
	USRDS, 2009 [7]	USA	Relative risk
Myocardial infarction	Calculated from prevalence	-	Incidence
	Mozaffarian et al., 2015 [127]	USA	Prevalence
	USRDS, 2009 [7]	USA	Relative risk
Stroke	IHME Global Burden of Diseases, 2019 [25]	-	Incidence and prevalence
	Masson et al., 2015 [10]	-	Relative risk
**Mortality**			
Mortality by CKD stage	UN, World Population Prospects, 2019 [11, 12]	-	Total number of deaths by age and sex
	Levey et al., 2011 [13]	-	Probability of dying from any cause by CKD stage
**Diagnosis rates**			
Diagnosis rates by stage	Hirst et al., 2020 [14]	UK	Stages 1 and 2
	Average from TriNetX and LCED database analysis by AstraZeneca (data on file)	USA	Stages 3a and 3b
	Bakris et al., 2020 [38]	USA	Stages 4 and 5
**UK**			
eGFR	HSE, 2016 [4]	-	Categorical eGFR and albuminuria data for both sexes, by age
Albuminuria			
**KRT parameters**			
KRT initiation threshold	Dr Albert Power, personal communication	-	
Chance of being treated	O'Callaghan et al., 2011 [16]	-	
Peritoneal dialysis	UK Renal Registry [88]	-	Incidence and prevalence of patients receiving KRT who are undergoing dialysis
Haemodialysis	Pyart et al., 2020 [27]	-	KRT prevalence by year
Transplantation	USRDS, 2021 [40]	USA	Transplantation rate per 100 dialysis patients per year
	UK Renal Registry (p91) [19]	-	Risk of transplantation failure

**Comorbidities of CKD**			
Type 2 diabetes	HSE, 2016 [4]	-	Prevalence of type 2 diabetes and hypertension in the CKD population, by albuminuria level
Hypertension			
**Cardiovascular complications of CKD**			
Heart failure	British Heart Foundation, 2020 [5]	USA	Incidence and prevalence
Myocardial infarction	USRDS, 2009 [7]	USA	Relative risk
Stroke	British Heart Foundation, 2020 [5]	-	Incidence and prevalence
	Masson et al., 2015 [10]	-	Relative risk
**Mortality**			
Mortality by CKD stage	UN, World Population Prospects, 2019 [11, 12]	-	Total number of deaths by age and sex
	Levey et al., 2011 [13]	-	Probability of dying from any cause by CKD stage
**Diagnosis rates**			
Diagnosis rates by stage	Hirst et al., 2020 [14]	-	Average calculated from both inputs
	HSE, 2016 [4]	-	
**USA**			
eGFR	NHANES, 2020 [93]	-	Individual-level continuous eGFR and albuminuria data
Albuminuria			
**KRT parameters**			
KRT initiation threshold	Prof. Glenn Chertow, personal communication	-	
Chance of being treated	O'Callaghan et al., 2011 [16]	UK	
Peritoneal dialysis	USRDS, 2021 [156]	-	Incidence and prevalence of patients receiving KRT who are undergoing dialysis
Haemodialysis			KRT prevalence by year<br>Transplantation rate
Transplantation	UK Renal Registry (p91) [19]	UK	Risk of transplantation failure
**Comorbidities of CKD**			
Type 2 diabetes	NHANES, 2020 [93]	-	
Hypertension			
**Cardiovascular complications of CKD**			
Heart failure	Benjamin et al., 2019 [126]	-	Prevalence<br>Incidence estimated from prevalence and mortality [6]
	USRDS, 2009 [7]	-	Relative risk
Myocardial infarction	Mozaffarian et al., 2015 [127]	-	Prevalence<br>Incidence estimated from prevalence and mortality [6]

	USRDS, 2009 [7]	-	Relative risk
Stroke	Benjamin et al., 2019 [126]	-	Prevalence<br><br>Incidence estimated from prevalence and mortality [6]
	Masson et al., 2015 [10]	-	Relative risk
**Mortality**			
Mortality by CKD stage	UN, World Population Prospects, 2019 [11, 12]	-	Total number of deaths by age and sex
	Levey et al., 2011 [13]	-	Probability of dying from any cause by CKD stage
**Diagnosis rates**			
Diagnosis rates by stage	Hirst et al., 2020 [14]	UK	Stages 1 and 2
	Average from TriNetX and LCED database analysis by AstraZeneca (data on file)	-	Stages 3a and 3b
	Bakris et al., 2020 [38]	-	Stages 4 and 5

*UAE migrant population was modelled separately from the Emirati population owing to distinct population demographics requiring separate input data

ADR=annual data report. AER=albumin excretion rate. ANZDATA=Australia and New Zealand Dialysis and Transplant Registry. CKD=chronic kidney disease. eGFR=estimated glomerular filtration rate. ERA-EDTA=European Renal Association - European Dialysis and Transplant Association. GBE=Gesundheitsberichterstattung. HSE=Health and Safety Executive. IHME=Institute for Health Metrics and Evaluation. KDIGO=Kidney Disease: Improving Global Outcomes. KRT=kidney replacement therapy. LCED=Limited Claims and Electronic Health Record. MANET= Magyar Nephrologiai Társaság. MPZ=Mapa potrzeb zdrowotnych. NHANES=National Health and Nutrition Examination Survey. NHF=National Health Fund. NHIFA=National Health Insurance Fund Administration. REIN= Renal Epidemiology and Information Network. SISPRO=Sistema Integrado de Información de la Protección Social. THIN=The Health Improvement Network. UACR=urine albumin:creatinine ratio. UN=United Nations. USRDS=United States Renal Data System.

Table S3: External validation of clinical burden of disease outputs

Output	External validation reference	External validation value	*Inside CKD* 2022 value	External validation notes
**Australia**				
CKD prevalence	Australia Health Survey 2012 [1]	11 000 per 100 000 (aged ≥18 years)	8917 per 100 000	
CKD prevalence change	Australian Institute of Health and Welfare 2018 [2]	24% (Stages 1–5) 42% (Stages 3a–5)	6·6% (2022–2027)	1999–2012
Mortality prevalence	Bikbov et al., 2020 [3]	9·2 per 100 000 (8·3–10·1)	120 per 100 000 (diagnosed patients with CKD)	Age-standardized rate in 2017 (95% CI)
Mortality prevalence change	Australian Institute of Health and Welfare 2020 [4]	56·2–52·1 per 100 000	−7·2% (2022–2027 in diagnosed patients with CKD)	2010–2018
Heart failure prevalence	Conrad et al., 2018 [5]: UK data	24 455 per 100 000 (CKD population)	20 407 per 100 000 (diagnosed patients with CKD)	
Myocardial infarction prevalence	Meisinger et al., 2006 [6]: German data	1465 per 100 000 (male) 482 per 100 000 (female) (CKD population)	5230 per 100 000 (diagnosed patients with CKD)	Presented in person-years and recalculated
Stroke prevalence	Weiner et al., 2004 [7]	10 896 per 100 000 (CKD population)	5064 per 100 000 (diagnosed patients with CKD)	
**Belgium**				
CKD prevalence	Bikbov et al., 2020 [3]	5642 per 100 000 (5238–6088)	14 186 per 100 000	Age-standardized rate in 2017 (95% CI)
CKD prevalence change	Bikbov et al., 2020 [3]	−1·4% (−4·9–1·9%)	−0·9% (2022–2027)	Change in age-standardized rates, 1990–2017 (95% CI)
Mortality prevalence	Bikbov et al., 2020 [3]	7·2 per 100 000 (6·7–7·7)	391 per 100 000 (diagnosed patients with CKD)	Age-standardized rate (95% CI).Vital registration and verbal autopsy data were used to model mortality due to CKD. Data were standardized and mapped when each death was assigned to a single underlying cause
Mortality prevalence change	Bikbov et al., 2020 [3]	−29·4% (−34·9 to −23·4%)	−25·6% (2022–2027 in diagnosed patients with CKD)	Change in age-standardized rates, 1990–2017 (95% CI)
Heart failure prevalence	Heart Failure Policy Network 2020 [8]	1–3% (total population)	6908 per 100 000 (diagnosed patients with CKD)	
Stroke prevalence	World Bank 1993 [9]	1876 per 100 000 (total population)	4851 per 100 000 (diagnosed patients with CKD)	Population aged 45–54 years (based on estimates from the WHO Global Burden of Disease 2000 study)
**Brazil**				
CKD prevalence	Barreto et al., 2016 [10]	8·9%	9·7% of national population	According to baseline data of 14 636 public sector employees aged 35–74 years; 2008–2010
	Piccolli et al., 2017 [11]	11·4%		5216 individuals randomly selected from a pool of 10 000 from the database of a local energy company
Mortality prevalence	Bikbov et al., 2020 [3]	16·1 per 100 000 (15·8–16·5)	200 per 100 000 (diagnosed patients with CKD)	Age-standardized rate (95% CI). Vital registration and verbal autopsy data were used

				to model mortality due to CKD. Data were standardized and mapped when each death was assigned to a single underlying cause
Mortality prevalence change	Souza et al., 2019 [12]	2·79–3·14 per 100 000	−4·7% (2022–2027 in diagnosed patients with CKD)	1996–2016, conducted in a single Brazilian state
Heart failure prevalence	Stevens et al., 2018 [13]	2·0% (total adult population)	8154 per 100 000 (diagnosed patients with CKD)	
Myocardial infarction prevalence	Stevens et al., 2018 [13]	0·2% (total adult population)	3432 per 100 000 (diagnosed patients with CKD)	
Stroke prevalence	Bensenor et al., 2015 [14]	1·6% (male) 1·4% (female) (total population)	3124 per 100 000 (diagnosed patients with CKD)	From the Brazilian National Health Survey based on self-reported data. 60 202 individuals were interviewed
**Canada**				
CKD prevalence	Arora et al., 2013 [15]	12 500 per 100 000	13 344 per 100 000	12·5% of the population aged 6–79 years, 2007–2009
CKD prevalence change	Bello et al., 2019 [16]	5440–4650 per 100 000	13 344–13 649 per 100 000 (2022–2027)	Study included 559 745 adults in primary healthcare in five provinces: 2010–2015
Mortality prevalence	Bikbov et al., 2020 [3]	7·9 per 100 000 (7·4–8·5)	259 per 100 000 (diagnosed patients with CKD)	Age-standardized rate (95% CI). Vital registration and verbal autopsy data were used to model mortality due to CKD. Data were standardized and mapped when each death was assigned to a single underlying cause
Heart failure prevalence	Björn et al., 2015 [17]	669 620 cases (total population)	10 197 per 100 000 (diagnosed patients with CKD)	Population aged ≥40 years, 2012–2013
Myocardial infarction prevalence	Statista 2019 [18]	1·2–2·0% (total population)	7088 per 100 000 (diagnosed patients with CKD)	Prevalence of diagnosed acute myocardial infarction in Canada, 2000–2013
Stroke prevalence	Mace-Brickman et al., 2019 [19]	4170 per 100 000 (CKD population)	9633 per 100 000 (diagnosed patients with CKD)	Of 39 120 patients in Ontario. All CKD stages and kidney failure
**China**				
CKD prevalence	Bikbov et al., 2020 [3]	7180 per 100 000 (6658–7747)	8544 per 100 000	Age-standardized rate in 2017 (95% CI)
	Wang et al., 2018 [20]	11·6%		
	Zhang et al., 2012 [21]	10 800 per 100 000		Sample population aged ≥18 years, 2009–2010
CKD prevalence change	Bikbov et al., 2020 [3]	−6·1% (−8·1 to −4·3%)	0·6% (2022–2027)	Change in age-standardized rates, 1990–2017 (95% CI)
Mortality prevalence	Bikbov et al., 2020 [3]	10.0 per 100 000 (9·2–10·4)	92 per 100 000 (diagnosed patients with CKD)	Age-standardized rate in 2017 (95% CI)
	Zhang et al., 2020 [22]	256 per 100 000		Patients in hospital with CKD
Mortality prevalence change	Bikbov et al., 2020 [3]	−19·0% (−30·2 to −13·6%)	−21·7% (2022–2027 in diagnosed patients with CKD)	Change in age-standardized rates, 1990–2017 (95% CI)

Heart failure prevalence	Zhang et al., 2020 [22]	1690 per 100 000 (CKD population)	4720 per 100 000 (diagnosed patients with CKD)	Population aged ≥18 years, 2016
Myocardial infarction prevalence	Zhang et al., 2020 [22]	1822 per 100 000 (CKD population)	13 265 per 100 000 (diagnosed patients with CKD)	2016
Stroke prevalence	Zhang et al., 2020 [22]	1322 per 100 000 (CKD population)	9018 per 100 000 (diagnosed patients with CKD)	Population aged ≥18 years
**Colombia**				
CKD prevalence	Bikbov et al., 2020 [3]	9638 per 100 000 (8935–10 505)	6679 per 100 000	Age-standardized rate in 2017 (95% CI)
CKD prevalence change	Bikbov et al., 2020 [3]	1·1% (−4·7–6·4)	14·6% (2022–2027)	Change in age-standardized rates, 1990–2017 (95% CI)
Mortality prevalence	Bikbov et al., 2020 [3]	15·7 (14·2–17·3)	99 per 100 000 (diagnosed patients with CKD)	Age-standardized rate per 100 000 (95% CI)
Mortality prevalence change	Bikbov et al., 2020 [3]	−34·1% (−40·5 to −26·9)	−3·2% (2022–2027 in diagnosed patients with CKD)	Change in age-standardized rates, 1990 and 2017 (95% CI)
Stroke prevalence	Pradilla et al., 1995 [23]	0·65% (total population)	1735 per 100 000 (diagnosed patients with CKD)	Screening 16 032 people from seven different rural and urban communities
**Denmark**				
CKD prevalence	Bikbov et al., 2020 [3]	5816 per 100 000 (5400–6285)	13 888 per 100 000	Age-standardized rate in 2017 (95% CI)
	Thomsen et al., 2018 [24]	9618 per 100 000		2000–2012
	Kampmann et al., 2021 [25]	9·93%		Large register of 669 929 individuals in southern Denmark between 2006 and 2013, providing a geographical coverage of 78%. 66 486 patients had CKD stages 3–5. Only those with a creatinine measurement were included, which could account for an overrepresentation of older/sicker patients
	Vestergaard et al., 2022 [26]	4·6–8·3%		Diagnosed adult population
CKD prevalence change	Bikbov et al., 2020 [3]	4·9% (1·8–8·1)	−2·2% (2022–2027)	Change in age-standardized rates, 1990–2017 (95% CI)
Mortality prevalence	Bikbov et al., 2020 [3]	7·7 (7·2–8·3)	320 per 100 000 (diagnosed patients with CKD)	Age-standardized rate per 100 000 (95% CI)
	Vestergaard et al., 2022 [26]	8·9%		1-year mortality
Mortality prevalence change	Bikbov et al., 2020 [3]	117·1% (101·1–135·2)	−15·3% (2022–2027 in diagnosed patients with CKD)	Change in age-standardized rates, 1990–2017 (95% CI)
Heart failure prevalence	Thomsen et al., 2018 [24]	With hyperkalaemia: 19·8%; without hyperkalaemia: 8·5% (CKD population)	12 212 per 100 000 (diagnosed patients with CKD)	
Myocardial infarction prevalence	Thomsen et al., 2018 [24]	With hyperkalaemia: 15·2%; without hyperkalaemia: 10·3% (CKD population)	10 977 per 100 000 (diagnosed patients with CKD)	

Stroke prevalence	Thomsen et al., 2018 [24]	With hyperkalaemia: 18·3%;<br>Without hyperkalaemia: 14·7%<br>(CKD population)	9984 per 100 000 (diagnosed patients with CKD)	Cerebrovascular disease prevalence
**France**				
CKD prevalence	Bikbov et al., 2020 [3]	5242 per 100 000 (4858–5697)	10 816 per 100 000	Age-standardized rate in 2017 (95% CI)
CKD prevalence change	Bikbov et al., 2020 [3]	−1·7%<br>(−6·0–2·5)	5·5% (2022–2027)	Change in age-standardized rates, 1990–2017 (95% CI)
Mortality prevalence	Bikbov et al., 2020 [3]	4·9 (4·6–5·3)	168 per 100 000 (diagnosed patients with CKD)	Age-standardized rate per 100 000 (95% CI)
Mortality prevalence change	Bikbov et al., 2020 [3]	−25·8%<br>(−31·8 to−18·7)	−1·7%<br>(2022–2027 in diagnosed patients with CKD)	Change in age-standardized rates, 1990–2017 (95% CI)
Heart failure prevalence	Villain et al., 2020 [27]	13·1%<br>(CKD population)	4712 per 100 000 (diagnosed patients with CKD)	CKD-REIN is a clinic-based, prospective cohort study of 3033 patients with CKD stages 3–4 receiving nephrologist-led care in France
Myocardial infarction prevalence	Villain et al., 2020 [27]	24·5%<br>(CKD population)	3626 per 100 000 (diagnosed patients with CKD)	Prevalence of coronary artery disease, defined as a history of angina, myocardial infarction, coronary angioplasty, or bypass. This value will be much higher than predicted for myocardial infarction alone
Stroke prevalence	Villain et al., 2020 [27]	10·0%<br>(CKD population)	6019 per 100 000 (diagnosed patients with CKD)	History of ischaemic stroke or a transient ischaemic attack
**Germany**				
CKD prevalence	Bikbov et al., 2020 [3]	5687 per 100 000	12 709 per 100 000	Age-standardized rate in 2017
	Brück et al., 2016 [28]	17·3% (16·5–18·1)		A study that investigated the prevalence of CKD stages 1–5 (95% CI) in northeast Germany
CKD prevalence change	Bommer 2002 [29]	5·4%	2·8%<br>(2022–2027)	1995–2000
Mortality prevalence	Bikbov et al., 2020 [3]	11·3 per 100 000 (10·2–12·4)	112 per 100 000 (diagnosed patients with CKD)	Age-standardized rate in 2017<br>(95% CI)
Mortality prevalence change	Bikbov et al., 2020 [3]	45·0% (30·3–61·3%)	−12·7%<br>(2022–2027 in diagnosed patients with CKD)	Change in age-standardized rates, 1990–2017 (95% CI)
Heart failure prevalence	Beck et al., 2015 [30]	24–59%<br>(CKD population)	1459 per 100 000 (diagnosed patients with CKD)	Observational prospective study with 5015 patients with CKD aged 18–74 years. Heart failure prevalence ranged from 24% in those with eGFR >90 mL/min/1·73m^2 and 59% in those with eGFR <30 mL/min/1·73m^2
Myocardial infarction prevalence	Gößwald et al., 2013 [31]	1·5–10·2%<br>(total population)	499 per 100 000 (diagnosed patients with CKD)	Results of the German Health Interview and Examination Survey for Adults (DEGS1) showed that the lifetime

				prevalence of myocardial infarction ranged from 1·5% in adults aged 40–49 years to 10·2% in adults aged 70–79 years. Sample size: 5389
Stroke prevalence (total population)	Stroke Alliance for Europe 2017 [32]	526 774 (of total population)	1035 per 100 000 (diagnosed patients with CKD)	
**Greece**				
CKD prevalence	Bikbov et al., 2020 [3]	5·34% (4·96–5·81)	11·6% of national population	Age-standardized prevalence (%), 2017 (95% CI)
CKD prevalence change	Bikbov et al., 2020 [3]	−5·3% (−9·1 to −1·1)	5·9% (2022–2027)	Change in age-standardized rates, 1990–2017 (95% CI)
Mortality prevalence	Bikbov et al., 2020 [3]	12·0 (11·2–12·9)	281 per 100 000 (diagnosed patients with CKD)	Age-standardized rate per 100 000 (95% CI)
Mortality prevalence change	Bikbov et al., 2020 [3]	−30·0% (−35·3 to−24·1)	−12·5% (2022–2027 in diagnosed patients with CKD)	Change in age-standardized rates, 1990–2017 (95% CI)
Heart failure prevalence	Calculated from Loutradis et al., 2015 [33]	17·2% (CKD population)	13 769 per 100 000 (diagnosed patients with CKD)	
Myocardial infarction prevalence		34·2% (CKD population)	12 484 per 100 000 (diagnosed patients with CKD)	
Stroke prevalence		4·7% (CKD population)	8436 per 100 000 (diagnosed patients with CKD)	
**Hungary**				
CKD prevalence	Bikbov et al., 2020 [3]	8204 per 100 000 (7596–8882)	13 629 per 100 000	Age-standardized prevalence (%), 2017 (95% CI)
CKD prevalence change	Bikbov et al., 2020 [3]	1·0% (−2·4–4·5)	−2·7% (2022–2027)	Change in age-standardized rates, 1990–2017 (95% CI)
Mortality prevalence	Bikbov et al., 2020 [3]	7·6 per 100 000 (7·2–8·1)	526 per 100 000 (diagnosed patients with CKD)	Age-standardized rate per 100 000 (95% CI)
	NHIFA, Hungarian National Statistics [34]	526·85 per 100 000		Mortality from real-world data analysis compared with the 2019 total national population
Mortality prevalence change	Bikbov et al., 2020 [3]	34·4% (26·2–44·1)	−7·1% (2022–2027 in diagnosed patients with CKD)	Change in age-standardized rates, 1990–2017 (95% CI)
**India**				
CKD prevalence	Bikbov et al., 2020 [3]	9529 per 100 000 (8852–10 280)	5456 per 100 000	Age-standardized rate in 2017 (95% CI)
	Singh et al., 2013 [35]	17·2%		In a cohort of 5588, prevalence of CKD stages 1, 2, 3, 4, and 5 was 7%, 4·3%, 4·3%, 0·8%, and 0·8%, respectively
CKD prevalence change	Bikbov et al., 2020 [3]	5·6% (3·3–8·2%)	2·5% (2022–2027)	Change in age-standardized rates, 1990–2017 (95% CI)
Mortality prevalence	Bikbov et al., 2020 [3]	22·3 per 100 000 (20·6–23·5)	92 per 100 000 (diagnosed patients with CKD)	Age-standardized rate (95% CI). Vital registration and verbal autopsy data were used to model mortality due to CKD. Data were

				standardized and mapped when each death was assigned to a single underlying cause
Mortality prevalence change	Bikbov et al., 2020 [3]	−5·5% (−14·5–2·7%)	−37·7% (2022–2027 in diagnosed patients with CKD)	Change in age-standardized rates, 1990–2017 (95% CI)
Heart failure prevalence	Huffman et al., 2010 [36]	0·3–1·75 million cases (total population)	11 446 per 100 000 (diagnosed patients with CKD)	2010
Stroke prevalence	Pandian et al., 2013 [37]	84–262 per 100 000 (rural); 424 per 100 000 (urban) (total population)	5597 per 100 000 (diagnosed patients with CKD)	Estimated adjusted prevalence in 2013
**Israel**				
CKD prevalence	Bikbov et al., 2020 [3]	6246 per 100 000 (5810–6757)	13 129 per 100 000	Age-standardized rate in 2017 (95% CI)
CKD prevalence change	Bikbov et al., 2020 [3]	0·6% (−2·1–3·3%)	−0·4% (2022–2027)	Change in age-standardized rates, 1990–2017 (95% CI)
Mortality prevalence	Bikbov et al., 2020 [3]	17·7 per 100 000 (16·5–19·0)	228 per 100 000 (diagnosed patients with CKD)	Age-standardized rate (95% CI). Vital registration and verbal autopsy data were used to model mortality due to CKD. Data were standardized and mapped when each death was assigned to a single underlying cause
Mortality prevalence change	Bikbov et al., 2020 [3]	−20·9% (−26·6 to −14·2%)	−35·6% (2022–2027 in diagnosed patients with CKD)	Change in age-standardized rates, 1990–2017 (95% CI)
Heart failure prevalence	Ministry of Health Report 2013 [38]	1–2% (total population)	3340 per 100 000 (diagnosed patients with CKD)	Local-language document
Stroke prevalence	Stroke Alliance for Europe [39]	26 450 (total population)	3073 per 100 000 (diagnosed patients with CKD)	2017
**Italy**				
CKD prevalence	Bikbov et al., 2020 [3]	5156 per 100 000 (4792–5602)	6716 per 100 000	Age-standardized rate in 2017 (95% CI)
	De Nicola et al., 2015 [40]	7·05%		2008–2012 National Health Examination Survey in Italy, population aged 35–79 years
CKD prevalence change	Capuano et al., 2012 [41]	5·9–6·2% (male); 3·9–4·5% (female)	10·2% (2022–2027)	1998–2009
	Bikbov et al., 2020 [3]	−9·2% (−12·8 to −6·1%)		Change in age-standardized rates, 1990–2017 (95% CI)
Mortality prevalence	Bikbov et al., 2020 [3]	7·3 per 100 000 (6·8–7·9)	146 per 100 000 (diagnosed patients with CKD)	Age-standardized rate in 2017 (95% CI)
Mortality prevalence change	Bikbov et al., 2020 [3]	−19·5% (−25·3 to −12·9%)	−5·4% (2022–2027 in diagnosed patients with CKD)	Change in age-standardized rates, 1990–2017 (95% CI)
	Navarra et al., 2021 [42]	60% (male); 54% (female)		Over 16 years, 2003–2019
Heart failure prevalence	Barbieri et al., 2020 [43]	12 600 per 100 000 (CKD population)	13 488 per 100 000 (diagnosed patients with CKD)	CKD stages 3a–5
Myocardial infarction prevalence	Giampaoli et al., 2015 [44]	1·6% (male); 0·6% (female) (total population)	11 697 per 100 000 (diagnosed patients with CKD)	Population aged 35–74 years, 2008–2012

Stroke prevalence	Stroke Alliance for Europe 2017 [45]	351 820<br>(total population)	8884 per 100 000<br>(diagnosed patients with CKD)	2017
**Japan**				
CKD prevalence	Bikbov et al., 2020 [3]	8404 per 100 000<br>(7838–9068)	18 787 per 100 000	Age-standardized rate in 2017 (95% CI)
	Imai et al., 2007 [46]	19·1 million cases		Population aged >18 years, CKD stages 3a–5, in 2004
CKD prevalence change	Nagata et al., 2010 [47]	13·8–22·1% (male);<br>14·3–15·3% (female)	8·5% (2022–2027)	Population aged ≥40 years, 1974–2002
	Bikbov et al., 2020 [3]	−5·9%<br>(−8·6 to −3·3%)		Change in age-standardized rates, 1990–2017 (95% CI)
	Takeuchi et al., 2021 [48]	14·1 per 100 000		2005–2017
Mortality prevalence	Bikbov et al., 2020 [3]	7·5 per 100 000 (7·1–8·0)	400 per 100 000<br>(diagnosed patients with CKD)	Age-standardized rate in 2017 (95% CI)
Mortality prevalence change	Bikbov et al., 2020 [3]	−40·3%<br>(−43·0 to−36·3%)	−2·3%<br>(2022–2027 in diagnosed patients with CKD)	Change in age-standardized rates, 1990–2017 (95% CI)
Heart failure prevalence	Tanaka et al., 2017 [49]	2764·7 per 100 000<br>(CKD population)	20 942 per 100 000<br>(diagnosed patients with CKD)	Patients with CKD, pre-dialysis
Myocardial infarction prevalence	Ninomiya et al., 2009 [50]	5546·7 per 100 000<br>(CKD population)	22 700 per 100 000<br>(diagnosed patients with CKD)	
**Mexico**				
CKD prevalence	Bikbov et al., 2020 [3]	12 107 per 100 000<br>(11 292–13 009)	10 927 per 100 000	Age-standardized rate in 2017 (95% CI)
CKD prevalence change	Bikbov et al., 2020 [3]	10·2% (7·7–13·0%)	6·3% (2022–2027)	Change in age-standardized rates, 1990–2017 (95% CI)
Mortality prevalence	Bikbov et al., 2020 [3]	58·1 per 100 000<br>(56·4–59·5)	216 per 100 000<br>(diagnosed patients with CKD)	Age-standardized rate (95% CI). Vital registration and verbal autopsy data were used to model mortality due to CKD. Data were standardized and mapped when each death was assigned to a single underlying cause
Mortality prevalence change	Bikbov et al., 2020 [3]	102·3% (94·4–108·8%)	−31·6%<br>(2022–2027 in diagnosed patients with CKD)	Change in age-standardized rates, 1990–2017 (95% CI)
**Netherlands**				
CKD prevalence	Bikbov et al., 2020 [3]	6142 per 100 000<br>(5688–6653)	12 194 per 100 000	Age-standardized rate in 2017 (95% CI)
	Sundström et al., 2022 [51]	8·9%		
CKD prevalence change	Bikbov et al., 2020 [3]	1·3%<br>(−3·1–6·1%)	5·1% (2022–2027)	Change in age-standardized rates, 1990–2017 (95% CI)
Mortality prevalence	Bikbov et al., 2020 [3]	7·1 per 100 000 (6·7–7·6)	244 per 100 000<br>(diagnosed patients with CKD)	Age-standardized rate (95% CI). Vital registration and verbal autopsy data were used to model mortality due to CKD. Data were standardized and mapped when each death was assigned to a single underlying cause

Mortality prevalence change	Bikbov et al., 2020 [3]	6·9% (−0·5–15·8%)	−11·8% (2022–2027 in diagnosed patients with CKD)	Change in age-standardized rates, 1990–2017 (95% CI)
Heart failure prevalence	Analysis from Sundström et al., 2022 [51]	15·05% (CKD population)	6946 per 100 000 (diagnosed patients with CKD)	
Myocardial infarction prevalence		27·81% (CKD population)	14 495 per 100 000 (diagnosed patients with CKD)	
Stroke prevalence		18·88% (CKD population)	8302 per 100 000 (diagnosed patients with CKD)	
**Philippines**				
CKD prevalence	Bikbov et al., 2020 [3]	11 049 per 100 000 (10 263–11 879)	5469 per 100 000	Age-standardized rate in 2017 (95% CI)
	Morales et al., 2005 [52]	2·6%		CKD stages 3a–5, National Nutrition Health Survey 2003 renal report
CKD prevalence change	Bikbov et al., 2020 [3]	13·2% (10·2–16·1%)	5·6% (2022–2027)	Change in age-standardized rates, 1990–2017 (95% CI)
Mortality prevalence	Bikbov et al., 2020 [3]	50·3 per 100 000 (44·5–56·4)	89 per 100 000 (diagnosed patients with CKD)	Age-standardized rate (95% CI). Vital registration and verbal autopsy data were used to model mortality due to CKD. Data were standardized and mapped when each death was assigned to a single underlying cause
Mortality prevalence change	Bikbov et al., 2020 [3]	108·9% (83·0–137·8%)	−21·4% (2022–2027 in diagnosed patients with CKD)	Change in age-standardized rates, 1990–2017 (95% CI)
Heart failure prevalence	Tumanan-Mendoza et al., 2017 [53]	1·6% per 100 000 (total population)	13 561 per 100 000 (diagnosed patients with CKD)	Population aged ≥19 years in 2014
Stroke prevalence	Sy et al., 2012 [54]	1·4–1·2% (total population)	9288 per 100 000 (diagnosed patients with CKD)	2003–2008
**Poland**				
CKD prevalence	Bikbov et al., 2020 [3]	7271 per 100 000 (6702–7943)	10 730 per 100 000	Age-standardized rate in 2017 (95% CI)
CKD prevalence change	Bikbov et al., 2020 [3]	−6·0% (−12·9–0·9)	5·5% (2022–2027)	Change in age-standardized rates, 1990–2017 (95% CI)
Mortality prevalence	Bikbov et al., 2020 [3]	4·8 per 100 000 (4·5–5·1)	289 per 100 000 (diagnosed patients with CKD)	Age-standardized rate (95% CI). Vital registration and verbal autopsy data were used to model mortality due to CKD. Data were standardized and mapped when each death was assigned to a single underlying cause
Mortality prevalence change	Bikbov et al., 2020 [3]	−50·3% per 100 000 (−53·5–46·2)	−18·9% (2022–2027 in diagnosed patients with CKD)	Change in age-standardized rates, 1990–2017 (95% CI)
Heart failure prevalence	Puch-Walczak et al., 2022 [55]	2·4–4·3% (total population)	14 986 per 100 000 (diagnosed patients with CKD)	From 2414 participants of NATPOL 2011, aged 18–79
Stroke prevalence	Stroke Alliance for Europe 2017 [56]	652·3 per 100 000 (total population)	46 260 per 100 000 (diagnosed patients with CKD)	

**Romania**				
CKD prevalence	Bikbov et al., 2020 [3]	8204 per 100 000 (11 292–13 009)	7015 per 100 000	Age-standardized rate in 2017 (95% CI)
CKD prevalence change	Bikbov et al., 2020 [3]	1·0% (−2·4–4·5%)	20·1% (2022–2027)	Change in age-standardized rates, 1990–2017 (95% CI)
Mortality prevalence	Bikbov et al., 2020 [3]	7·6 per 100 000 (7·2–8·1)	123 per 100 000 (diagnosed patients with CKD)	Age-standardized rate (95% CI). Vital registration and verbal autopsy data were used to model mortality due to CKD. Data were standardized and mapped when each death was assigned to a single underlying cause
Mortality prevalence change	Bikbov et al., 2020 [3]	34·4% (26·2–44·1%)	20·2% (2022–2027 in diagnosed patients with CKD)	Change in age-standardized rates, 1990–2017 (95% CI)
**Saudi Arabia**				
CKD prevalence	Bikbov et al., 2020 [3]	9892 per 100 000 (9139–10 812)	12 283 per 100 000	Age-standardized rate in 2017 (95% CI)
CKD prevalence change	Bikbov et al., 2020 [3]	0·1% (−4·0–4·4%)	12·9% (2022–2027)	Change in age-standardized rates, 1990–2017 (95% CI)
Mortality prevalence	Bikbov et al., 2020 [3]	29·9 per 100 000 (24·2–34·1)	83 per 100 000 (diagnosed patients with CKD)	Age-standardized rate (95% CI). Vital registration and verbal autopsy data were used to model mortality due to CKD. Data were standardized and mapped when each death was assigned to a single underlying cause
Mortality prevalence change	Bikbov et al., 2020 [3]	−2·6% (−21·7–22·7%)	9·2% (2022–2027 in diagnosed patients with CKD)	Change in age-standardized rates, 1990–2017 (95% CI)
Heart failure prevalence	AbuRuz et al., 2015 [57]	455 222 cases (total population)	5700 per 100 000 (diagnosed patients with CKD)	Extrapolated
Stroke prevalence	Memon et al., 2019 [58]	29 per 100 000 (total population)	1792 per 100 000 (diagnosed patients with CKD)	Although there is a lack of data on the prevalence of stroke in Saudi Arabia, a study conducted by the Ministry of Health reported 29 per 100 000 per year
**Singapore**				
CKD prevalence	Bikbov et al., 2020 [3]	9333 per 100 000 (8659–10 090)	15 892 per 100 000	Age-standardized rate in 2017 (95% CI)
CKD prevalence change	Bikbov et al., 2020 [3]	−8·8% (−10·9 to −6·6%)	8·3% (2022–2027)	Change in age-standardized rates, 1990–2017 (95% CI)
	Wong et al., 2018 [59]	12·2–24·3%		2007–2035, using a Markov model
Mortality prevalence	Bikbov et al., 2020 [3]	9·0 per 100 000 (8·4–9·7)	143 per 100 000 (diagnosed patients with CKD)	Age-standardized rate (95% CI). Vital registration and verbal autopsy data were used to model mortality due to CKD. Data were standardized and mapped when each death was assigned to a single underlying cause

Mortality prevalence change	Bikbov et al., 2020 [3]	−36·7% (−41·5 to −31·4%)	−4·5% (2022–2027 in diagnosed patients with CKD)	Change in age-standardized rates, 1990–2017 (95% CI)
Heart failure prevalence	Picco et al., 2016 [60]	2·8% (CKD population)	5424 per 100 000 (diagnosed patients with CKD)	Data analysed from the Singapore Mental Health Study (2009–2010). Sample size: 6616
Stroke prevalence	Teh et al., 2018 [61]	7·6% (CKD population)	5550 per 100 000 (diagnosed patients with CKD)	Data from the Well-being of the Singapore Elderly study, a cross-sectional epidemiological survey conducted 2012–2013
**South Korea**				
CKD prevalence	Bikbov et al., 2020 [3]	7103 per 100 000 (6560–7659)	10 808 per 100 000	Age-standardized rate in 2017 (95% CI)
CKD prevalence change	Lee et al., 2020 [62]	57·8–157·1 per 100 000	19·6% (2022–2027)	Kidney failure. Based on a retrospective population-based cohort study using the National Health Insurance Service–National Sample Cohort; 2006–2015
	Bikbov et al., 2020 [3]	−9·2% (−14·5 to −5·6%)		Change in age-standardized rates, 1990–2017 (95% CI)
Mortality prevalence	Bikbov et al., 2020 [3]	7·7 per 100 000 (7·1–8·2)	71 per 100 000 (diagnosed patients with CKD)	Age-standardized rate (95% CI). Vital registration and verbal autopsy data were used to model mortality due to CKD. Data were standardized and mapped when each death was assigned to a single underlying cause
Mortality prevalence change	Bikbov et al., 2020 [3]	−40·6% (−45·8 to −35·1%)	39·1% (2022–2027 in diagnosed patients with CKD)	Change in age-standardized rates, 1990–2017 (95% CI)
Stroke prevalence	Korean Stroke Society 2018 [63]	1·71% (total population)	7105 per 100 000 (diagnosed patients with CKD)	Population aged ≥19 years
**Spain**				
CKD prevalence	Gorostidi et al., 2018 [64]	15·1%	10 729 per 100 000	Population aged ≥18 years, 2008–2010
	Otero González et al., 2010 [65]	6·83%		Population aged ≥20 years
CKD prevalence change	Bikbov et al., 2020 [3]	−5·9% (−10·3 to −1·6%)	9·1% (2022–2027)	Change in age-standardized rates, 1990–2017 (95% CI)
Mortality prevalence	Bikbov et al., 2020 [3]	8·0 per 100 000 (7·5–8·6)	242 per 100 000 (diagnosed patients with CKD)	Age-standardized rate (95% CI). Vital registration and verbal autopsy data were used to model mortality due to CKD. Data were standardized and mapped when each death was assigned to a single underlying cause
Mortality prevalence change	Bikbov et al., 2020 [3]	−32·3% (−37·5 to −26·5%)	10·4% (2022–2027 in diagnosed patients with CKD)	Change in age-standardized rates 1990–2017 (95% CI)
Heart failure prevalence	Martínez-Castelao et al., 2011 [66]	17 500 per 100 000 (CKD population)	11 403 per 100 000 (diagnosed patients with CKD)	Population aged 19–94 years

Myocardial infarction prevalence	Martínez-Castelao et al., 2011 [66]	11 100 per 100 000 (CKD population)	5270 per 100 000 (diagnosed patients with CKD)	Population aged 19–94 years
Stroke prevalence	Stroke Alliance for Europe 2017 [67]	357·3 per 100 000 in total population	4709 per 100 000 (diagnosed patients with CKD)	2017
**Sweden**				
CKD prevalence	CELOSIA. 2016 [data on file]	5437 per 100 000	9258 per 100 000	
	CELOSIA. 2017 [data on file]	5762 per 100 000		
	Bikbov et al., 2020 [3]	6839 per 100 000 (6362–7400)		Age-standardized rate in 2017 (95% CI)
	CELOSIA. 2018 [data on file]	6154 per 100 000		
	CELOSIA. 2019 [data on file]	6220 per 100 000		
	CELOSIA. 2020 [data on file]	6209 per 100 000		
CKD prevalence change	Bikbov et al., 2020 [3]	3·0% (0·3–5·7%)	1·5% (2022–2027)	Change in age-standardized rates, 1990–2017 (95% CI)
Mortality prevalence	Bikbov et al., 2020 [3]	5·7 per 100 000 (5·3–6·1)	202 per 100 000 (diagnosed patients with CKD)	Age-standardized rate (95% CI). Vital registration and verbal autopsy data were used to model mortality due to CKD. Data were standardized and mapped when each death was assigned to a single underlying cause
Mortality prevalence change	Bikbov et al., 2020 [3]	57·5% (46·9–68·8%)	−18·9% (2022–2027 in diagnosed patients with CKD)	Change in age-standardized rates, 1990–2017 (95% CI)
Heart failure prevalence	Lindmark et al., 2019 [68]	2·18% (total population)	11 887 per 100 000 (diagnosed patients with CKD)	Electronic medical records 2014. Primary and secondary care patients from Uppsala and Västerbotten
Stroke prevalence	Sweden stroke statistics 2022 [69]	340 per 100 000 (total population)	8356 per 100 000 (diagnosed patients with CKD)	2020
**Taiwan**				
CKD prevalence	Bikbov et al., 2020 [3]	8145 per 100 000 (7557–8770)	10 620 per 100 000	Age-standardized rate in 2017 (95% CI)
CKD prevalence change	Tsai et al., 2007 [70]	1·99–9·83%	17·1% (2022–2027)	Prospective cohort study of 106 094 individuals aged ≥20 years, 1996–2003
	Bikbov et al., 2020 [3]	−4·9% (−8·0 to −1·7%)		Change in age-standardized rates, 1990–2017 (95% CI)
Mortality prevalence	Bikbov et al., 2020 [3]	17·4 per 100 000 (6·3–18·5)	90 per 100 000 (diagnosed patients with CKD)	Age-standardized rate (95% CI). Vital registration and verbal autopsy data were used to model mortality due to CKD. Data were standardized and mapped when each death was assigned to a single underlying cause
Mortality prevalence change	Bikbov et al., 2020 [3]	−15·5% (−1·9 to −8·8%)	28·8% (2022–2027 in diagnosed patients with CKD)	Change in age-standardized rates, 1990–2017 (95% CI)

Heart failure prevalence	Lee et al., 2018 [71]	0·6%<br>(CKD population)	10 648 per 100 000<br>(diagnosed patients with CKD)	The prevalence of heart failure in CKD was 0·4% for stage 3, 1·1% for stage 4, and 0·6% for stage 5. Sample: 1463 patients with CKD stages 3–5, ≥18 years of age
Stroke prevalence	Huang et al., 1997 [72]	595 per 100 000<br>(total population)	2858 per 100 000<br>(diagnosed patients with CKD)	11 925 individuals were interviewed, and 71 of them had experienced stroke. The crude point prevalence was 5·95 per 1000
**Thailand**				
CKD prevalence	Bikbov et al., 2020 [3]	10 292 per 100 000<br>(9461–11 174)	17 465 per 100 000	Age-standardized rate in 2017 (95% CI)
CKD prevalence change	Bikbov et al., 2020 [3]	−3·9%<br>(−7·2 to −0·3%)	8·4% (2022–2027)	Change in age-standardized rates, 1990–2017 (95% CI)
Mortality prevalence	Bikbov et al., 2020 [3]	23·3 per 100 000<br>(20·5–25·9)	203 per 100 000<br>(diagnosed patients with CKD)	Age-standardized rate (95% CI). Vital registration and verbal autopsy data were used to model mortality due to CKD. Data were standardized and mapped when each death was assigned to a single underlying cause
Mortality prevalence change	Bikbov et al., 2020 [3]	−12·6%<br>(−23·6 to −0·7%)	14·0%<br>(2022–2027 in diagnosed patients with CKD)	Change in age-standardized rates, 1990–2017 (95% CI)
Heart failure prevalence	Reyes et al., 2016 [73]	0·4%<br>(total population)	5757 per 100 000<br>(diagnosed patients with CKD)	
Myocardial infarction prevalence	Sakboonyarat et al., 2018 [74]	3·54%<br>(total population)	16 061 per 100 000<br>(diagnosed patients with CKD)	Patients with diabetes (25 902 patients; >18 years, enrolled 2012–2013)
Stroke prevalence	Suwanwela 2014 [75]	1·88%<br>(total population)	6778 per 100 000<br>(diagnosed patients with CKD)	Based on the Thai Epidemiological Stroke Study. Population aged ≥45 years
**Türkiye**				
CKD prevalence	Bikbov et al., 2020 [3]	10 311 per 100 000<br>(9532–11 248)	11 742 per 100 000	Age-standardized rate in 2017 (95% CI)
	Süleymanlar et al., 2011 [76]	15·7%		Percentage prevalence, 2011
CKD prevalence change	Bikbov et al., 2020 [3]	−2·5%<br>(−7·4–2·6%)	4·5% (2022–2027)	Change in age-standardized rates, 1990–2017 (95% CI)
Mortality prevalence	Bikbov et al., 2020 [3]	17·8 per 100 000<br>(15·8–19·7)	188 per 100 000<br>(diagnosed patients with CKD)	Age-standardized rate (95% CI). Vital registration and verbal autopsy data were used to model mortality due to CKD. Data were standardized and mapped when each death was assigned to a single underlying cause
Mortality prevalence change	Bikbov et al., 2020 [3]	−32·0%<br>(−40·4 to −21·7%)	–25·1%<br>(2022–2027 in diagnosed patients with CKD)	Change in age-standardized rates, 1990–2017 (95% CI)
Heart failure prevalence	Değertekin et al., 2012 [77]	2·9%<br>(total population)	5672 per 100 000<br>(diagnosed patients with CKD)	4650 randomly selected individuals, aged ≥35 years

Myocardial infarction prevalence	Kılıç et al., 2020 [78]	6·7%<br>(total population)	2208 per 100 000<br>(diagnosed patients with CKD)	Patients diagnosed with myocardial infarction in 2018. Sample size: 1793
Stroke prevalence	Börü et al., 2018 [79]	1·7%<br>(total population)	2408 per 100 000<br>(diagnosed patients with CKD)	Population aged ≥18 years, 2013. Sample size: 3320
**UAE**				
CKD prevalence	Xie et al., 2018 [80]	3397 per 100 000<br>(9532–11 248)	11 345 per 100 000	Prevalence of CKD per 100 000, 2016 (95% CI)
	Bikbov et al., 2020 [3]	9951 per 100 000<br>(9198–10 828)		Age-standardized rate in 2017 (95% CI)
CKD prevalence change	Bikbov et al., 2020 [3]	−4·0%<br>(−7·7–0·1%)	13·7% (2022–2027)	Change in age-standardized rates, 1990–2017 (95% CI)
Mortality prevalence	Bikbov et al., 2020 [3]	30·8 per 100 000<br>(25·3–36·7)	55 per 100 000<br>(diagnosed patients with CKD)	Age-standardized rate (95% CI). Vital registration and verbal autopsy data were used to model mortality due to CKD. Data were standardized and mapped when each death was assigned to a single underlying cause
Mortality prevalence change	Bikbov et al., 2020 [3]	3·0%<br>(−18·1–29·0%)	41.0%<br>(2022–2027 in diagnosed patients with CKD)	Change in age-standardized rates, 1990–2017 (95% CI)
**UK**				
CKD prevalence	Bikbov et al., 2020 [3]	5167 per 100 000<br>(4819–5589)	13 377 per 100 000	Age-standardized rates in 2017 (95% CI)
	Public Health England 2014 [81]	2·6 million cases (2·3–3·0 million)		Living with CKD stages 3–5 (95% CI) (diagnosed and undiagnosed)
CKD prevalence change	Bikbov et al., 2020 [3]	−11·4%<br>(−13·7 to −8·9%)	−0·7% (2022–2027)	Change in age-standardized rates, 1990–2017 (95% CI)
	Public Health England 2014 [81]	8·3%		CKD stages 3–5, aged ≥16 years, 2011–2036
	Hounkpatin et al., 2020 [82]	12·6% (2009)– 13·9% (2016)		17 663 pooled individuals (aged ≥16) in the Health Survey for England 2003, 2009/2010 combined and 2016
Mortality prevalence	Bikbov et al., 2020 [3]	4·5 per 100 000 (4·4–4·6)	320 per 100 000<br>(diagnosed patients with CKD)	Age-standardized rate (95% CI). Vital registration and verbal autopsy data were used to model mortality due to CKD. Data were standardized and mapped when each death was assigned to a single underlying cause
Mortality prevalence change	Bikbov et al., 2020 [3]	−14·5%<br>(−16·3 to −12·6%)	−18·4%<br>(2022–2027 in diagnosed patients with CKD)	Change in age-standardized rates, 1990–2017 (95% CI)
Heart failure prevalence	British Heart Foundation [83]	>1% depending on age<br>(total population)	5080 per 100 000<br>(diagnosed patients with CKD)	Prevalence increased with age, with approximately 1% of people aged ≤65 years affected. This increased to 6–7% for those aged 75–84 years, and 12–22% for those aged ≥85 years

Myocardial infarction prevalence	Jayaraj et al., 2018 [84]	915 000 cases (total population)	4493 per 100 000 (diagnosed patients with CKD)	Self-reported national survey, 2014
Stroke prevalence	Health Survey for England 2018 [85]	2% (aged 45–54 years); 9% (aged ≥75 years) (total population)	8478 per 100 000 (diagnosed patients with CKD)	2017
**USA**				
CKD prevalence	Bikbov et al., 2020 [3]	8144 per 100 000 (7615–8783)	14 892 per 100 000	Age-standardized rate in 2017 (95% CI)
	United States Renal Data System 2018 [86]	14·8% per 100 000 (13·6–16·0%)		Among National Health and Nutrition Examination Survey participants aged ≥20 years, 2011–2014 (95% CI)
CKD prevalence change	Bikbov et al., 2020 [3]	100 per 100 000 (−3·6–8·8%)	1·4% (2022–2027)	Change in age-standardized rates, 1990–2017 (95% CI)
	National Institute of Diabetes and Digestive and Kidney Diseases 2015 [87]	2000 per 100 000		CKD increased from 12·0–14·0% between 1988 and 1994, and from 1999 to 2004, but has remained stable since 2004. The largest increase occurred in people with CKD stage 3, from 4·5–6·0%, since 1988
Mortality prevalence	Bikbov et al., 2020 [3]	14·6 per 100 000 (14·3–14·9)	237 per 100 000 (diagnosed patients with CKD)	Age-standardized rate (95% CI). Vital registration and verbal autopsy data were used to model mortality due to CKD. Data were standardized and mapped when each death was assigned to a single underlying cause
Mortality prevalence change	Bikbov et al., 2020 [3]	63 000 per 100 000	−7·7% (2022–2027 in diagnosed patients with CKD)	Change in age-standardized rates, 1990–2017
	Bowe et al., 2018 [88]	58%		2002–2016
Heart failure prevalence	Cheung et al., 2004 [89]	40 000 per 100 000 (kidney failure population)	13 315 per 100 000 (diagnosed patients with CKD)	40% of 1846 patients undergoing haemodialysis, 1995–2000
	Komanduri et al., 2017 [90]	>5·8 million cases (total population)		
Myocardial infarction prevalence	Bradley et al., 2019 [91]	427 per 100 000 (total population)	6527 per 100 000 (diagnosed patients with CKD)	Cohort study of 1·3 million patients hospitalized in US Veterans' Health Administration facilities, 2007–2007
Stroke prevalence	Benjamin et al., 2018 [92]	2·7% (1·9–4·3%) (total population)	13 148 per 100 000 (diagnosed patients with CKD)	Incidence of stroke ranges from 1.9% in Minnesota to 4.3% in Alabama

CI=confidence interval. CKD=chronic kidney disease. eGFR=estimated glomerular filtration rate. NATPOL=A National, Cross-sectional Observational study conducted in Poland. NHIFA=National Health Insurance Fund Administration. REIN=Renal Epidemiology and Information Network. WHO=World Health Organization.

Table S4: Baseline percentage of drivers of CKD, and country/region income categories in 2022

Country/region	Population aged ≥ 65 (%)	Hypertension (%)	Coronary heart disease (%)	Stroke (%)	Diabetes (%)	Smoking (%)	Obesity (%)	Income category
**Americas**								
Brazil	9.6	23.3	1.9	1.4	10.1	16.5	22.1	Middle
Canada	18.1	13.2	3.3	1.8	8.4	17.5	29.4	High
Colombia	9.1	19.2	2.1	1.0	9.8	7.9	22.3	Middle
Mexico	7.6	19.7	2.0	1.1	15.6	13.9	28.9	Middle
USA	16.6	12.9	2.9	2.3	9.4	25.1	36.2	High
**Europe**								
Belgium	19.3	17.5	3.8	1.3	4.8	25.0	22.1	High
Denmark	20.2	20.6	3.6	1.3	5.6	18.6	19.7	High
France	20.8	22.0	3.4	1.2	5.4	34.6	21.6	High
Germany	21.7	19.9	4.5	1.6	5.3	28.0	22.3	High
Greece	22.3	19.1	3.7	1.6	5.1	39.1	24.9	High
Hungary	20.2	30.0	6.9	2.3	6.0	30.6	26.4	High
Italy	23.3	21.2	4.7	1.3	5.1	23.4	19.9	High
Netherlands	20.0	18.7	4.6	1.3	5.3	23.4	20.4	High
Poland	18.7	28.7	4.1	1.7	9.0	26.0	23.1	High
Romania	19.2	30.6	5.8	2.4	7.7	25.5	22.5	Middle
Spain	20.0	19.2	3.5	1.3	6.3	27.9	23.8	High
Sweden	20.3	19.3	3.8	1.8	4.2	28.8	20.6	High
Türkiye	9.0	20.3	3.6	1.4	7.9	29.3	32.1	Middle
UK	18.7	15.2	3.1	1.1	5.2	19.2	27.8	High
**Asia-Pacific/Middle East**								
Australia	16.2	15.2	5.3	1.1	6.6	16.2	29.0	High
China	12.0	19.2	3.3	2.1	8.8	24.7	6.2	Middle
India	6.6	25.8	2.8	0.7	9.0	27.0	3.9	Middle
Israel	12.4	16.6	2.2	0.8	7.4	25.5	26.1	High
Japan	28.4	17.6	3.0	3.3	7.7	21.9	4.3	High
Philippines	5.5	22.6	1.7	1.2	9.7	24.3	6.4	Middle
Saudi Arabia	3.5	23.3	2.4	1.4	19.6	16.6	35.4	High
Singapore	13.4	14.6	2.0	1.5	9.5	16.5	6.1	High
South Korea	15.8	11.0	1.6	2.0	7.5	22.0	4.7	High
Taiwan	16.1	25.0	3.7	1.8	8.2	19.4	22.8	High
Thailand	13.0	22.3	2.3	1.7	7.5	22.8	10.0	High
UAE	1.3	21.1	2.2	1.5	18.8	18.2	31.7	High
**Mean**								
High income	17.4	19.3	3.5	1.6	7.7	23.8	22.1	N/A
Middle income	9.8	22.6	2.9	1.4	9.8	21.1	18.1	N/A

Table S5: Overall prevalence of CKD in 2022 and 2027 expressed as the total number of cases

CKD prevalence – total cases (including KRT)									
**Year**	**Country/region**	**CKD stage**						**Total**	**% change 2022–2027***
		**1**	**2**	**3a**	**3b**	**4**	**5**		
**Americas**									
2022	Brazil	3 382 458	3 730 243	10 114 500	3 335 817	336 203	45 277	**20 944 499**	12.5
2027		3 843 453	3 491 389	12 645 280	3 014 572	435 512	126 639	**23 556 846**	
2022	Canada	1 188 666	919 125	2 146 775	716 166	134 254	17 720	**5 122 707**	6.4
2027		1 341 739	877 337	2 435 597	650 685	110 591	35 199	**5 451 149**	
2022	Colombia	898 101	612 521	1 601 180	273 497	49 124	6098	**3 440 522**	16.9
2027		1 022 697	571 779	2 142 613	227 197	39 934	17 004	**4 021 223**	
2022	Mexico	5 861 831	4 199 844	2 944 663	956 664	233 149	180 129	**14 376 281**	11.2
2027		6 797 431	3 705 284	4 314 933	868 726	209 321	89 987	**15 985 683**	
2022	USA	17 835 056	12 754 591	14 432 472	4 407 547	370 093	58 447	**49 858 206**	4.2
2027		19 884 476	11 247 883	16 062 028	3 977 608	623 253	171 521	**51 966 771**	
**Europe**									
2022	Belgium	347 131	281 795	659 476	257 066	94 542	15 243	**1 655 253**	0.3
2027		386 204	256 252	732 369	211 885	54 787	19 383	**1 660 881**	
2022	Denmark	177 810	144 484	353 422	113 087	19 365	2166	**810 335**	−0.4
2027		200 810	131 127	380 577	79 691	11 074	4107	**807 387**	
2022	France	2 350 210	1 525 888	2215 308	713 743	213 930	744 85	**7 093 565**	6.7
2027		2 631 722	1 283 087	2 802 207	590 843	191 843	69 063	**7 568 766**	
2022	Germany	6 984 518	1 373 921	1 684 581	511 074	94 390	12 145	**10 660 629**	2.1
2027		6 723 994	1 378 665	2 248 226	436 279	82 330	19 589	**10 889 083**	
2022	Greece	167 839	204 579	651 662	141 254	26 169	3326	**1 194 829**	3.2
2027		188 258	179 969	734 772	108 841	15 536	6024	**1 233 401**	
2022	Hungary	298 411	240 395	571 230	170 340	26 028	2830	**1 309 234**	−4.3
2027		321 699	206 770	601 055	105 004	12 603	5286	**1 252 417**	
2022	Italy	1 242 316	772 726	1 485 120	379 249	111 634	56 061	**4 047 106**	8.9
2027		1 317 372	631 008	1 977 604	363 478	78 897	38 510	**4 406 869**	
2022	Netherlands	531 041	477 658	803 327	239 544	42 378	4895	**2 098 843**	6.2
2027		593 339	432 433	993 931	172 299	27 453	8675	**2 228 130**	
2022	Poland	773 400	960 191	1 724 862	500 306	81 463	9239	**4 049 462**	4.3
2027		916 412	882 365	2 038 342	325 147	45 193	15 643	**4 223 103**	
2022	Romania	263 841	232 391	712 736	112 138	12 843	1188	**1 335 136**	17.3
2027		285 658	179 489	1 000 945	80 289	12 284	7321	**1 565 985**	
2022	Spain	791 444	872 500	2 705 692	631 210	117 072	17 651	**5 135 568**	10.6
2027		893 732	783 582	3 234 633	640 025	93 593	33 298	**5 678 863**	
2022	Sweden	233 764	196 392	358 195	112 621	28 219	16 901	**946 092**	4.2
2027		270 340	167 666	437 067	79 490	18 123	13 196	**985 882**	
2022	Türkiye	4 555 662	2 329 183	1 696 844	1 199 880	200 452	64 300	**10 046 321**	7.0

2027		5 131 344	2 151 851	2 464 412	772 707	190 560	42 668	**10 753 542**	
2022	UK	2 055 460	1 594 353	3 887 259	1 343 103	249 920	33 011	**9 163 106**	1.1
2027		2 277 613	1 451 597	4 178 943	1 127 044	179 468	52 407	**9 267 073**	
**Asia-Pacific/Middle East**									
2022	Australia	919 290	578 290	633 818	141 386	36 338	15 523	**2 324 645**	12.1
2027		1 048 919	515 238	847 898	150 768	30 460	12 823	**2 606 106**	
2022	China	88 782 770	13 966 257	16 982 472	3 070 155	725 812	233 376	**123 760 844**	1.5
2027		90 928 832	13 292 506	17 982 359	2 973 833	374 547	123 486	**125 675 563**	
2022	India	49 806 047	9 834 515	11 834 383	3 930 004	673 006	671 075	**76 749 030**	7.0
2027		54 000 875	9 218 592	15 936 886	2 493 412	383 447	122 190	**82 155 403**	
2022	Israel	641 816	219 959	200 088	71 511	32 146	5984	**1 171 505**	7.0
2027		715 533	216 512	243 950	54 732	16 850	5729	**1 253 305**	
2022	Japan	2 737 632	7 944 141	6 823 351	5 411 187	631 876	45 341	**23 593 527**	6.1
2027		3 146 006	6 860 559	10 610 404	3 480 895	811 396	118 604	**25 027 864**	
2022	Philippines	2 119 469	1 045 760	2 422 282	427 649	103 349	34 235	**6 152 744**	12.3
2027		2 411 704	955 075	2 967 006	483 746	62 990	26 290	**6 906 811**	
2022	Saudi Arabia	1 481 681	941 397	1 382 497	369 203	121 747	106 321	**4 402 846**	20.1
2027		1 755 905	925 010	1 866 359	455 998	142 236	141 487	**5 286 995**	
2022	Singapore	348 517	228 422	290 509	55 905	15 879	5308	**944 539**	12.3
2027		386 639	229 016	351 054	74 639	13 228	5838	**1 060 413**	
2022	South Korea	2 356 592	1 450 976	1 326 627	319 296	83 251	11 202	**5 547 944**	19.5
2027		2 532 936	1 316 288	2 269 665	367 289	113 378	32 891	**6 632 448**	
2022	Taiwan	1 084 714	657 427	606 602	146 133	37 125	4996	**2 536 997**	17.7
2027		1 155 246	580 804	1 026 249	161 665	47 940	13 531	**2 985 437**	
2022	Thailand	1 701 041	2 495 584	6 055 902	1 569 747	272 185	144 565	**12 239 024**	8.9
2027		1 952 640	2 264 514	6 672 540	1 898 435	350 670	189 518	**13 328 317**	
2022	UAE (Emirati)	63 171	40 200	56 692	13 071	4997	3933	**182 065**	23.9
2027		76 516	45 733	80 043	13 566	5847	3961	**225 665**	
2022	UAE (Expatriate)	357 688	30 190	25 460	3392	469	561	**417 760**	9.5
2027		373 320	30 923	48 924	3178	830	252	**457 427**	

CKD prevalence per 100 000 (including KRT)

Year	Country/region	CKD stage						TOTAL	% change 2022–2027*
		1	2	3a	3b	4	5		
**Americas**									
2022	Brazil	1571	1732	4697	1549	156	21	**9726**	9.5
2027		1738	1579	5718	1363	197	57	**10 652**	
2022	Canada	3096	2394	5592	1866	350	46	**13 344**	2.3
2027		3359	2197	6098	1629	277	88	**13 649**	
2022	Colombia	1743	1189	3108	531	95	12	**6679**	14.6
2027		1947	1088	4079	433	76	32	**7655**	
2022	Mexico	4456	3192	2238	727	177	137	**10 927**	6.3
2027		4940	2693	3136	631	152	65	**11 617**	
2022	USA	5327	3810	4311	1316	111	17	**14 892**	1.4
2027		5779	3269	4668	1156	181	50	**15 102**	
**Europe**									

2022	Belgium	2975	2415	5652	2203	810	131	**14 186**	−0.9
2027		3268	2168	6198	1793	464	164	**14 055**	
2022	Denmark	3047	2476	6057	1938	332	37	**13 888**	−2.2
2027		3379	2207	6404	1341	186	69	**13 586**	
2022	France	3583	2327	3378	1088	326	114	**10 816**	5.5
2027		3968	1935	4225	891	289	104	**11 412**	
2022	Germany	8326	1638	2008	609	113	14	**12 709**	2.8
2027		8068	1654	2697	523	99	24	**13 065**	
2022	Greece	1627	1983	6317	1369	254	32	**11 582**	5.9
2027		1872	1790	7308	1082	155	60	**12 267**	
2022	Hungary	3106	2502	5946	1773	271	29	**13 629**	−2.7
2027		3405	2189	6362	1111	133	56	**13 256**	
2022	Italy	2061	1282	2464	629	185	93	**6716**	10.2
2027		2212	1060	3321	610	132	65	**7400**	
2022	Netherlands	3085	2775	4667	1392	246	28	**12 194**	5.1
2027		3414	2488	5719	991	158	50	**12 820**	
2022	Poland	2049	2544	4570	1326	216	24	**10 730**	5.5
2027		2456	2365	5463	871	121	42	**11 317**	
2022	Romania	1386	1221	3745	589	67	6	**7016**	20.1
2027		1537	965	5384	432	66	39	**8424**	
2022	Spain	1653	1823	5653	1319	245	37	**10729**	9.1
2027		1843	1616	6670	1320	193	69	**11 711**	
2022	Sweden	2288	1922	3505	1102	276	165	**9258**	1.5
2027		2578	1599	4167	758	173	126	**9400**	
2022	Türkiye	5324	2722	1983	1402	234	75	**11 742**	4.5
2027		5857	2456	2813	882	218	49	**12 274**	
2022	UK	3001	2328	5675	1961	365	48	**13 377**	−0.7
2027		3264	2080	5989	1615	257	75	**13 282**	
**Asia-Pacific/Middle East**									
2022	Australia	3526	2218	2431	542	139	60	**8917**	6.6
2027		3827	1880	3094	550	111	47	**9509**	
2022	China	6129	964	1172	212	50	16	**8544**	0.6
2027		6220	909	1230	203	26	8	**8597**	
2022	India	3541	699	841	279	48	48	**5456**	2.5
2027		3675	627	1085	170	26	8	**5591**	
2022	Israel	7193	2465	2242	801	360	67	**13 129**	−0.4
2027		7467	2259	2546	571	176	60	**13 079**	
2022	Japan	2180	6326	5433	4309	503	36	**18 787**	8.5
2027		2563	5589	8644	2836	661	97	**20 388**	
2022	Philippines	1884	929	2153	380	92	30	**5469**	5.6
2027		2016	798	2480	404	53	22	**5773**	
2022	Saudi Arabia	4134	2626	3857	1030	340	297	**12 283**	12.9
2027		4607	2427	4897	1196	373	371	**13 871**	
2022	Singapore	5864	3843	4888	941	267	89	**15 892**	8.3
2027		6278	3719	5700	1212	215	95	**17 219**	
2022	South Korea	4591	2827	2585	622	162	22	**10 808**	19.6

2027		4938	2566	4425	716	221	64	**12 930**	
2022	Taiwan	4541	2752	2539	612	155	21	**10 620**	17.1
2027		4814	2420	4276	674	200	56	**12 441**	
2022	Thailand	2427	3561	8642	2240	388	206	**17 465**	8.4
2027		2774	3217	9479	2697	498	269	**18 934**	
2022	UAE (Emirati)	3937	2505	3533	815	311	245	**11 345**	13.7
2027		4373	2614	4575	775	334	226	**12 898**	
2022	UAE (Expatriate)	4391	371	313	42	6	7	**5128**	6.7
2027		4467	370	585	38	10	3	**5473**	

CKD=chronic kidney disease. KRT=kidney replacement therapy.

Table S6: KRT prevalence per 100 000 (given for all modalities of treatment) in 2022 and 2027

KRT prevalence per 100 000						
Year	Country/region	KRT modality			TOTAL	% change 2022–2027*
		Haemodialysis	Peritoneal dialysis	Kidney transplantation		
**Americas**						
2022	Brazil	67	5	25	**97**	22.2
2027		85	8	26	**119**	
2022	Canada	49	13	50	**112**	7.5
2027		56	15	49	**120**	
2022	Colombia	50	18	17	**85**	12.9
2027		56	22	19	**96**	
2022	Mexico	68	47	72	**187**	6.1
2027		80	47	71	**198**	
2022	USA	153	18	74	**245**	1.5
2027		155	19	74	**248**	
**Europe**						
2022	Belgium	68	5	63	**137**	10.7
2027		76	6	70	**151**	
2022	Denmark	37	9	55	**101**	15.6
2027		45	13	59	**117**	
2022	France	83	5	67	**155**	22.3
2027		106	7	77	**190**	
2022	Germany	75	4	30	**108**	4.8
2027		79	4	31	**114**	
2022	Greece	112	6	34	**152**	15.2
2027		133	7	35	**175**	
2022	Hungary	79	12	120	**211**	12.0
2027		84	13	139	**236**	
2022	Italy	71	9	53	**132**	5.2
2027		73	10	56	**139**	
2022	Netherlands	30	6	76	**112**	5.0
2027		34	8	76	**118**	
2022	Poland	78	3	34	**115**	22.3
2027		98	5	38	**140**	
2022	Romania	74	2	12	**88**	27.5
2027		90	2	20	**112**	
2022	Spain	53	7	68	**127**	13.2
2027		62	9	73	**144**	
2022	Sweden	30	8	65	**104**	11.5
2027		33	9	73	**116**	
2022	Türkiye	69	3	29	**102**	13.2
2027		82	5	29	**116**	
2022	UK	38	4	62	**104**	7.7

2027		44	7	62	**112**	
**Asia-Pacific/Middle East**						
2022	Australia	45	10	51	**106**	3.3
2027		48	11	50	**110**	
2022	China	49	5	9	**62**	4.9
2027		51	5	9	**65**	
2022	India	14	1	9	**24**	−2.7
2027		13	1	9	**23**	
2022	Israel	70	6	7	**84**	1.8
2027		70	6	9	**85**	
2022	Japan	272	8	10	**290**	10.6
2027		301	9	12	**321**	
2022	Philippines	50	2	1	**52**	8.1
2027		54	2	1	**57**	
2022	Saudi Arabia	73	7	30	**110**	77.4
2027		111	13	72	**196**	
2022	Singapore	122	21	30	**173**	17.3
2027		143	27	33	**203**	
2022	South Korea	180	11	44	**236**	13.8
2027		207	12	49	**268**	
2022	Taiwan	358	31	16	**405**	6.6
2027		381	34	17	**432**	
2022	Thailand	234	61	14	**309**	27.2
2027		295	75	23	**393**	
2022	UAE (Emirati)	154	12	43	**209**	19.6
2027		170	12	67	**250**	
2022	UAE (Expatriate)	1	0	0	**1**	75.3
2027		2	0	0	**2**	

KRT=kidney replacement therapy. Note: individual values have been rounded to whole numbers. Slight discrepancies may occur between the total sum reported as a result.

Supplementary data appendix 1: sensitivity analysis on diagnosis rates

Australia: Prevalence of CKD following sensitivity analysis on diagnosis rates in which a 10% increase and decrease was applied to the diagnosis rate. Breakdowns by total, undiagnosed and diagnosed CKD are provided. The error bars represent the ±10% difference in diagnosis rate.

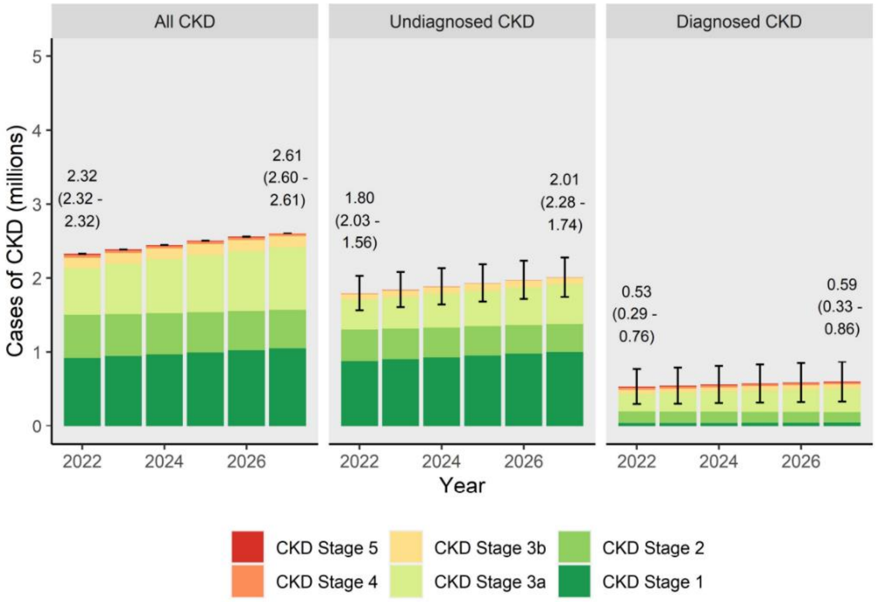

Belgium: Prevalence of CKD following sensitivity analysis on diagnosis rates in which a 10% increase and decrease was applied to the diagnosis rate. Breakdowns by total, undiagnosed and diagnosed CKD are provided. The error bars represent the ±10% difference in diagnosis rate.

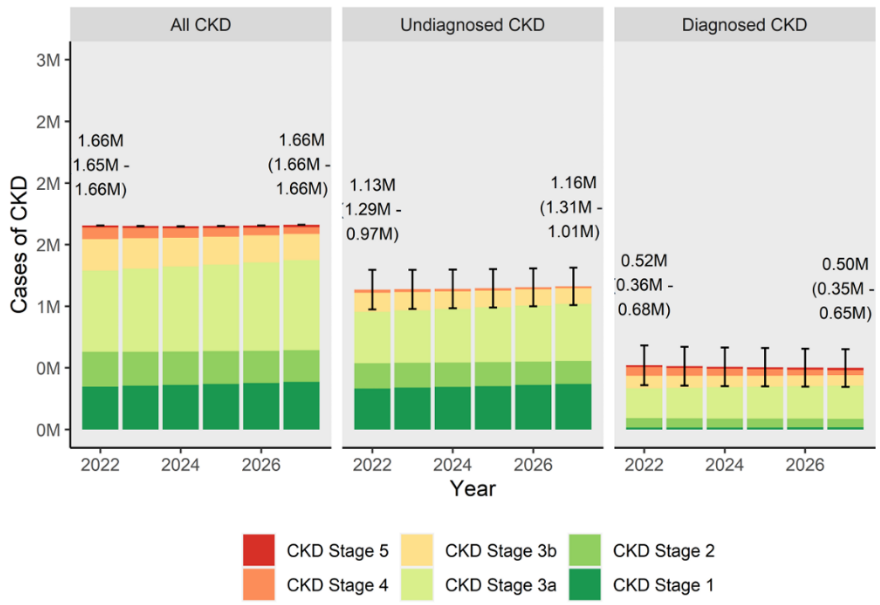

Brazil: Prevalence of CKD following sensitivity analysis on diagnosis rates in which a 10% increase and decrease was applied to the diagnosis rate. Breakdowns by total, undiagnosed and diagnosed CKD are provided. The error bars represent the ±10% difference in diagnosis rate.

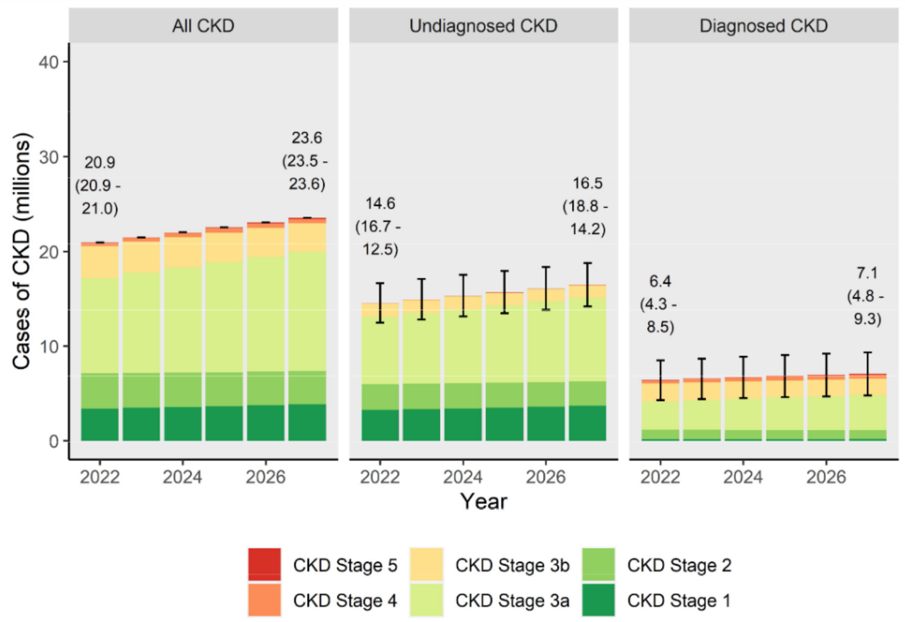

Canada: Prevalence of CKD following sensitivity analysis on diagnosis rates in which a 10% increase and decrease was applied to the diagnosis rate. Breakdowns by total, undiagnosed and diagnosed CKD are provided. The error bars represent the ±10% difference in diagnosis rate.

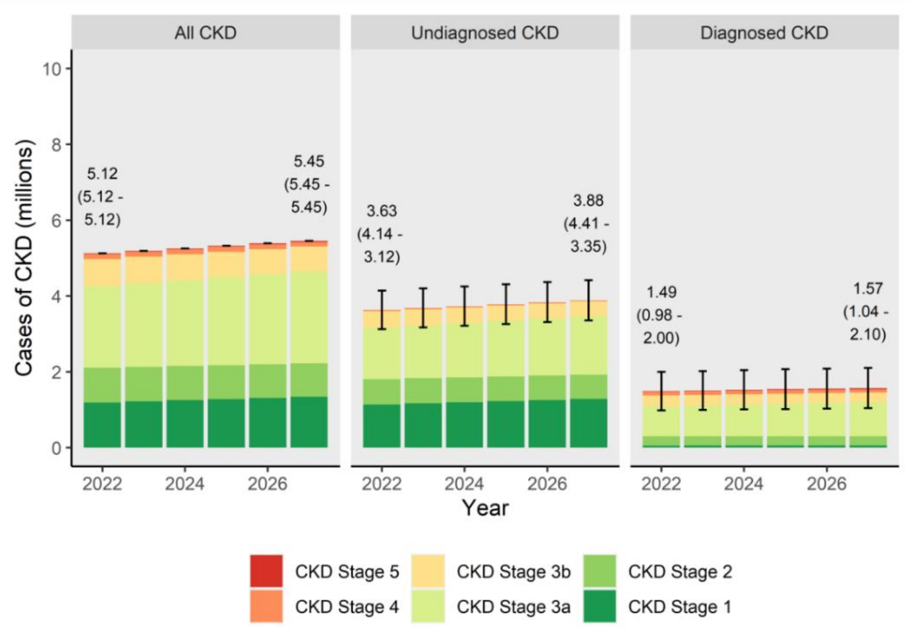

China: Prevalence of CKD following sensitivity analysis on diagnosis rates in which a 10% increase and decrease was applied to the diagnosis rate. Breakdowns by total, undiagnosed and diagnosed CKD are provided. The error bars represent the ±10% difference in diagnosis rate.

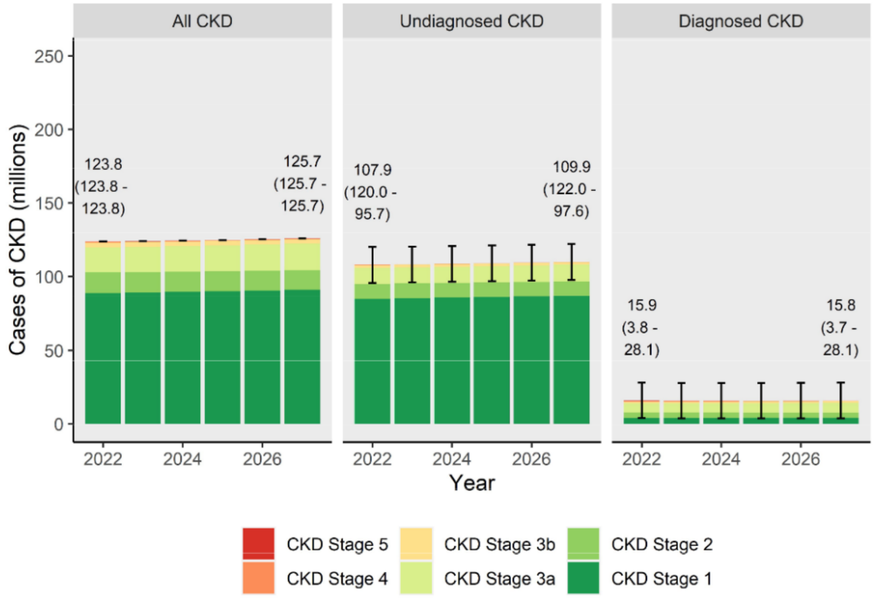

Colombia: Prevalence of CKD following sensitivity analysis on diagnosis rates in which a 10% increase and decrease was applied to the diagnosis rate. Breakdowns by total, undiagnosed and diagnosed CKD are provided. The error bars represent the ±10% difference in diagnosis rate.

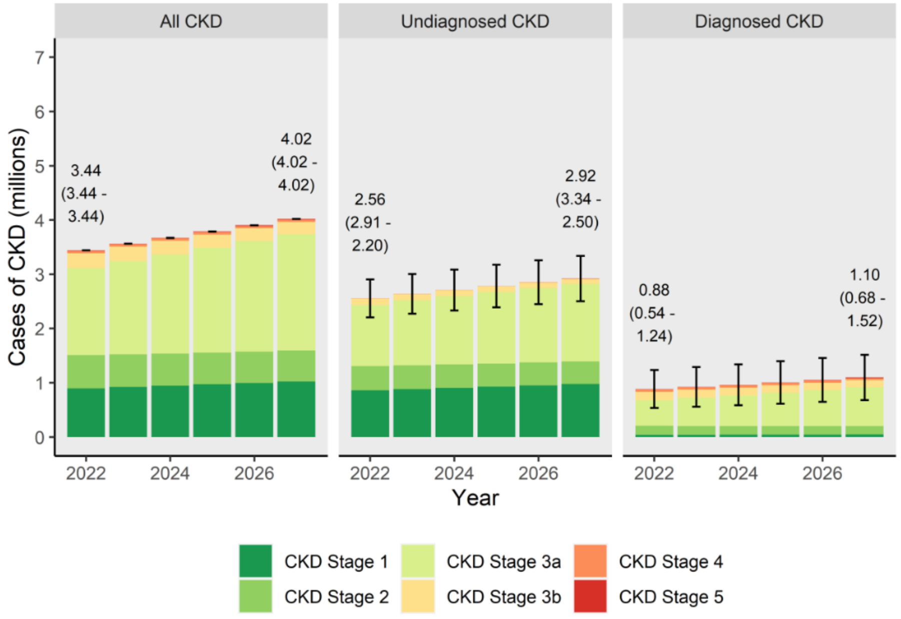

Denmark: Prevalence of CKD following sensitivity analysis on diagnosis rates in which a 10% increase and decrease was applied to the diagnosis rate. Breakdowns by total, undiagnosed and diagnosed CKD are provided. The error bars represent the ±10% difference in diagnosis rate.

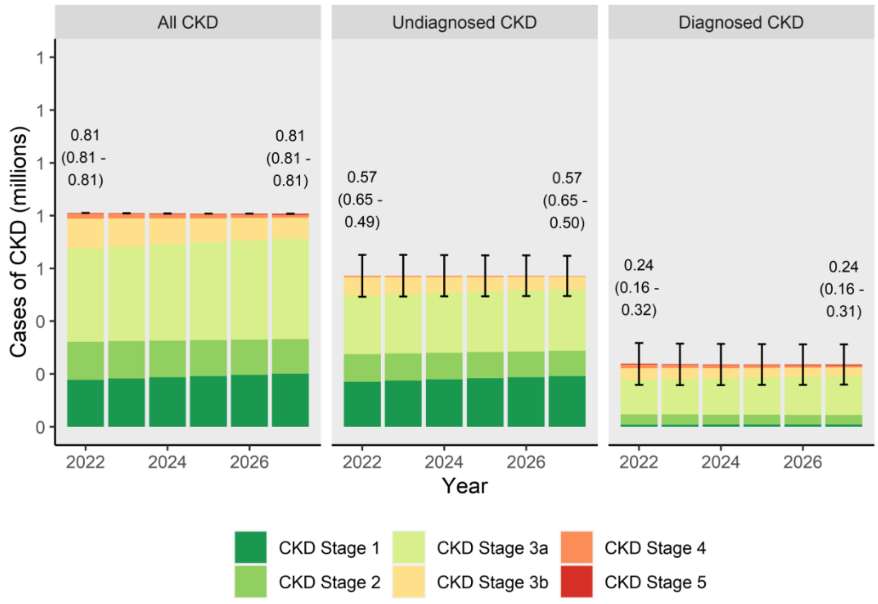

France: Prevalence of CKD following sensitivity analysis on diagnosis rates in which a 10% increase and decrease was applied to the diagnosis rate. Breakdowns by total, undiagnosed and diagnosed CKD are provided. The error bars represent the ±10% difference in diagnosis rate.

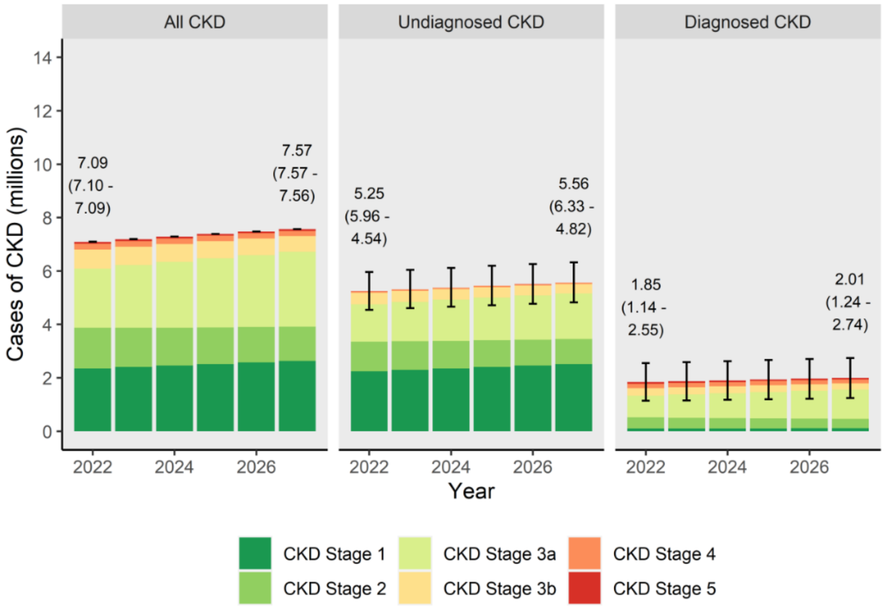

Germany: Prevalence of CKD following sensitivity analysis on diagnosis rates in which a 10% increase and decrease was applied to the diagnosis rate. Breakdowns by total, undiagnosed and diagnosed CKD are provided. The error bars represent the ±10% difference in diagnosis rate.

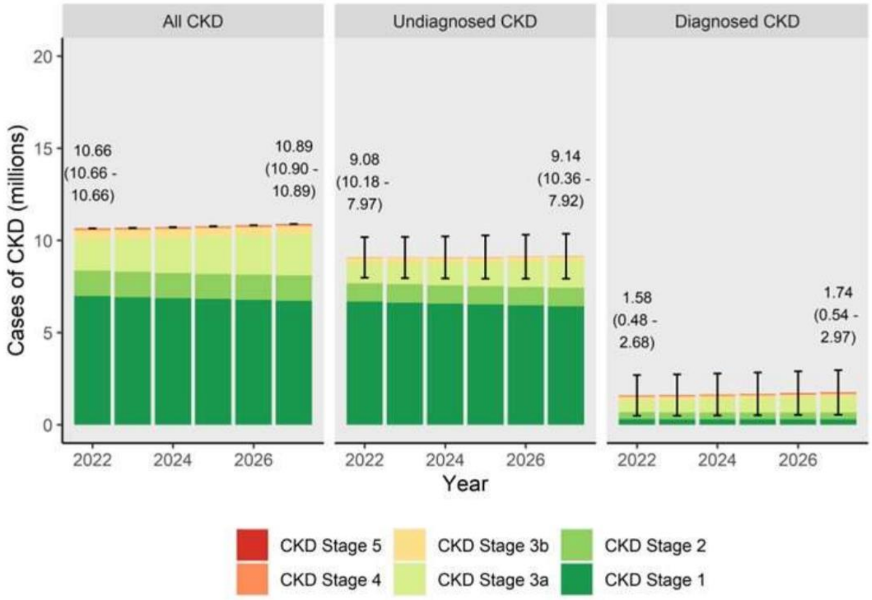

Greece: Prevalence of CKD following sensitivity analysis on diagnosis rates in which a 10% increase and decrease was applied to the diagnosis rate. Breakdowns by total, undiagnosed and diagnosed CKD are provided. The error bars represent the ±10% difference in diagnosis rate.

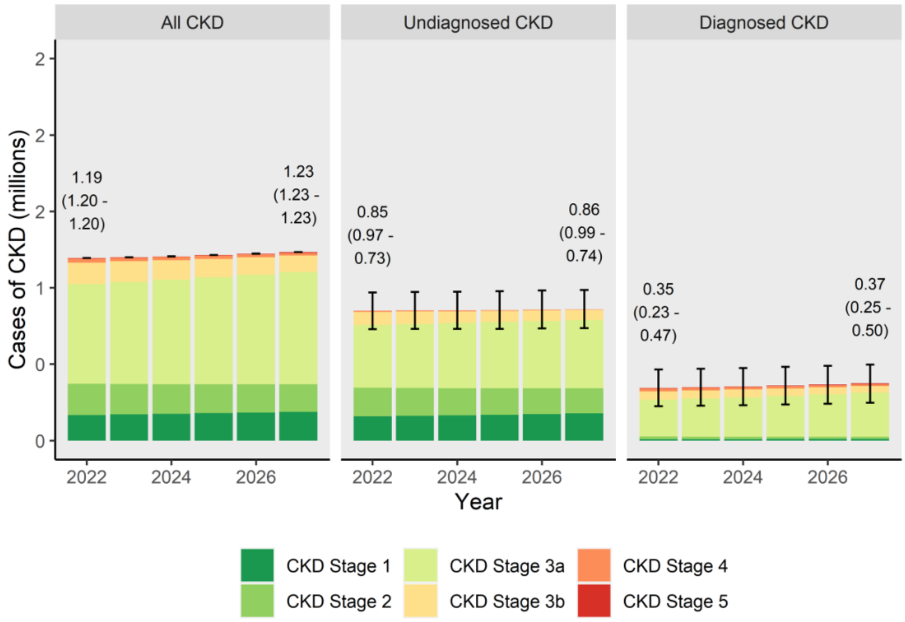

Hungary: Prevalence of CKD following sensitivity analysis on diagnosis rates in which a 10% increase and decrease was applied to the diagnosis rate. Breakdowns by total, undiagnosed and diagnosed CKD are provided. The error bars represent the ±10% difference in diagnosis rate.

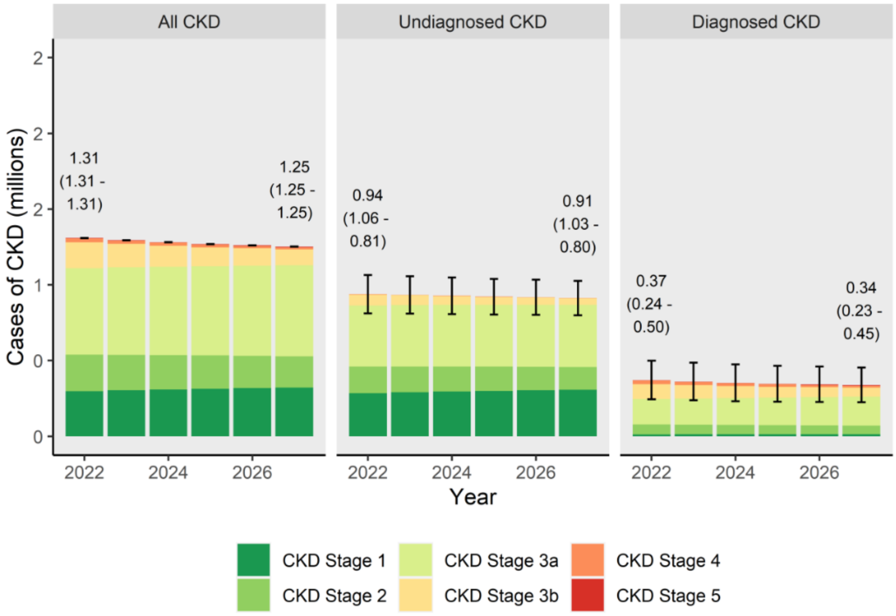

India: Prevalence of CKD following sensitivity analysis on diagnosis rates in which a 10% increase and decrease was applied to the diagnosis rate. Breakdowns by total, undiagnosed and diagnosed CKD are provided. The error bars represent the ±10% difference in diagnosis rate.

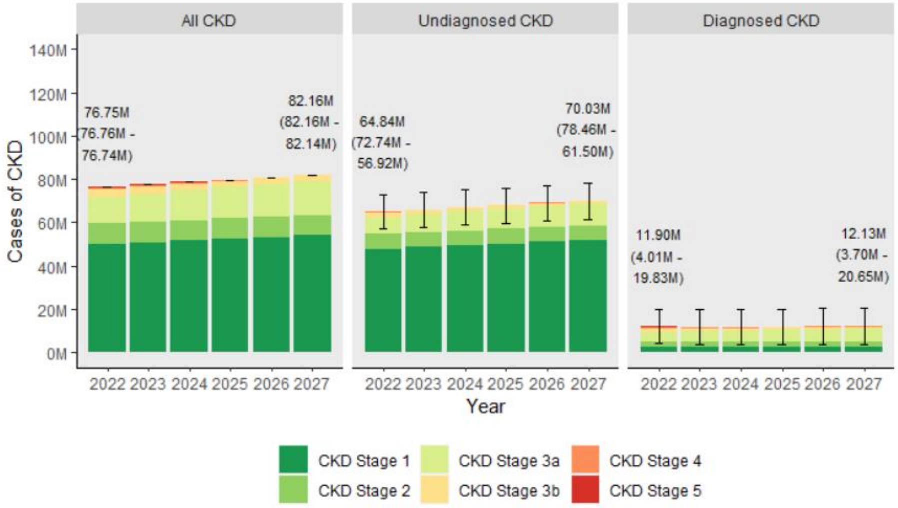

Israel: Prevalence of CKD following sensitivity analysis on diagnosis rates in which a 10% increase and decrease was applied to the diagnosis rate. Breakdowns by total, undiagnosed and diagnosed CKD are provided. The error bars represent the ±10% difference in diagnosis rate.

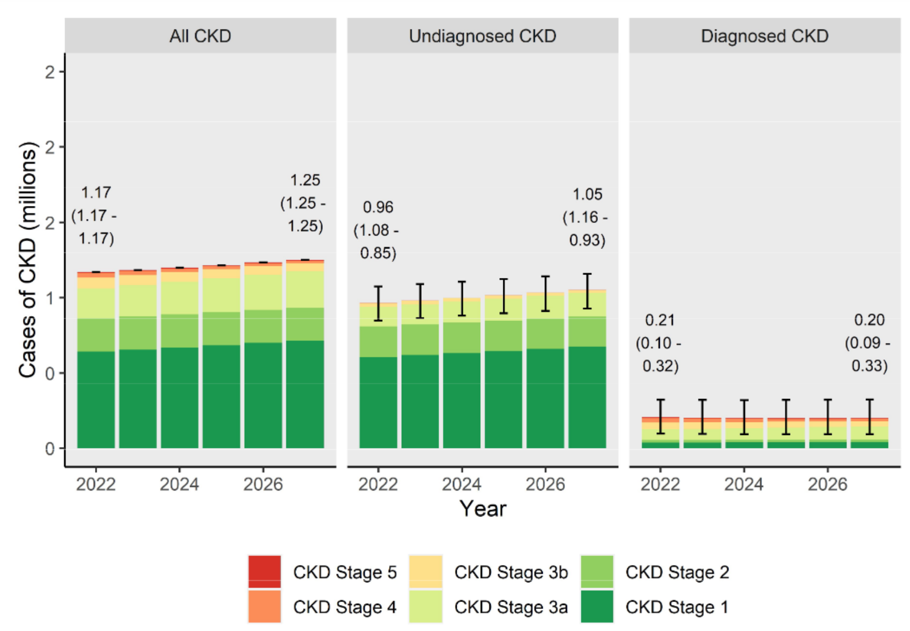

Italy: Prevalence of CKD following sensitivity analysis on diagnosis rates in which a 10% increase and decrease was applied to the diagnosis rate. Breakdowns by total, undiagnosed and diagnosed CKD are provided. The error bars represent the ±10% difference in diagnosis rate.

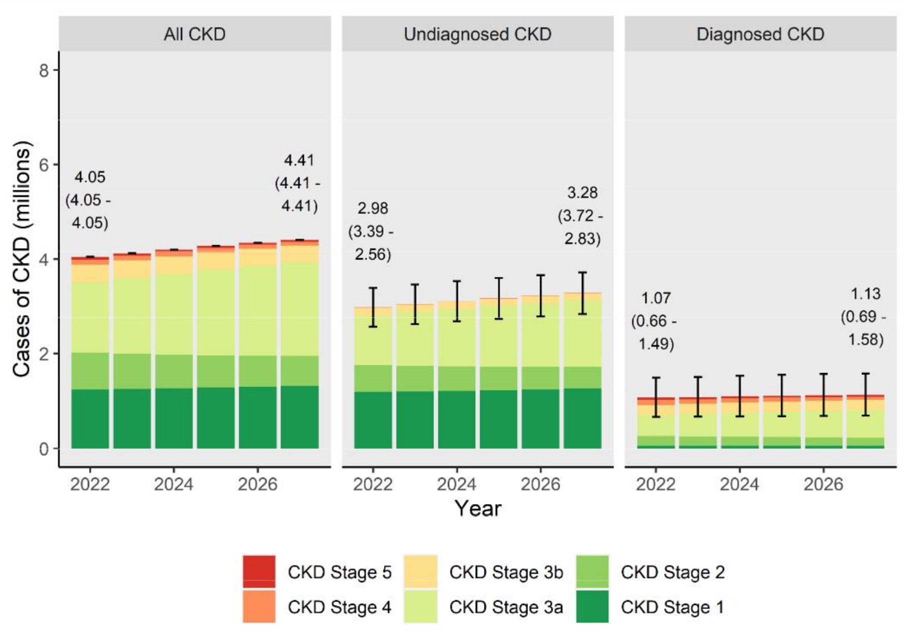

Japan: Prevalence of CKD following sensitivity analysis on diagnosis rates in which a 10% increase and decrease was applied to the diagnosis rate. Breakdowns by total, undiagnosed and diagnosed CKD are provided. The error bars represent the ±10% difference in diagnosis rate.

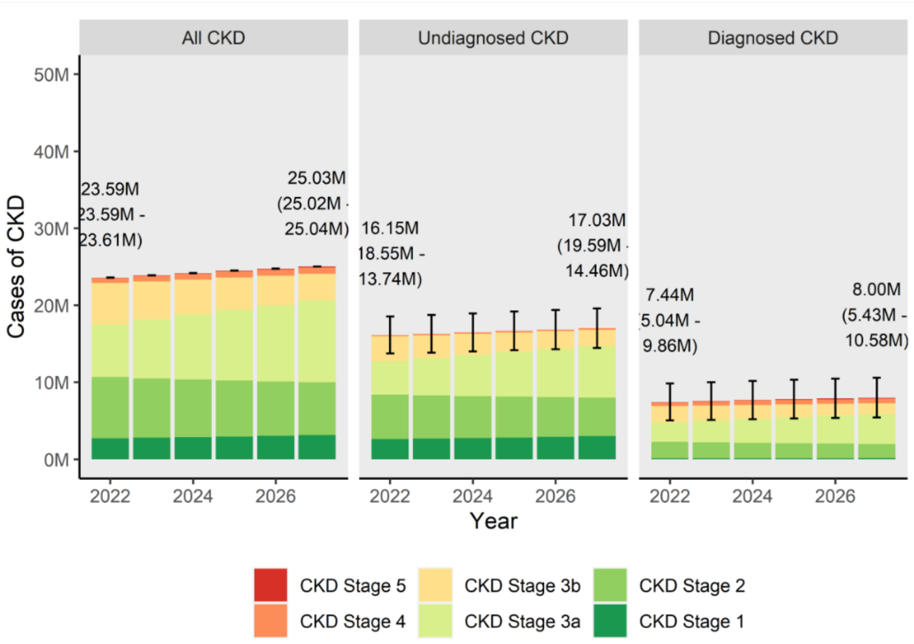

Mexico: Prevalence of CKD following sensitivity analysis on diagnosis rates in which a 10% increase and decrease was applied to the diagnosis rate. Breakdowns by total, undiagnosed and diagnosed CKD are provided. The error bars represent the ±10% difference in diagnosis rate.

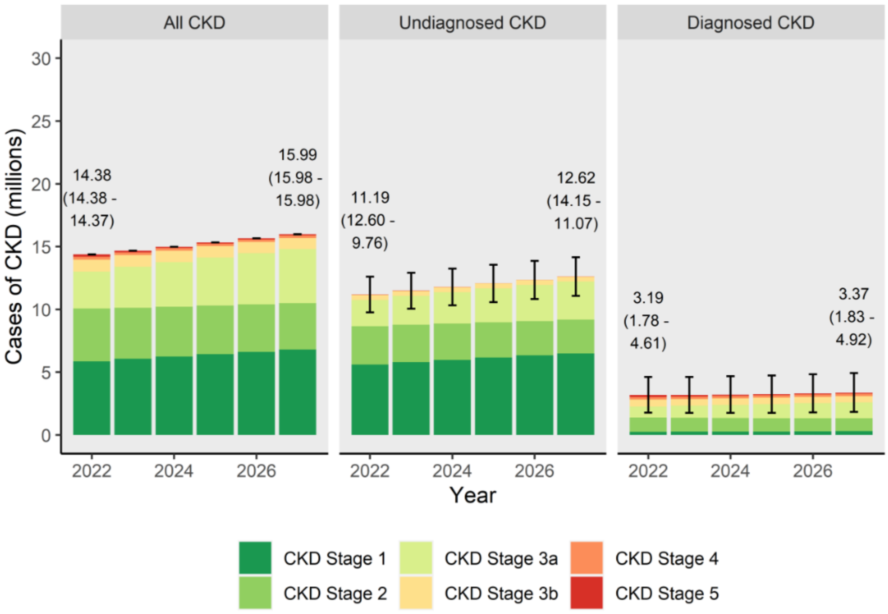

Netherlands: Prevalence of CKD following sensitivity analysis on diagnosis rates in which a 10% increase and decrease was applied to the diagnosis rate. Breakdowns by total, undiagnosed and diagnosed CKD are provided. The error bars represent the ±10% difference in diagnosis rate.

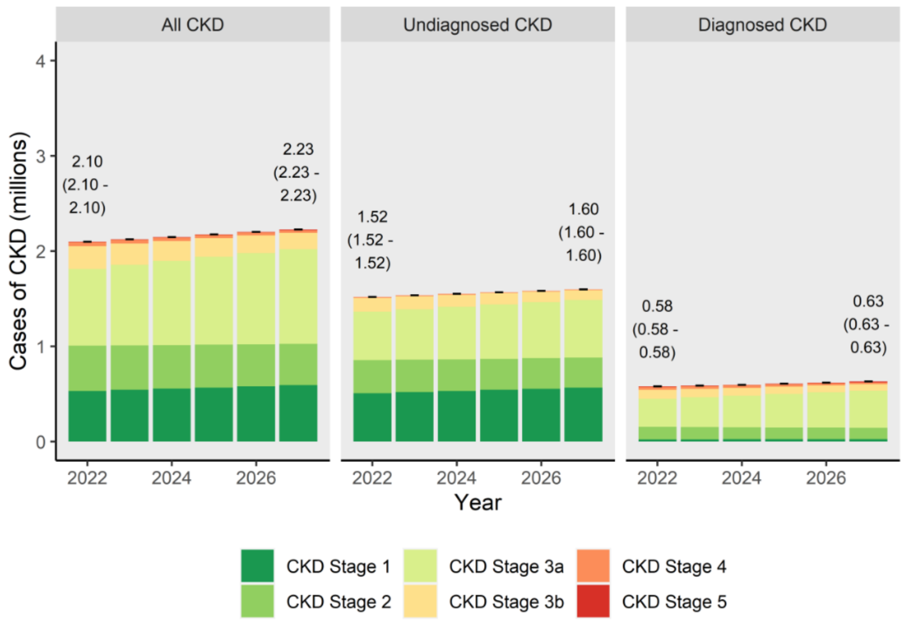

Philippines: Prevalence of CKD following sensitivity analysis on diagnosis rates in which a 10% increase and decrease was applied to the diagnosis rate. Breakdowns by total, undiagnosed and diagnosed CKD are provided. The error bars represent the ±10% difference in diagnosis rate.

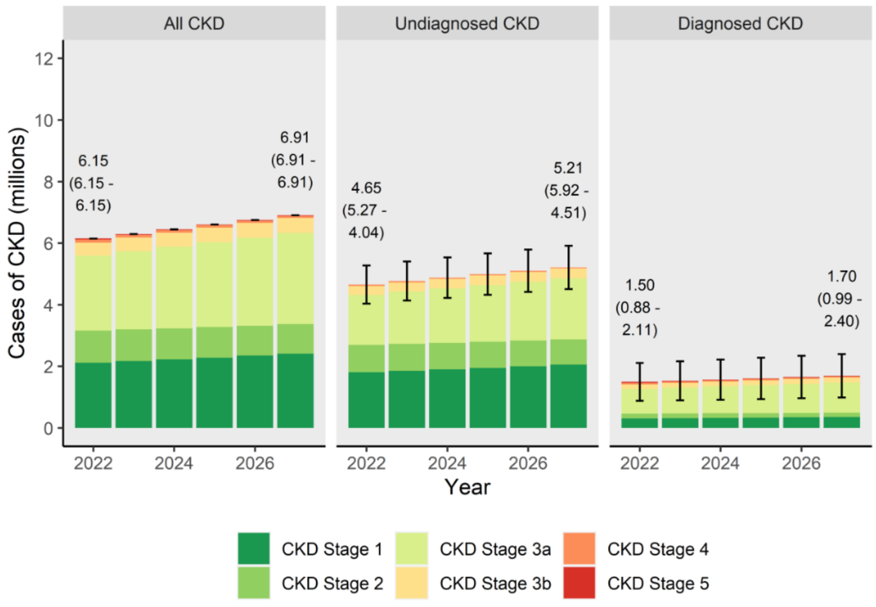

Poland: Prevalence of CKD following sensitivity analysis on diagnosis rates in which a 10% increase and decrease was applied to the diagnosis rate. Breakdowns by total, undiagnosed and diagnosed CKD are provided. The error bars represent the ±10% difference in diagnosis rate.

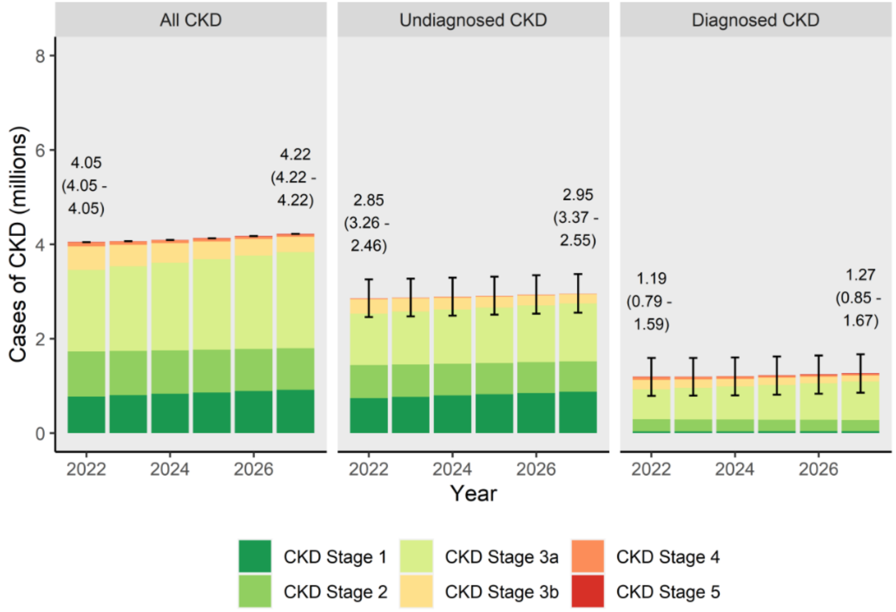

Romania: Prevalence of CKD following sensitivity analysis on diagnosis rates in which a 10% increase and decrease was applied to the diagnosis rate. Breakdowns by total, undiagnosed and diagnosed CKD are provided. The error bars represent the ±10% difference in diagnosis rate.

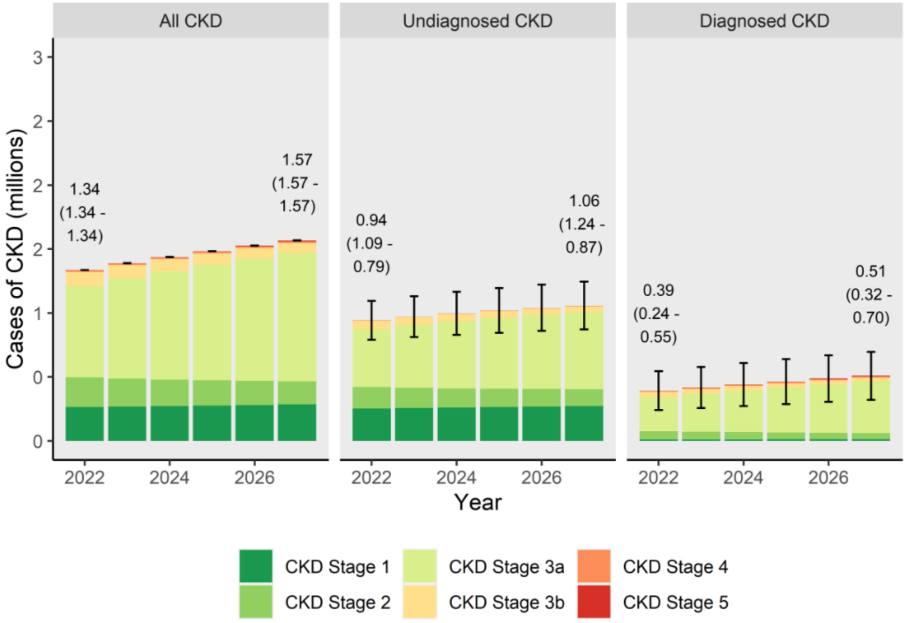

Saudi Arabia: Prevalence of CKD following sensitivity analysis on diagnosis rates in which a 10% increase and decrease was applied to the diagnosis rate. Breakdowns by total, undiagnosed and diagnosed CKD are provided. The error bars represent the ±10% difference in diagnosis rate.

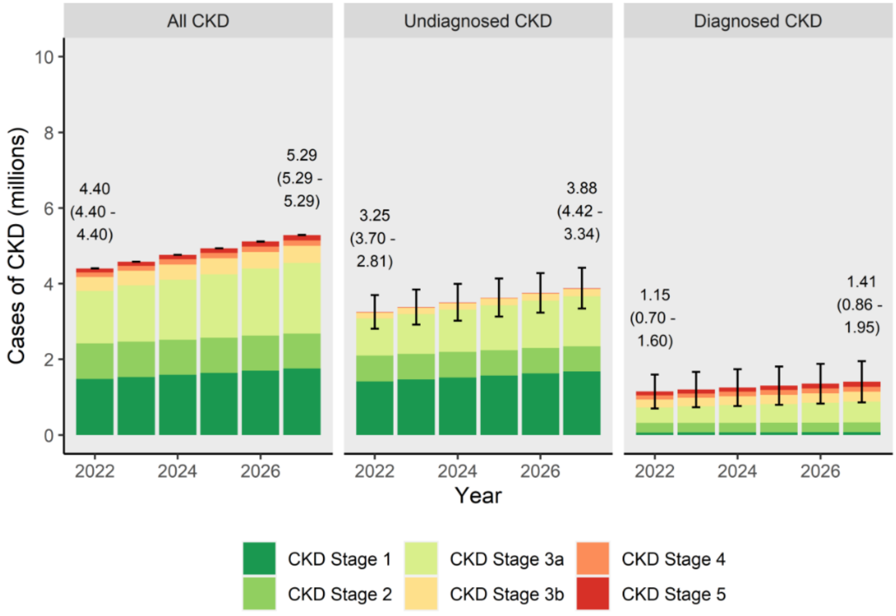

Singapore: Prevalence of CKD following sensitivity analysis on diagnosis rates in which a 10% increase and decrease was applied to the diagnosis rate. Breakdowns by total, undiagnosed and diagnosed CKD are provided. The error bars represent the ±10% difference in diagnosis rate.

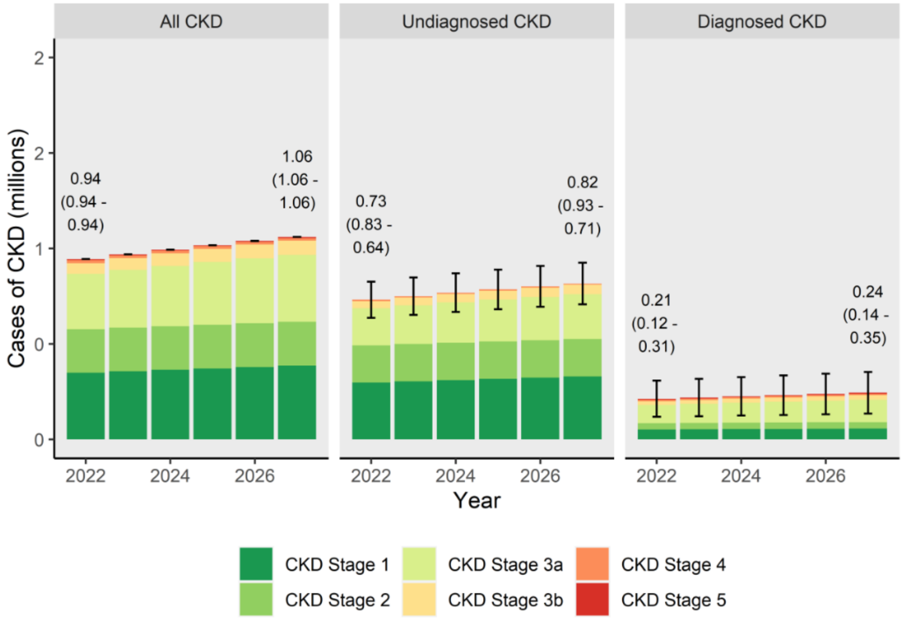

South Korea: Prevalence of CKD following sensitivity analysis on diagnosis rates in which a 10% increase and decrease was applied to the diagnosis rate. Breakdowns by total, undiagnosed and diagnosed CKD are provided. The error bars represent the ±10% difference in diagnosis rate.

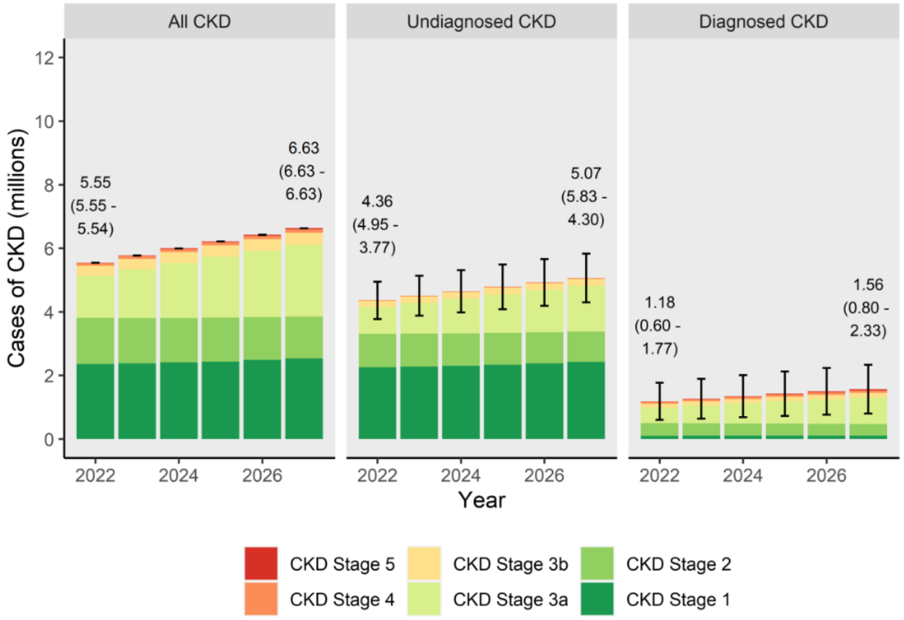

Spain: Prevalence of CKD following sensitivity analysis on diagnosis rates in which a 10% increase and decrease was applied to the diagnosis rate. Breakdowns by total, undiagnosed and diagnosed CKD are provided. The error bars represent the ±10% difference in diagnosis rate.

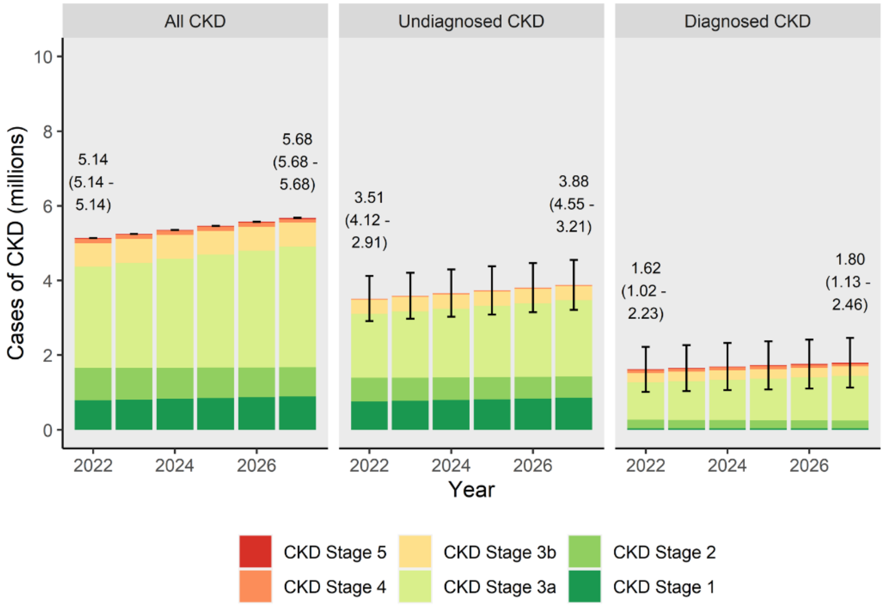

Sweden: Prevalence of CKD following sensitivity analysis on diagnosis rates in which a 10% increase and decrease was applied to the diagnosis rate. Breakdowns by total, undiagnosed and diagnosed CKD are provided. The error bars represent the ±10% difference in diagnosis rate.

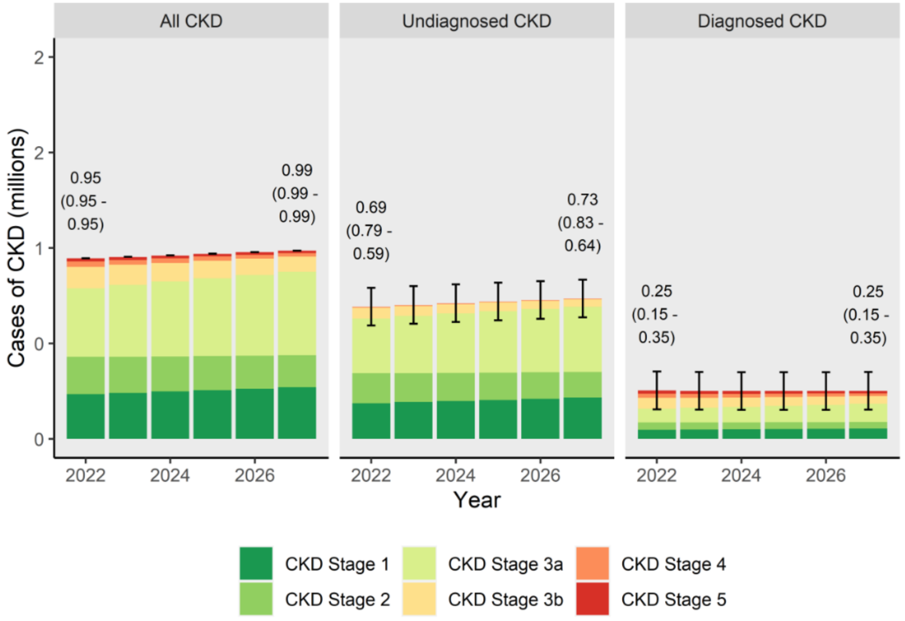

Taiwan: Prevalence of CKD following sensitivity analysis on diagnosis rates in which a 10% increase and decrease was applied to the diagnosis rate. Breakdowns by total, undiagnosed and diagnosed CKD are provided. The error bars represent the ±10% difference in diagnosis rate.

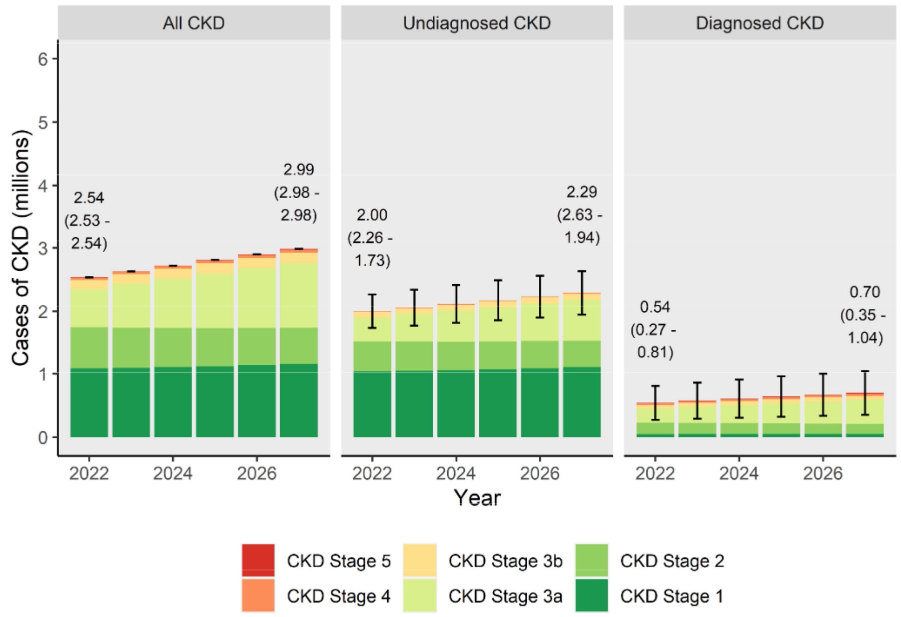

Thailand: Prevalence of CKD following sensitivity analysis on diagnosis rates in which a 10% increase and decrease was applied to the diagnosis rate. Breakdowns by total, undiagnosed and diagnosed CKD are provided. The error bars represent the ±10% difference in diagnosis rate.

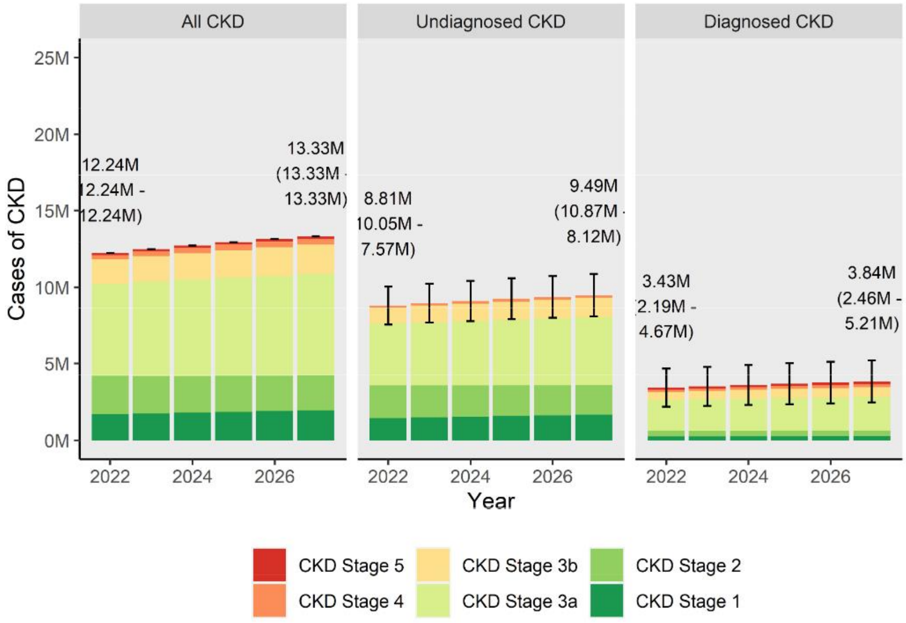

Türkiye: Prevalence of CKD following sensitivity analysis on diagnosis rates in which a 10% increase and decrease was applied to the diagnosis rate. Breakdowns by total, undiagnosed and diagnosed CKD are provided. The error bars represent the ±10% difference in diagnosis rate.

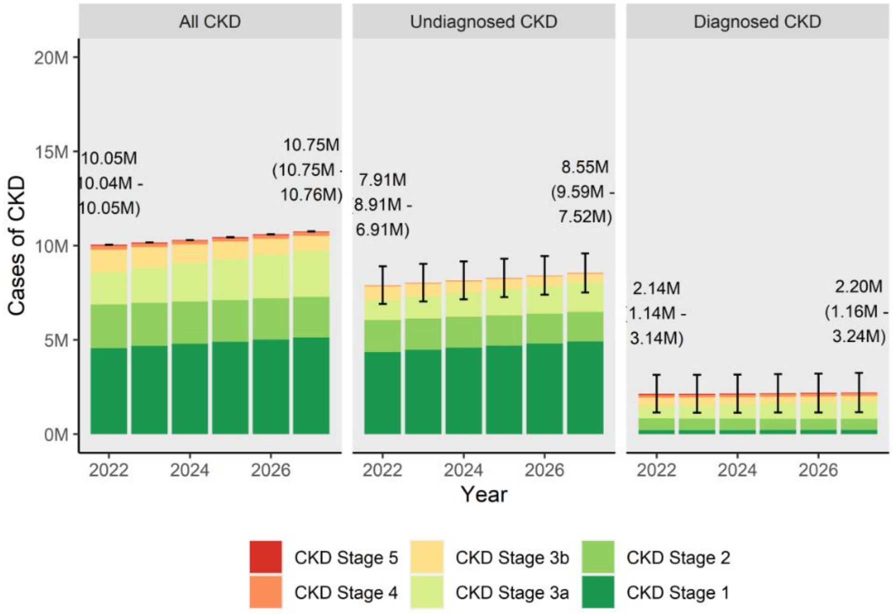

UAE: Prevalence of CKD following sensitivity analysis on diagnosis rates in which a 10% increase and decrease was applied to the diagnosis rate. Breakdowns by total, undiagnosed and diagnosed CKD are provided. The error bars represent the ±10% difference in diagnosis rate.

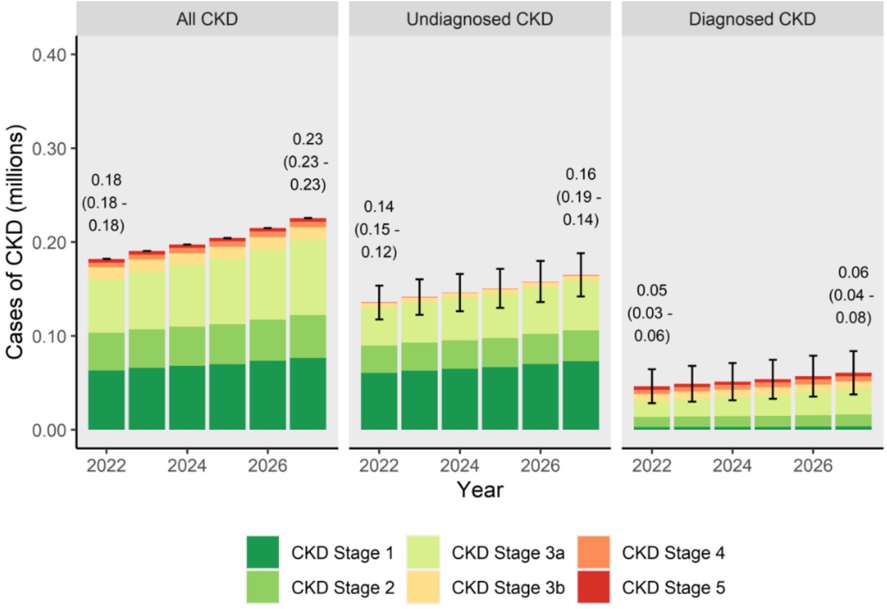

UK: Prevalence of CKD following sensitivity analysis on diagnosis rates in which a 10% increase and decrease was applied to the diagnosis rate. Breakdowns by total, undiagnosed and diagnosed CKD are provided. The error bars represent the ±10% difference in diagnosis rate.

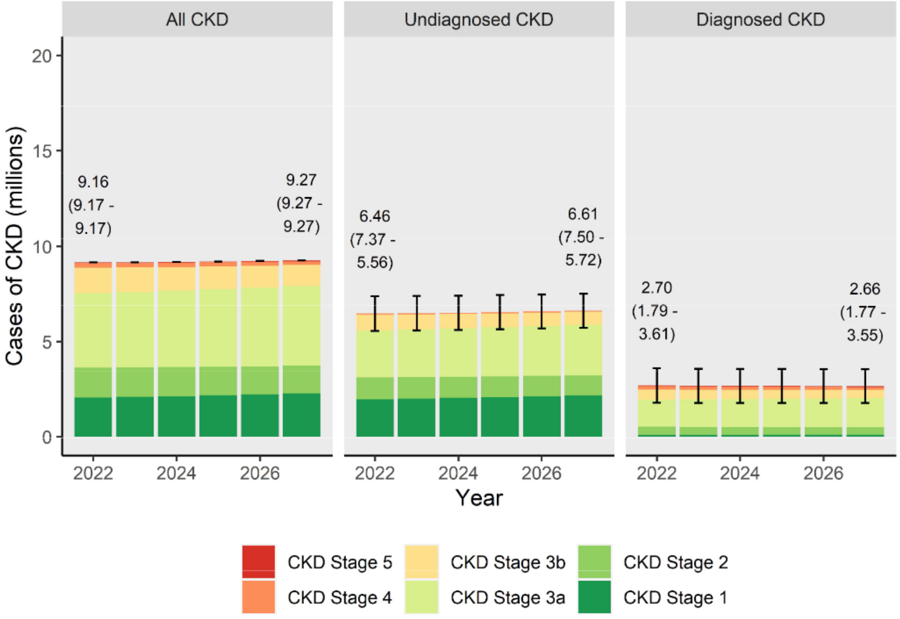

USA: Prevalence of CKD following sensitivity analysis on diagnosis rates in which a 10% increase and decrease was applied to the diagnosis rate. Breakdowns by total, undiagnosed and diagnosed CKD are provided. The error bars represent the ±10% difference in diagnosis rate.

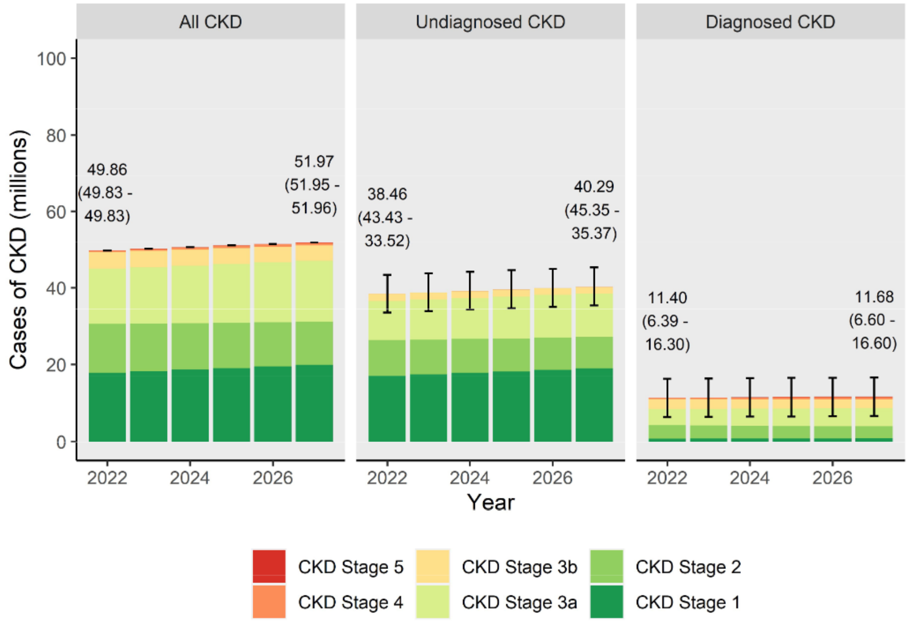

Supplementary references

Table S2 references

1. Australian Bureau of Statistics. Australian Health Survey: Biomedical Results for Chronic Diseases, 2011–12. 2013. https://www.abs.gov.au/statistics/health/health-conditions-and-risks/australian-health-survey-biomedical-results-chronic-diseases/latest-release (accessed May 01, 2022).
2. Sparke C, Moon L, Green F, et al. Estimating the Total Incidence of Kidney Failure in Australia Including Individuals Who Are Not Treated by Dialysis or Transplantation. *Am J Kidney Dis* 2013; **61**(3): 413–19.
3. Australia and New Zealand Dialysis and Transplant Registry (ANZDATA). ANZDATA 43rd Annual Report 2020 (Data to 2019). 2021. https://www.anzdata.org.au/report/anzdata-43rd-annual-report-2020-data-to-2019/ (accessed Jun 01, 2022).
4. NHS Digital. Health Survey for England, 2016. 2017. https://digital.nhs.uk/data-and-information/publications/statistical/health-survey-for-england/health-survey-for-england-2016 (accessed Jul 29, 2020).
5. British Heart Foundation (BHF). BHF Statistics: Heart and circulatory disease statistics 2020. 2020. https://www.bhf.org.uk/what-we-do/our-research/heart-statistics (accessed Jun 01, 2022).
6. Barendregt J, van Oortmarssen G, Vos T, Murray C. A generic model for the assessment of disease epidemiology: the computational basis of DisMod II. *Popul Health Metr* 2003; **1**: 4.
7. US Renal Data System, USRDS 2009 Annual Data Report: Atlas of Chronic Kidney Disease and End-Stage Renal Disease in the United States. 2009. https://www.usrds.org/2009/pdf/V1_06_09.pdf. (accessed Aug 01, 2022).
8. Australian Bureau of Statistics. Heart, stroke and vascular disease. 2018. https://www.abs.gov.au/statistics/health/health-conditions-and-risks/heart-stroke-and-vascular-disease/2017-18#data-download (accessed Jul 01 2022).
9. Deloitte Access Economics. Economic impact of stroke in Australia. 2020. https://www2.deloitte.com/content/dam/Deloitte/au/Documents/Economics/deloitte-au-dae-economic-impact-stroke-report-061120.pdf (accessed Jun 01, 2022).
10. Masson P, Webster A, Hong M, Turner R, Lindley R, Craig J. Chronic kidney disease and the risk of stroke: a systematic review and meta-analysis. *Nephrol Dial Transplant* 2015; **30**(7): 1162–9.
11. United Nations. Deaths by age groups, male. World population prospects 2019. https://population.un.org/wpp/Download/Archive/Standard/ (accessed May 4, 2022).
12. United Nations. Deaths by age groups, female. World population prospects 2019. https://population.un.org/wpp/Download/Archive/Standard/ (accessed May 4, 2022).
13. Levey AS, de Jong P, Coesh J, et al. The definition, classification, and prognosis of chronic kidney disease: a KDIGO Controversies Conference report. *Kidney Int* 2011; **80**(1): 17–28.
14. Hirst JA, Hill N, O'Callaghan C, et al. Prevalence of chronic kidney disease in the community using data from OxRen: A UK population-based cohort study. *Br J Gen Pract* 2020; **70**(693): e285–93.
15. Van Pottelbergh G, Bartholomeeusen S, Buntinx F, Degryse J. The prevalence of chronic kidney disease in a Flemish primary care morbidity register. *Age Ageing* 2012; **41**(2): 231–3.
16. O'Callaghan CA, Shine B, Lasserson DS. Chronic kidney disease: a large-scale population-based study of the effects of introducing the CKD-EPI formula for eGFR reporting. *BMJ Open* 2011; **1**(2): e000308.
17. Cleemput I, Beguin C, de la Kethulle Y, et al. Organisation and financing of chronic dialysis in Belgium KCE reports 124C. 2010. https://kce.fgov.be/sites/default/files/atoms/files/d20101027313.pdf (accessed May 01, 2022).
18. Belgian Society of Nephrology (NBVN). Jaarrapport NBVN 2019: Dialyse & Transplantatie. 2019. http://www.nbvn.be/data/Publieke-website-NBVN---jaarrapport-NBVN-2019---dialyse-&-transplantatie.pdf (accessed Apr 01, 2022). [Dutch.]
19. UK Renal Registry. 20th Annual Report. 2018. https://ukkidney.org/sites/renal.org/files/publication/file-attachments/Full%20Annual-Report_0.pdf (accessed May 01, 2022).

20. Barzilay JI, Davis B, Pressel S, et al. The effects of eGFR change on CVD, renal, and mortality outcomes in a hypertensive cohort treated with 3 different antihypertensive medications. *Am J Hypertens* 2018; **31**(5): 609–14.
21. Devroey D, Van Casteren V. The incidence and first-year mortality of heart failure in Belgium: A 2-year nationwide prospective registration. *Int J Clin Pract* 2010; **64**(3): 330–5.
22. Smeets M, Vaes B, Mamouris P, et al. Burden of heart failure in Flemish general practices: A registry-based study in the Intego database. *BMJ Open* 2019; **9**(1): e022972.
23. Belgian Federal Public Service. Ischemic heart disease. 2016. 2021. https://www.healthybelgium.be/en/health-status/non-communicable-diseases/ischemic-heart-disease#:~:text=In%202016%2C%20the%20number%20of,men%20in%20all%20age%20groups (accessed Aug 01, 2022).
24. Moran A, Shen A, Turner-Lloveras D, et al. Utility of self-reported diagnosis and electrocardiogram Q-waves for estimating myocardial infarction prevalence: an international comparison study. *Heart* 2012; **98**(22): 1660–6.
25. Institute for Health Metrics and Evaluation (IHME). Global Burden of Disease Study 2019 (GBD 2019) Data Resources: Ischemic heart disease, stroke. 2019. http://ghdx.healthdata.org/gbd-2019 (accessed Aug 01, 2021).
26. Piccolli AP, do Nascimento MM, Riella MC. Prevalence of chronic kidney disease in a population in southern Brazil (Pro-Renal Study). *J Bras Nefrol* 2017; **39**(4): 384–90.
27. Pyart R, Evans K, Steenkamp R, et al. The 21st UK Renal Registry Annual Report: A Summary of Analyses of Adult Data in 2017. *Nephron* 2020; **144**(2): 59–66.
28. Organización Nacional de Trasplantes. Registro Español de Enfermos Renales (REER). 2018. http://www.registrorenal.es/ (accessed Jun 01, 2022). [Spanish.]
29. Machado E, Caiaffa W, César C, et al. Iniquities in the access to renal transplant for patients with end-stage chronic renal disease in Brazil. *Cad Saude Publica* 2011; **27**(supp 2): S284–97.
30. Brazilian Society of Nephrology, Brazilian dialysis registry. 1997. http://gamba.epm.br/registro/97/rghd97.html (accessed May 01, 2022).
31. Neves PDMM, Sesso R, Thomé F, Lugon J, Nascimento M. Brazilian dialysis survey 2019. *J Bras Nefrol* 2021; **43**(2): 217–27.
32. Oliveira JGG, Monteiro MS, Teixeira JF, et al. Humoral immune response after kidney transplantation is enhanced by acute rejection and urological obstruction and is downregulated by mycophenolate mofetil treatment. *Transpl Int* 2005; **18**(11): 1286–91.
33. Ruiz-Hurtado G, Ruilope LM, de la Sierra A, et al. Association between high and very high albuminuria and nighttime blood pressure: influence of diabetes and chronic kidney disease. *Diabetes Care* 2016; **39**(10): 1729–37.
34. Fernández-Gassó L, Hernando-Arizaleta L, Palomar-Rodríguez J, Abellán-Pérez M, Hernández-Vicente Á, Pascual-Figal D. Population-based Study of First Hospitalizations for Heart Failure and the Interaction Between Readmissions and Survival. *Rev Esp Cardiol (Engl Ed)* 2019; **72**(9): 740–8.
35. Oliveira G, Brant L, Polanczyk C, et al. Cardiovascular statistics – Brazil 2020. *Arq Bras Cardiol* 2020; **115**(3): 308–439.
36. Forcadell MJ, Vila-Córcoles A, de Diego C, Ochoa-Gondar O, Satué E. Incidence and mortality of myocardial infarction among Catalonian older adults with and without underlying risk conditions: The CAPAMIS study. *Eur J Prev Cardiol* 2018; **25**(17): 1822–30.
37. Vega T, Zurriaga O, Ramos J, et al. Stroke in Spain: Epidemiologic Incidence and Patterns; A Health Sentinel Network Study. *J Stroke Cerebrovasc Dis* 2009; **18**(1): 11–16.
38. Bakris G, et al. Prevalence and factors associated with undiagnosed chronic kidney disease in diabetes mellitus. 2020. https://casehippo.com/spa/symposium/national-kidney-foundation-2019-spring-clinical-meetings/event/gallery/abstracts?abstractId=119 (accessed Aug 01, 2021).
39. Canadian Institute for Health Information. Canadian Organ Replacement Register (CORR). 2021. https://www.cihi.ca/en/canadian-organ-replacement-register-corr (accessed Jul 29, 2020).
40. United States Renal Data System (USRDS). USRDS Annual Data Report: Epidemiology of kidney disease in the United States 2021. 2021. https://adr.usrds.org/2021 (accessed Aug 01, 2021).
41. Armstrong M, Weaver R, Pannu N, Services AH. Prevalence and Quality of Care in Chronic Kidney Disease Alberta Kidney Care Report. 2019. https://www.albertahealthservices.ca/assets/about/scn/ahs-scn-kh-ckd-report-2019.pdf (accessed Aug 01, 2022).

42. Centers for Disease Control and Prevention. National Health and Nutrition Examination Survey. 2020. https://www.cdc.gov/nchs/nhanes/index.htm. (accessed Aug 01, 2022).
43. Government of Canada. Canadian Chronic Disease Surveillance System (CCDSS). 2018. https://health-infobase.canada.ca/ccdss/data-tool/ (accessed Jul 30, 2020).
44. Zhang L, Wang F, Wang L, et al. Prevalence of chronic kidney disease in China: A cross-sectional survey. *Lancet* 2012; **379**: 815–22.
45. Japanese Society of Nephrology. CKD Medical Care Guide. 2012. https://jsn.or.jp/guideline/pdf/CKDguide2012.pdf (accessed May 01, 2022).
46. Watanabe Y, Yamagata K, Nishi S, et al. Japanese Society for Dialysis Therapy Clinical Guideline for "Hemodialysis Initiation for Maintenance Hemodialysis". *Ther Apher Dial* 2015; **19**: 93–107.
47. Zhang L, Zhao M, Zuo L, et al. China Kidney Disease Network (CK-NET) 2015 Annual Data Report. *Kidney Int Suppl* 2019; **9**(1): e1–81.
48. Chinese National Renal Data System. 国家肾脏病医疗质量控制中心. http://www.cnrds.net/TxLogin (accessed May 01, 2022).
49. Sun L, Zou L, Han YC, et al. Forecast of the incidence, prevalence and burden of end-stage renal disease in Nanjing, China to the Year 2025. *BMC Nephrol* 2016; **17**: 60.
50. Xue W, Tian P, Xiang H, et al. Outcomes for primary kidney transplantation from donation after Citizens' death in China: a single-center experience of 367 cases. *BMC Health Serv Res* 2017; **17**(1): 250.
51. Sun XQ, Li JS, Sui YY, et al. Investigation of epidemiologic features and treatment of chronic heart failure in some rural parts of Liaoning province. *Chinese J Pract Intern Med* 2009; **11**: 1000–2.
52. Yamato M, Aizu H, Kurohara K, Tanaka J. Japanese local language paper: https://www.kyoukaikenpo.or.jp/~/media/Files/honbu/cat740/houkokusho/h28/houkokusho_11.pdf (accessed Aug 31, 2023. [Japanese.]
53. Otero A, de Francisco A, Gayoso P, et al. Prevalence of chronic renal disease in Spain: results of the EPIRCE study. *Nefrología* 2010; **30**(1): 78–86.
54. Gorostidi M, Sánchez-Martínez M, Ruilope L, et al. Chronic kidney disease in Spain: Prevalence and impact of accumulation of cardiovascular risk factors *Nefrología*, 2018; **38**(6): 606–15.
55. Cuenta de Alto Costo. Situación de la enfermedad renal crónica, la hipertensión arterial y la diabetes mellitus en Colombia 2020. 2021. https://cuentadealtocosto.org/site/erc/situacion-de-la-enfermedad-renal-cronica-la-hipertension-arterial-y-la-diabetes-mellitus-en-colombia-2020/ (accessed May 11, 2022). [Spanish.]
56. Urrego A. Riñón, higado y corazón, los órganos más trasplantados en el último año en Colombia. 2022. https://www.larepublica.co/salud-ejecutiva/rinon-higado-y-corazon-los-organos-mas-trasplantados-en-el-ultimo-ano-en-colombia-3312287 (accessed May 11, 2022). [Spanish.]
57. Estupiñán-Bohórquez A, Acosta-Reyes J, Viasus-Pérez D, García-López A, Patino-Jaramillo N, Girón-Luque F. Trasplante renal de donantes con criterios expandidos en la región Caribe colombiana. *Nefro Latinoam* 2021; **18**: 119–27. [Spanish].
58. Gómez E. Introduction, epidemiology of heart failure and the history of cardiac failure clinics in Colombia. *Rev Colomb Cardiol* 2016; **23**(S1): 6–12. [Spanish.]
59. Sistema Integrado de Información de la Protección Social (SISPRO) and Congreso de la República Colombiana. Ponencia segundo debate PL 017 DE 2020 enfermedades ACV. 2021. https://www.camara.gov.co/camara/visor?doc=/sites/default/files/2021-05/Ponencia%20Segundo%20Debate%20PL%20017%20DE%202020%20Enfermedades%20ACV.pdf (accessed May 11, 2022). [Spanish.]
60. Gómez-Mesa J, et al. Colombian registry of heart failure (RECOLFACA): results. *Rev Colomb Cardiol* 2021; **28**(4): 334–44. [Spanish.]
61. Bedoya-Rios C, Mendoz-Lozano J, Nieto-Cardenas O. Prevalence of myocardial infarction in a cardiovascular risk program of a health care institution in Armenia-Quindío. *Rev Colomb Cardiol* 2016; **23**(6): 561–7. [Spanish.]
62. El Hospital. Bogotá is the Colombian city with the highest prevalence of stroke. Internal medicine and subspecialities. 2018. https://www.elhospital.com/temas/Bogota-es-la-ciudad-colombiana-con-mayor-prevalencia-de-ataque-cerebrovascular+127097#:~:text=Seg%C3%BAn%20la%20Asociaci%C3%B3n%20Colombiana%20de,emergencia%20para%20su%20pronta%20atenci%C3%B3n (accessed May 13, 2022). [Spanish.]

63. Danish Society of Nephrology (DSN). Kronisk nyresygdom: Analysemetoder og klinisk evaluering. 2015. https://nephrology.dk/vejledninger/ckd-mbd/kronisk-nyresygdom/ (accessed Jul 01, 2022). [Danish.]
64. Danish Nephrological Society National Register (DNSL). Nationwide database for patients with chronic renal failure: Report for 2020. 2020. https://www.sundhed.dk/content/cms/92/4692_dnsl-aarsrapport-2020---endelig.pdf (accessed Jul 01, 2022). [Danish.]
65. Sundhed DK. Kidney Transplant. 2019. https://www.sundhed.dk/borger/patienthaandbogen/nyrer-og-urinveje/sygdomme/diverse/nyretransplantation/ (accessed Jul 01, 2022). [Danish.]
66. Hjerte Foreningen. Heart failure: new cases (35+ years). 2018. https://hjerteforeningen.shinyapps.io/HjerteTal/?_inputs_&agCVD=%22age%22&bar=%22cvd%22&year=%222018%22&varCVD=%22v1%22&oCVD=%22d8%22 (accessed Jan 01, 2022).
67. Hjerte Foreningen. Heart failure: total cases (35+ year olds). 2018. https://hjerteforeningen.shinyapps.io/HjerteTal/?_inputs_&agCVD=%22age%22&bar=%22cvd%22&year=%222018%22&varCVD=%22v2%22&oCVD=%22d8%22 (accessed Jan 01, 2022).
68. Hjerte Foreningen. Myocardial embolism (AMI): new cases (35+ years). 2018. https://hjerteforeningen.shinyapps.io/HjerteTal/?_inputs_&agCVD=%22age%22&bar=%22cvd%22&year=%222018%22&varCVD=%22v1%22&oCVD=%22d3%22 (accessed Jan 01, 2022).
69. Hjerte Foreningen. Vascular diseases of the brain (incl. stroke): new cases (35+ years). 2018. https://hjerteforeningen.shinyapps.io/HjerteTal/?_inputs_&agCVD=%22age%22&bar=%22cvd%22&year=%222018%22&varCVD=%22v1%22&oCVD=%22d10%22 (accessed Jan 01, 2022).
70. Hjerte Foreningen. Vascular diseases of the brain (incl. stroke): total cases (35+ year olds). 2018. https://hjerteforeningen.shinyapps.io/HjerteTal/?_inputs_&agCVD=%22age%22&bar=%22cvd%22&year=%222018%22&varCVD=%22v2%22&oCVD=%22d10%22 (accessed Jan 01, 2022).
71. Agence de le biomédecine. REIN Annual Report 2018. https://www.agence-biomedecine.fr/IMG/pdf/rapport_rein_2018_v2.pdf (accessed Nov 01, 2022). [French.]
72. Girndt M, Trocchi P, Scheidt-Nave C, Stang A. The Prevalence of Renal Failure. Results from the German Health Interview and Examination Survey for Adults, 2008-2011 (DEGS1). *Dtsch Ärztebl Int* 2016; **113**: 85–91.
73. Horl WH, Wanner CH. Dialyseverfahren in Klinik und Praxis: Technik und Klinik. Stuttgart, DE: Georg Thieme Verlag, 2004. [German.]
74. Frei U, Schober-Halstenberg HJ. Nieren-Ersatztherapie in Deutschland. 2008. https://www.bundesverband-niere.de/wp-content/uploads/2019/02/QuaSi-Niere-Bericht_2006-2007.pdf (accessed May 01, 2022). [German.]
75. Satayathum S, Pisoni L, McCullough K, et al. Kidney transplantation and wait-listing rates from the international Dialysis Outcomes and Practice Patterns Study (DOPPS). *Kidney Int* 2005; **68**: 330–7.
76. GBE Federation. Federal health reporting. 2021. https://www.gbe-bund.de/gbe/ (accessed Aug 01, 2022).
77. ERA-EDTA Registry. ERA-EDTA Registry Annual Report 2019. 2019. https://www.era-online.org/registry/AnnRep2019.pdf (accessed Jun 28, 2022).
78. Grandaliano. 61° Congresso Nazionale Società Italiana Nefrologia (SIN). Available from: https://congresso2020.eventisin.it/. (accessed Apr 01, 2022).
79. Piccinni C, Antonazzo I, Simonetti M, et al. The Burden of Chronic Heart Failure in Primary Care in Italy. *High Blood Press Cardiovasc Prev* 2017; **24**(2): 171–8.
80. Bosch L, Assmann P, de Grauw W, Schalk B, Biermans M. Heart failure in primary care: prevalence related to age and comorbidity. *Prim Health Care Res Dev* 2019; **20**: e79.
81. Regione Lombardia, Registro dell'infarto miocardico acuto nell'Asl di Brescia. Rapporto 2003–2009. https://www.ats-brescia.it/documents/3432658/70845777/RegistroIMA_2003_09.pdf/1dec3046-29b2-b0c8-850d-30f2d8615a61 (accessed Apr 01, 2022). [Italian.]
82. Hellenic Statistical Authority. Press Release: Health Research 2019. 2020. https://www.statistics.gr/el/statistics?p_p_id=documents_WAR_publicationsportlet_INSTANCE_qDQ8fBKKo4lN&p_p_lifecycle=2&p_p_state=normal&p_p_mode=view&p_p_cacheability=cacheLevelPage&p_p_col_id=column-2&p_p_col_count=4&p_p_col_pos=1&_documents_WAR_publicationsportlet_INSTA

NCE_qDQ8fBKKo4lN_javax.faces.resource=document&_documents_WAR_publicationsportlet_INSTANCE_qDQ8fBKKo4lN_ln=downloadResources&_documents_WAR_publicationsportlet_INSTANCE_qDQ8fBKKo4lN_documentID=461317&_documents_WAR_publicationsportlet_INSTANCE_qDQ8fBKKo4lN_locale=el (accessed Jun 01, 2022). [Greek].
83. Karantali E, Vemos K, Tsampalas E, et al. Temporal trends in stroke incidence and case-fatality rates in Arcadia, Greece: A sequential, prospective, population-based study. *Int J Stroke* 2022; **17**(1): 37–47.
84. Zdrojewski Ł, Zdrojewski T, Rutkowski M, et al. Prevalence of chronic kidney disease in a representative sample of the Polish population: results of the NATPOL 2011 survey. *Nephrol Dial Transplant* 2016; **31**(3): 433–9.
85. Chudek J, Wieczorowska-Tobis K, Zejda J, et al. The prevalence of chronic kidney disease and its relation to socioeconomic conditions in an elderly Polish population: results from the national population-based study PolSenior. *Nephrol Dial Transplant* 2014; **29**(5): 1073–82.
86. Hungarian Society of Nephrology. MANET 2016–2019. 2020. http://www.nephrologia.hu/info.aspx?sp=200 (accessed Aug 01, 2021). [Hungarian].
87. Anand S, Shivashankar R, Ali M, et al. Prevalence of chronic kidney disease in two major Indian cities and projections for associated cardiovascular disease. *Kidney Int* 2015; **88**: 178–85.
88. UK Renal Registry. UK Renal Registry 23rd Annual Report. 2021. https://ukkidney.org/sites/renal.org/files/publication/file-attachments/23rd_UKRR_ANNUAL_REPORT_0.pdf (accessed May 01, 2022).
89. Parameswaran S, Geda S, Rathi M, et al. Referral pattern of patients with end-stage renal disease at a public sector hospital and its impact on outcome. *Natl Med J India* 2011; **24**: 208–13.
90. Nitta K, Goto S, Masakane I, et al. Annual dialysis data report for 2018, JSDT Renal Data Registry: Survey methods, facility data, incidence, prevalence, and mortality. *Renal Replace Ther* 2020; **6**: 1–18.
91. The Japanese Society for Transplantation. Fact book on organ transplantation in Japan. 2020. http://www.asas.or.jp/jst/pdf/factbook/factbook2020.pdf (accessed Jun 01, 2022). [Japanese.]
92. Bharati J, Jha V. Global Dialysis Perspective: India. *Kidney360* 2020; **1**: 1143–7.
93. Centers for Disease Control & Prevention (CDC). National Health and Nutrition Examination Survey (NHANES). 2020. https://www.cdc.gov/nchs/nhanes/index.htm (accessed Jul 01, 2020).
94. E-Stat. Portal Site of Official Statistics of Japan. 2017. https://www.e-stat.go.jp/en (accessed Apr 01, 2022).
95. The Japanese Registry of All Cardiac and Vascular Diseases. Cardiovascular disease medical treatment fact-finding report, 2019. 2019. https://www.j-circ.or.jp/jittai_chosa/jittai_chosa2018web.pdf (accessed Apr 01, 2022).
96. Ministry of Health, Labour and Welfare, Japan. Draft budget outline for fiscal year 2014. 2019. https://www.mhlw.go.jp/english/policy/other/budget/dl/1st-fy2014-e.pdf (accessed Apr 01, 2022).
97. Das SK, Biswas A, Roy T, et al. A random sample survey for prevalence of major neurological disorders in Kolkata. *Indian J Med Res* 2006; **124**: 163–72.
98. Hebrew local language paper, 2021 https://www.gov.il/BlobFolder/reports/dialysis-in-israel-2020/he/files_publications_units_ICDC_dialysisinisrael2020.pdf (accessed Apr 01, 2022).
99. De Nicola L, Donfrancesco C, Minutolo R, et al. Prevalence and cardiovascular risk profile of chronic kidney disease in Italy: results of the 2008–12 National Health Examination Survey. *Nephrol Dial Transplant* 2015; **30**: 806–14
100. Valeria, M, Pasquale B, Franco B, et al. Ministero della Salute Documento di Indirizzo per la Malattia Renale Cronica. http://www.salute.gov.it/imgs/C_17_pubblicazioni_2244_allegato.pdf (accessed Jul 01, 2022). [Italian.]
101. Nordio M, Limido A, Postorino M. Present and future of kidney replacement therapy in Italy: the perspective from Italian Dialysis and Transplantation Registry (IDTR). *J Nephrol* 2020; **33**(6): 1195–1200.
102. Corso G, Bottacchi E, Giardini G, et al. Epidemiology of stroke in northern Italy: the Cerebrovascular Aosta Registry, 2004–2008. *Neurol Sci* 2013; **34**: 1071–81.
103. Yagisawa T, Mieno M, Ichimaru N, et al. Trends of kidney transplantation in Japan in 2018: data from the kidney transplant registry. *Ren Replace Ther* 2019; **5**: 1–14.
104. Takashima N, Arima H, Kita Y, et al. Incidence, management and short-term outcome of stroke in a general population of 1.4 million Japanese – Shiga stroke registry. *Circ J* 2017; **81**: 1636–46.

105. Cueto-Manzano AM, Cortés-Sanabria L, Martínez-Ramírez HR, Rojas-Campos E, Gómez-Navarro B, Castillero-Manzano M. Prevalence of Chronic Kidney Disease in an Adult Population. *Arch Med Res* 2014; **45**: 507–13.
106. United States Renal Data System (USRDS). USRDS Annual Data Report: Epidemiology of kidney disease in the United States. 2020. https://adr.usrds.org/2020/ (accessed Jun 01, 2022).
107. Garcia-Garcia G, Briseño-Rentería G, Luquín-Arellan VH, Gao Z, Gill J, Tonelli M. Survival among patients with kidney failure in Jalisco, Mexico. *J Am Soc Nephrol* 2007; **18**: 1922–7.
108. Farré N, Vela E, Clèries M, et al. Real world heart failure epidemiology and outcome: A population-based analysis of 88,195 patients. *PLoS One* 2017; **12**: e0172745.
109. Federatie Medisch Specialisten. Diabetic Nephropathy: Timing renal function replacement therapy in diabetic nephropathy. 2020. https://richtlijnendatabase.nl/richtlijn/diabetische_nefropathie/timing_nierfunctievervangende_therapie_bij_diabetische_nefropathie.html (accessed Jul 01, 2022). [Dutch.]
110. Nefrovisie. 2022. https://ivisualz.nl/ivisualz/chartFlash/charts\ (accessed Jul 01, 2022). [Dutch.]
111. Dutch Heart Foundation, et al. Cardiovascular disease in the Netherlands, 2021. [Chapter 3]. Den Haag, 2021 (December 2021). Available from: https://www.hartstichting.nl/getmedia/06fb9c92-a1f7-4135-a635-ff73680bfaa6/cijferboek-hartstichting-hart-vaatziekten-nederland-2016.pdf. [Dutch.]
112. Nang EEK, Khoo CM, Tai ES, et al. Is there a clear threshold for fasting plasma glucose that differentiates between those with and without neuropathy and chronic kidney disease? The Singapore Prospective Study Program. *Am J Epidemiol* 2009; **169**: 1454–62.
113. United States Renal Data System (USRDS). Annual data report: International comparisons. 2020. https://adr.usrds.org/2020/end-stage-renal-disease/11-international-comparisons (accessed Mar 01, 2022).
114. Wong LY, Liew AST, Lim CK, Vathsala A, Toh MPHS. Projecting the Burden of Chronic Kidney Disease in a Developed Country and Its Implications on Public Health. *Int J Nephrol* 2018; **2018**: 5196285.
115. Myśliwiec M, et al. Chronic renal failure. 2021. https://www.mp.pl/pacjent/nefrologia/choroby/chorobyudoroslych/51919,przewlekla-niewydolnosc-nerek (accessed Jul 01, 2022). [Polish.]
116. National Health Fund (NHF). Chronic kidney disease. 2020. https://ezdrowie.gov.pl/5668 (accessed Jul 01, 2022). [Polish.]
117. Dębska-Ślizień A, Bolesław R, Piotr J, et al. Current Status of Renal Replacement Therapy in Poland in 2020. *Nephrol Dial Pol* 2021; **25**: 7–20.
118. MPZ Kardiologia. Health needs map in the field of cardiology for the Masovian Voivodeship. 2018. https://mpz.mz.gov.pl/wp-content/uploads/2019/06/mpz_kardiologia_mazowieckie.pdf (accessed Jul 01, 2022). [Polish.]
119. Spitalul Clinic de Nefrologie Carol Davila. Registrul Renal Român. 2022. https://www.spcaroldavila.ro/activitati/registrul-renal-roman/ (accessed Aug 01, 2022). [Romanian.]
120. Registrul Renal Român. Raportul Registrului Renal Româ n 2019–2020. 2020. http://registrulrenal.ro/resurse_statistica/Raport_RRR_2019_2020.pdf. (accessed Feb 01, 2023).
121. European Renal Association. Annual Reports. 2017–2019. 2019–2022. https://www.era-online.org/en/registry/publications/annual-reports/. (accessed Feb 01, 2023).
122. Spitalul Clinic de Nefrologie Carol Davila. Olnavi tratați și aparate de dializă pe județe 2017. 2017. https://www.spcaroldavila.ro/wp-content/uploads/2015/09/Raport_2017_Jud.pdf (accessed Aug 01, 2022). [Romanian.]
123. Ahmed HG, Ginawi IA, Al-hazimi AM. Prevalence estimates of chronic kidney disease in Hail Region, KSA: in a comprehensive survey. *Int J Sci Res* 2014; **3**: 252–6.
124. Al-Attar, B. Renal replacement therapy in the Kingdom of Saudi Arabia. *Saudi J Kidney Dis Transpl* 2021; **32**: 1188–1200.
125. Saudi Center for Organ Transplantation. Annual Report for Organ Transplantation in Kingdom of Saudi Arabia 2019. 2019. https://newscot.mosandah.com.sa/wp-content/uploads/2020/06/Report_Ar_F_9-Annual-Report-2019-En.pdf (accessed Jun 01, 2022).
126. Benjamin EJ, Munter P, Alonso A, et al. Heart Disease and Stroke Statistics-2019 Update: A Report From the American Heart Association. *Circulation* 2019; **139**: e56–528.
127. Mozaffarian D, Benjamin EJ , Go AS, et al. Heart disease and stroke statistics-2015 update: A report from the American Heart Association. *Circulation* 2015; **131**: e29–322.

128. Alhazzani AA, Mahfouz AA, Abolyazid AY, et al. Study of Stroke Incidence in the Aseer Region, Southwestern Saudi Arabia. *Int J Environ Res Public Health* 2018; **15**: 215.
129. Singapore Health Promotion Board. Singapore Renal Registry Annual Report 2018. 2020. https://www.nrdo.gov.sg/docs/librariesprovider3/default-document-library/singapore-renal-registry-annual-report-2018.pdf (accessed Dec 01, 2022).
130. Singapore Health Promotion Board. Singapore Myocardial Infarction Registry Annual Report 2018. 2020. https://www.nrdo.gov.sg/docs/librariesprovider3/default-document-library/smir-web-report-2018.pdf?sfvrsn=ccde799a_0 (accessed Dec 01, 2022).
131. Park JI, Baek H, Jung HH. Prevalence of Chronic Kidney Disease in Korea: the Korean National Health and Nutritional Examination Survey 2011–2013. *J Korean Med Sci* 2016; **31**: 915–23.
132. Kim S, Lim CS, Han DC, et al. The prevalence of chronic kidney disease (CKD) and the associated factors to CKD in urban Korea: a population-based cross-sectional epidemiologic study. *J Korean Med Sci* 2009; **24**: S11–21.
133. Korean Society of Nephrology. Current renal replacement therapy in Korea. Insan Memorial Dialysis Registry 2018. http://www.ksn.or.kr/rang_board/inc/download.php?nocount=1&code=sinchart_eng&num=2069 (accessed Nov 01, 2021).
134. Kim Y-S, Jin D-C. Global Dialysis Perspective: Korea. *Kidney360* 2020; **1**: 52–7.
135. Park S, Kim M, Kim JE, et al. Characteristics of kidney transplantation recipients over time in South Korea. *Korean J Int Med* 2020; **35**: 1457–67.
136. NHIS. Korea National Health Interview Survey 2018. 2018. http://webcache.googleusercontent.com/search?q=cache:UN9mbgDSNNEJ:www.alio.go.kr/download.dn%3FfileNo%3D2430456+&cd=16&hl=en&ct=clnk&gl=sg (accessed Nov 01, 2021).
137. Lee JH, Lim NK, Cho MC, Park HY. Epidemiology of Heart Failure in Korea: Present and Future. *Korean Circ J* 2016; **46**: 658–64.
138. Kim RB, Kim HS, Kang DR, et al. The Trend in Incidence and Case-fatality of Hospitalized Acute Myocardial Infarction Patients in Korea, 2007 to 2016. *J Korean Med Sci* 2019; **34**: e322.
139. Korea Disease Control Prevention Agency. Stroke Epidemiology Report. 2018. http://www.cdc.go.kr/board/board.es?mid=a20602010000&bid=0034&list_no=365123&act=view (accessed Apr 01, 2022). [Korean]
140. Instituto Nacional de Estadistica. Death risk by age. 2020. https://www.ine.es/jaxiT3/Tabla.htm?t=27153 (accessed May 01, 2022).
141. Svenskt Njurregister. Svenskt njurregister årsrapport 2021. 2021. https://www.medscinet.net/snr/rapporterdocs/SNR_arsrapport%202021_webversion .pdf (accessed Oct 01, 2022). [Swedish.]
142. Njurförbundet. Njurvårdsrapport 2020. 2021. https://njurforbundet.se/wp-content/uploads/2020/08/Njurv%C3%A5rdsrapport2020.pdf (accessed Oct 01, 2022). [Swedish.]
143. Kweon S, Kim Y, Jang MJ, et al. Data Resource Profile: The Korea National Health and Nutrition Examination Survey (KNHANES). *Int J Epidemiol* 2014; **43**(1): 69–77.
144. Taiwan Society of Nephrology, Annual report on kidney disease in Taiwan. 2019. [Data on file].
145. Lin CC, Liu CS, Li TC, Chen CC, Li CI, Lin WY. Microalbuminuria and the metabolic syndrome and its components in the Chinese population. *Eur J Clin Invest* 2007; **37**(10): 783–90.
146. Han JS, Lee MJ, Park KS, et al. Albuminuria as a Risk Factor for Anemia in Chronic Kidney Disease: Result from the KoreaN Cohort Study for Outcomes in Patients With Chronic Kidney Disease (KNOW-CKD). *PLoS One* 2015; **10**(10): e0139747.
147. Ministry of Health and Welfare, Statistics of chronic diseases in the population over 18 years of age (stratified by diseases). 2013. https://dep.mohw.gov.tw/dos/cp-1720-7333-113.html (accessed May 01, 2022).
148. Wang CL, Kuo CF, Yeh YH, Hsieh MY, Kuo CT, Chang SH. Familial aggregation of myocardial infarction and coaggregation of myocardial infarction and autoimmune disease: a nationwide population-based cross-sectional study in Taiwan. *BMJ Open* 2019; **9**(3): e023614.
149. Siqueira CADS, de Souza DLB. Reduction of mortality and predictions for acute myocardial infarction, stroke, and heart failure in Brazil until 2030. *Sci Rep* 2020; **10**(1): 17856.
150. Perkovic V, et al. High prevalence of chronic kidney disease in Thailand. *Kidney Int* 2008; **73**: 473–9.

151. Nephrology Society of Thailand. Thailand Renal Replacement Therapy. 2020. https://www.nephrothai.org/wp-content/uploads/2021/10/Final-TRT-report-2020.pdf (accessed May 01, 2022).
152. Süleymanlar G, Utaş C, Arinsoy T, et al. A population-based survey of Chronic REnal Disease in Turkey--the CREDIT study. *Nephrol Dial Transplant* 2011; **26**(6): 1862–71.
153. GHDx. Turkey Registry of Nephrology, Dialysis and Transplantation Renal Replacement Therapy Data 2014 – ERA-EDTA. 2021. http://ghdx.healthdata.org/record/turkey-registry-nephrology-dialysis-and-transplantation-renal-replacement-therapy-data-2014. (accessed Aug 01, 2022).
154. Díaz-Guzmán J, Bermejo-Pareja F, Benito-León J, et al. Prevalence of stroke and transient ischemic attack in three elderly populations of central Spain. *Neuroepidemiology* 2008; **30**(4): 247–53.
155. Centers for Disease Control and Prevention (CDC). National Health and Nutrition Examination Survey (NHANES). 2018. https://www.cdc.gov/nchs/nhanes/index.htm (accessed Jun 01, 2022).
156. Centers for Disease Control and Prevention (CDC). Health expenditures. 2021. https://www.cdc.gov/nchs/fastats/health-expenditures.htm (accessed May 26, 2022).

Table S3 references

1. Australian Bureau of Statistics. Australian Health Survey 2011–2012. 2012. https://www.abs.gov.au/statistics/health/health-conditions-and-risks/australian-health-survey-biomedical-results-chronic-diseases/latest-release#chronic-kidney-disease (accessed Sep 10, 2021).
2. Australian Institute of Health and Welfare (AIHW). Chronic kidney disease prevalence among Australian adults over time. 2018. https://www.aihw.gov.au/getmedia/cbfc53e4-c6fc-4a8e-9a7f-e5dfa38ae10c/aihw-cdk-6.pdf.aspx?inline=true (accessed Sep 10, 2021).
3. Bikbov B, Purcell CA, Levey AS, et al. Global, regional, and national burden of chronic kidney disease, 1990–2017: a systematic analysis for the Global Burden of Disease Study 2017. *Lancet* 2020; **395**(10225): 709–33.
4. AIHW. Chronic kidney disease, What is chronic kidney disease? https://www.aihw.gov.au/reports/chronic-kidney-disease/chronic-kidney-disease/contents/what-is-chronic-kidney-disease (accessed Aug 31, 2021).
5. Conrad N, Judge A, Tran J, et al. Temporal trends and patterns in heart failure incidence: a population-based study of 4 million individuals. *Lancet* 2018; **391**(10120): 572–80.
6. Meisinger C, Döring A, Löwel H. Chronic kidney disease and risk of incident myocardial infarction and all-cause and cardiovascular disease mortality in middle-aged men and women from the general population. *Eur Heart J* 2006; **27**(10): 1245–50.
7. Weiner DE, Tighiouart H, Amin MG, et al. Chronic kidney disease as a risk factor for cardiovascular disease and all-cause mortality: a pooled analysis of community-based studies. *J Am Soc Nephrol* 2004; **15**(5): 1307–15.
8. The Heart Failure Policy Network. Heart failure policy and practice in Europe: Belgium. www.hfpolicynetwork.org/ (accessed Sep 9, 2021).
9. The World Bank. World Development Report 1993. 1993. https://openknowledge.worldbank.org/handle/10986/5976 (accessed Sep 9, 2021).
10. Barreto SM, Ladeira RM, Duncan BB, et al. Chronic kidney disease among adult participants of the ELSA-Brasil cohort: association with race and socioeconomic position. *J Epidemiol Community Health* 2016; **70**(4): 380–9.
11. Piccolli AP, do Nascimento MM, Riella MC. Prevalence of chronic kidney disease in a population in southern Brazil (Pro-Renal Study). *J Bras Nefrol* 2017; **39**(4): 384–90.
12. de Souza W, de Abreu LC, da Silva LG, Bezerra IMP. Incidence of chronic kidney disease hospitalisations and mortality in Espírito Santo between 1996 to 2017. *PLoS One* 2019; **14**(11): e0224889.
13. Stevens B, Pezzullo L, Verdian L, Tomlinson J, George A, Bacal F. The Economic Burden of Heart Conditions in Brazil. *Arq Bras Cardiol* 2018; **111**(1): 29.
14. Bensenor IM, Goulart AC, Szwarcwald CL, Vieira MLFP, Malta DC, Lotufo PA. Prevalence of stroke and associated disability in Brazil: National Health Survey - 2013. *Arq Neuropsiquiatr* 2015; **73**(9): 746–50.
15. Arora P , Vasa P, Brenner D, et al. Prevalence estimates of chronic kidney disease in Canada: results of a nationally representative survey. *CMAJ* 2013; **185**(9): E417–23.
16. Bello AK, Ronksley PE, Tangri N, et al. Prevalence and Demographics of CKD in Canadian Primary Care Practices: A Cross-sectional Study. *Kidney Int Rep* 2019; **4**(4): 561–70.
17. Redfors B, Angerås O, Råmunddal T, et al. Trends in Gender Differences in Cardiac Care and Outcome After Acute Myocardial Infarction in Western Sweden: A Report From the Swedish Web System for

Enhancement of Evidence-Based Care in Heart Disease Evaluated According to Recommended Therapies (SWEDEHEART). *J Am Heart Assoc* 2015; **4**(7): e001995.
18. Statista. Prevalence of diagnosed ischemic heart disease and acute myocardial infarction in Canada from 2000 to 2013. 2018. https://www.statista.com/statistics/881873/diagnosed-heart-disease-prevalence-canada/ (accessed Sep 1, 2021).
19. Mace-Brickman T, Eddeen AB, Carrero J-J, et al. The Risk of Stroke and Stroke Type in Patients With Atrial Fibrillation and Chronic Kidney Disease. *Can J Kidney Health Dis* 2019; **6**: 2054358119892372.
20. Wang F, He K, Wang J, et al. Prevalence and Risk Factors for CKD: A Comparison Between the Adult Populations in China and the United States. *Kidney Int Rep* 2018; **3**(5): 1135–43.
21. Zhang L, Wang F, Wang L, et al. Prevalence of chronic kidney disease in China: a cross-sectional survey. *Lancet* 2012; **379**(9818): 815–22.
22. Zhang L, Zhao M-H, Zuo L, et al. China Kidney Disease Network (CK-NET) 2016 Annual Data Report. *Kidney Int Suppl* 2020; **10**(2): e97.
23. Pradilla G, Pardo CA, Daza JS, Zúñiga A. Neuroepidemiología en Colombia: estudios en 16.032 individuos. *Acta Neurol Colomb* 1995; **11**: 129–33. [Spanish.]
24. Thomsen RW, Nicolaisen SK, Hasvold P, et al. Elevated potassium levels in patients with chronic kidney disease: occurrence, risk factors and clinical outcomes-a Danish population-based cohort study. *Nephrol Dial Transplant* 2018; **33**(9): 1610–20.
25. Kampmann JD, Goya Heaf J, Mogensen CB, Mickley H, Brandt F. Kidney Disease Cohort (KidDiCo) of Southern Denmark: Design, Coverage, Generalizability and Implications for Use. *Clin Epidemiol* 2021; **13**: 971–80.
26. Vestergaard SV, Heide-Jørgensen U, Birn H, Christiansen CF. Effect of the Refitted Race-Free eGFR Formula on the CKD Prevalence and Mortality in the Danish Population. *Clin J Am Soc Nephrol* 2022; **17**(3): 426–8.
27. Villain C, Metzger M, Combe C, et al. Prevalence of atheromatous and non-atheromatous cardiovascular disease by age in chronic kidney disease. *Nephrol Dial Transplant* 2020; **35**(5): 827–36.
28. Brück K, Stel VS, Gambaro G, et al. CKD Prevalence Varies across the European General Population. *J Am Soc Nephrol* 2016; **27**(7): 2135–47.
29. Bommer J. Prevalence and socio-economic aspects of chronic kidney disease. *Nephrol Dial Transplant* 2002; **17**(Suppl 11): 8–12.
30. Beck H, Titze SI, Hübner S, et al. Heart Failure in a Cohort of Patients with Chronic Kidney Disease: The GCKD Study. *PLoS One* 2015; **10**(4): e0122552.
31. Gößwald A, Schienkiewitz A, Nowossadeck E, Busch MA. [Prevalence of myocardial infarction and coronary heart disease in adults aged 40-79 years in Germany: results of the German Health Interview and Examination Survey for Adults (DEGS1)]. *Bundesgesundheitsblatt Gesundheitsforschung Gesundheitsschutz* 2013; **56**(5–6): 650–5. [German.]
32. The Burden of Stroke in Germany: Stroke Alliance for Europe 2017. 2017. https://www.safestroke.eu/wp-content/uploads/2017/12/SAFE_STROKE_GERMANY.pdf (accessed Sep 10, 2021).
33. Loutradis C, Tolika P, Skodra A, Avdelidou A, Sarafidis PA. Prevalence of Hyperkalemia in Diabetic and Non-Diabetic Patients with Chronic Kidney Disease: A Nested Case-Control Study. *Am J Nephrol* 2015; **42**(5): 351–60.
34. National Health Insurance Fund Administration (NHIFA). Hungarian National Statistics. 2019. https://www.ksh.hu/?lang=en (accessed Apr 06, 2023).
35. Singh A, Farag Y, Mittal B, et al. Epidemiology and risk factors of chronic kidney disease in India - results from the SEEK (Screening and Early Evaluation of Kidney Disease) study. *BMC Nephrol* 2013; **14**: 114.
36. Huffman M, Prabhakaran D. Heart failure: Epidemiology and prevention in India. *National Med J India* 2010; **23**(5): 283.
37. Pandian JD, Sudhan P. Stroke Epidemiology and Stroke Care Services in India. *J Stroke* 2013; **15**(3): 128.
38. Ministry of Health Report 2013. https://www.gov.il/BlobFolder/reports/health-2013/he/files_publications_units_ICDC_health2013.pdf (accessed Nov 17, 2021).
39. The Burden of Stroke in Israel: Stroke Alliance for Europe 2017. 2018 https://www.safestroke.eu/wp-content/uploads/2017/12/SAFE_STROKE_ISRAEL.pdf (accessed Nov 17, 2021).
40. de Nicola L, Donfrancesco C, Minutolo R, et al. Prevalence and cardiovascular risk profile of chronic kidney disease in Italy: results of the 2008–12 National Health Examination Survey. *Nephrol Dial Transplant* 2015; **30**(5): 806–14.
41. Capuano V, Lamaida N, Borrelli MI, et al. [Chronic kidney disease prevalence and trends (1998-2008) in an area of southern Italy. The data of the VIP project]. *G Ital Nefrol* 2012; **29**(4): 445–51. [Italian.]
42. Navarra S, Solini A, Baroni MG, Frova L, Grande E. A long-term nationwide study on chronic kidney disease-related mortality in Italy: trends and associated comorbidity. *J Nephrol* 2022; **35**(2): 505–15.

43. Barbieri MA, Rottura M, Cicala G, et al. Chronic Kidney Disease Management in General Practice: A Focus on Inappropriate Drugs Prescriptions. *J Clin Med* 2020; **9**(5): 1346.
44. Giampaoli S, Palmieri L, Donfrancesco C, Lo Noce C, Pilotto L, Vanuzzo D. Cardiovascular health in Italy. Ten-year surveillance of cardiovascular diseases and risk factors: Osservatorio Epidemiologico Cardiovascolare/Health Examination Survey. *Eur J Prev Cardiol* 2015; **22**(2 Suppl): 9–37.
45. The Burden of Stroke in Italy: Stroke Alliance for Europe 2017. 2018. https://www.safestroke.eu/wp-content/uploads/2017/12/SAFE_STROKE_ITALY.pdf (accessed Sep 24, 2021).
46. Imai E, Horio M, Iseki K, et al. Prevalence of chronic kidney disease (CKD) in the Japanese general population predicted by the MDRD equation modified by a Japanese coefficient. *Clin Exp Nephrol* 2007; **11**(2): 156–63.
47. Nagata M, Ninomiya T, Doi Y, et al. Trends in the prevalence of chronic kidney disease and its risk factors in a general Japanese population: The Hisayama Study. *Nephrol Dial Transplant* 2010; **25**: 2557–64.
48. Takeuchi M, Shinkawa K, Yanagita M, Kawakami K. Prevalence, recognition and management of chronic kidney disease in Japan: population-based estimate using a healthcare database with routine health checkup data. *Clin Kidney J* 2021; **14**(10): 2197–202.
49. Tanaka K, Watanabe T, Takeuchi A, et al. Cardiovascular events and death in Japanese patients with chronic kidney disease. *Kidney Int* 2017; **91**(1): 227–34.
50. Ninomiya T, Perkovic V, de Galan BE, et al. Albuminuria and Kidney Function Independently Predict Cardiovascular and Renal Outcomes in Diabetes. *J Am Soc Nephrol* 2009; **20**(8): 1813–21.
51. Sundström J, Bodegard J, Bollmann A, et al. Prevalence, outcomes, and cost of chronic kidney disease in a contemporary population of 2·4 million patients from 11 countries: The CaReMe CKD study. *Lancet Reg Health Eur* 2022; **20**: 100438.
52. Morales DD, et al. National Nutrition and Health Survey (NNHeS): 2003–2004 cardiovascular disease-related results and implications. 2005. https://www.herdin.ph/index.php/component/herdin/?view=research&cid=37903 (accessed Sep 10, 2021).
53. Tumanan-Mendoza BA, Mendoza VL, Santos AAAB, et al. Epidemiologic burden of hospitalisation for congestive heart failure among adults aged ≥19 years in the Philippines. *Heart Asia* 2017; **9**(1): 76–80.
54. Sy RG, Morales DD, Dans AL, et al. Prevalence of Atherosclerosis-Related Risk Factors and Diseases in the Philippines. *J Epidemiol* 2012; **22**(5): 440–7.
55. Puch-Walczak A, Bandosz P, Grodzicki T, et al. Prevalence of self-reported heart failure in the adult Polish population: results of the NATPOL 2011 study. *Pol Arch Intern Med* 2022; **132**(4): 16184.
56. The Burden of Stroke in Poland: Stroke Alliance for Europe 2017. 2018. https://www.safestroke.eu/wp-content/uploads/2017/12/SAFE_STROKE_POLAND.pdf (accessed Jul 01, 2022).
57. AbuRuz ME, Alaloul F, Saifan A, Masa'Deh R, Abusalem S. Quality of Life for Saudi Patients With Heart Failure: A Cross-Sectional Correlational Study. *Glob J Health Sci* 2016; **8**(3): 49–58.
58. Memon I, Abu-Shaheen A, Heena H, Al-Tannir M. Point prevalence study for stroke in Saudi Arabia: A cross-sectional survey. *Saudi J Health Sci* 2019; **8**(2): 93–7.
59. Wong LY, Liew AST, Weng WT, Lim CK, Vathsala A, Toh MPHS. Projecting the Burden of Chronic Kidney Disease in a Developed Country and Its Implications on Public Health. *Int J Nephrol* 2018; **2018**: 5196285.
60. Picco L, Subramaniam M, Abdin E, Vaingankar JA, Chong SA. Prevalence and correlates of heart disease among adults in Singapore. *Asian J Psychiatr* 2016; **19**: 37–43.
61. Teh WL, Abdin E, Vaingankar JA, et al. Prevalence of stroke, risk factors, disability and care needs in older adults in Singapore: results from the WiSE study. *BMJ Open* 2018; **8**(3): e020285.
62. Lee M-J, Ha KH, Kim DJ, Park I. Trends in the Incidence, Prevalence, and Mortality of End-Stage Kidney Disease in South Korea. *Diabetes Metab J* 2020; **44**(6): 933–7.
63. Stroke Statistics in Korea 2018: A Report from the Epidemiology Research Council of the Korean Stroke Society. Korean Stroke Society 2018 https://www.stroke.or.kr/bbs/skin/notice_popup/download.php?code=epidemic1&number=5305 (accessed Oct 20, 2021).
64. Gorostidi M, Sánchez-Martínez M, Ruilope LM, et al. Chronic kidney disease in Spain: Prevalence and impact of accumulation of cardiovascular risk factors. *Nefrología (Engl Ed)* 2018; **38**(6): 606–15.
65. González AO, de Francisco A, Gayoso P, García F. Prevalence of chronic renal disease in Spain: Results of the EPIRCE study. *Nefrología* 2010; **30**(1): 78–86.
66. Martínez-Castelao A, Górriz JL, Portolés JM, et al. Baseline characteristics of patients with chronic kidney disease stage 3 and stage 4 in Spain: the MERENA observational cohort study. *BMC Nephrol* 2011; **12**: 53.
67. The Burden of Stroke in Spain: Stroke Alliance for Europe 2017. 2018. https://www.safestroke.eu/wp-content/uploads/2017/12/SAFE_STROKE_SPAIN.pdf (accessed Sep 29, 2021).
68. Lindmark K, et al. Epidemiology of heart failure and trends in diagnostic work-up: a retrospective, population-based cohort study in Sweden. *Clin Epidemiol* 2019; **11**: 231–44.

69. Statista. Number strokes in Sweden from 2010 to 2020. 2022. https://www.statista.com/statistics/950406/number-strokes-in-sweden/ (accessed Feb 27, 2023).
70. Tsai M-H, Hsu C-Y, Lin M-Y, et al. Incidence, Prevalence, and Duration of Chronic Kidney Disease in Taiwan: Results from a Community-Based Screening Program of 106,094 Individuals. *Nephron* 2018; **140**(3): 175–84.
71. Lee W-C, Lee Y-T, Li L-C, et al. The Number of Comorbidities Predicts Renal Outcomes in Patients with Stage 3–5 Chronic Kidney Disease. *J Clin Med* 2018; **7**(12): 493.
72. Huang Z-S, Chiang T-L, Lee T-K. Stroke Prevalence in Taiwan. *Stroke* 1997; **28**(8): 1579–84.
73. Reyes EB, Ha J-W, Firdaus I, et al. Heart failure across Asia: Same healthcare burden but differences in organization of care. *Int J Cardiol* 2016; **223**:163–7
74. Sakboonyarat B, Rangsin R. Prevalence and associated factors of ischemic heart disease (IHD) among patients with diabetes mellitus: a nation-wide, cross-sectional survey. *BMC Cardiovasc Disord* 2018; **18**(1): 151.
75. Suwanwela NC. Stroke Epidemiology in Thailand. *J Stroke* 2014; **16**(1): 1–7.
76. Süleymanlar G, Utaş C, Arinsoy T, et al. A population-based survey of Chronic REnal Disease in Turkey-the CREDIT study. *Nephrol Dial Transplant* 2011; **26**(6): 1862–71.
77. Değertekin M, Erol Ç, Ergene O, et al. Türkiye'deki kalp yetersizliği prevalansi ve öngördürücüleri: HAPPY çalişmasi. *Türk Kardiyol Dern Arş* 2012; **40**(4): 298–308. [Turkish.]
78. Kılıç S, Aydın G, Çoner A, et al. Prevalence and clinical profile of patients with myocardial infarction with non-obstructive coronary arteries in Turkey (MINOCA-TR): A national multi-center, observational study. *Anatol J Cardiol* 2020. **23**(3): 176–182.
79. Börü ÜT, Kulualp AŞ, Tarhan ÖF, et al. Stroke prevalence among the Turkish population in a rural area of Istanbul: A community-based study. *SAGE Open Med* 2018; **6**: 2050312118797565.
80. Xie Y, Bowe B, Mokdad AH, et al., Analysis of the Global Burden of Disease study highlights the global, regional, and national trends of chronic kidney disease epidemiology from 1990 to 2016. *Kidney Int* 2018; **94**(3): 567–81.
81. Public Health England. Chronic Kidney Disease (CKD) prevalence model. 2014. https://assets.publishing.service.gov.uk/government/uploads/system/uploads/attachment_data/file/612303/ChronickidneydiseaseCKDprevalencemodelbriefing.pdf (accessed Jul 14, 2022).
82. Hounkpatin HO, Harris S, Fraser SDS, et al. Prevalence of chronic kidney disease in adults in England: comparison of nationally representative cross-sectional surveys from 2003 to 2016. *BMJ Open* 2020; **10**(8): e038423.
83. British Heart Foundation. Heart statistics - Cardiovascular Disease Statistics 2017. https://www.bhf.org.uk/what-we-do/our-research/heart-statistics/heart-statistics-publications/cardiovascular-disease-statistics-2017 (accessed Aug 09, 2022).
84. Jayaraj JC, Davatyan K, Subramanian SS, Priya J. Epidemiology of Myocardial Infarction. 2019. https://www.intechopen.com/chapters/59778 (accessed Sep 7, 2021).
85. Health Survey for England 2017 Cardiovascular diseases. 2018. www.statisticsauthority.gov.uk/assessment/code-of-practice (accessed Sep 7, 2021).
86. United States Renal Data System. Chapter 1: CKD in the General Population. 2018. https://www.usrds.org/media/1723/v1_c01_genpop_18_usrds.pdf (accessed Sep 24, 2021).
87. National Institute of Diabetes and Digestive and Kidney Diseases. Kidney Disease Statistics for the United States. 2015. https://www.niddk.nih.gov/health-information/health-statistics/kidney-disease (accessed Sep 10, 2021).
88. Bowe B, Xie Y, Li T, et al. Changes in the US Burden of Chronic Kidney Disease From 2002 to 2016: An Analysis of the Global Burden of Disease Study. *JAMA Netw Open* 2018; **1**(7): e184412.
89. Cheung AK, Sarnak MJ, Yan G, et al. Cardiac diseases in maintenance hemodialysis patients: Results of the HEMO Study. *Kidney Int* 2004; **65**(6): 2380–9.
90. Komanduri S, Jadhao Y, Guduru SS, Cheriyath P, Wert Y. Prevalence and risk factors of heart failure in the USA: NHANES 2013 – 2014 epidemiological follow-up study. *J Community Hosp Intern Med Perspect* 2017; **7**(1): 15–20.
91. Bradley SM, Borgerding JA, Wood GB, Maynard C, Fihn SD. Incidence, risk factors, and outcomes associated with in-hospital acute myocardial infarction. *JAMA Netw Open* 2019; **2**(1): e187348.
92. Benjamin EJ, Virani SS, Callaway CW, et al. Heart Disease and Stroke Statistics–2018 Update: A Report From the American Heart Association. *Circulation* 2018; **137**(12): e67–492.
